# The Korea Biobank Array: Design and Identification of Coding Variants Associated with Blood Biochemical Traits

**DOI:** 10.1038/s41598-018-37832-9

**Published:** 2019-02-04

**Authors:** Sanghoon Moon, Young Jin Kim, Sohee Han, Mi Yeong Hwang, Dong Mun Shin, Min Young Park, Yontao Lu, Kyungheon Yoon, Hye-Mi Jang, Yun Kyoung Kim, Tae-Joon Park, Dae Sub Song, Jae Kyung Park, Jong-Eun Lee, Bong-Jo Kim

**Affiliations:** 1 Division of Genome Research, Center for Genome Science, National Institute of Health, Chungcheongbuk-do, 28159 Republic of Korea; 2 Division of Epidemiology and Health Index, Center for Genome Science, National Institute of Health, Chungcheongbuk-do, 28159 Republic of Korea; 3 DNA link, Incorporated, Seoul, 03759 Republic of Korea; 4 Affymetrix, Inc, Santa Clara, USA

## Abstract

We introduce the design and implementation of a new array, the Korea Biobank Array (referred to as KoreanChip), optimized for the Korean population and demonstrate findings from GWAS of blood biochemical traits. KoreanChip comprised >833,000 markers including >247,000 rare-frequency or functional variants estimated from >2,500 sequencing data in Koreans. Of the 833 K markers, 208 K functional markers were directly genotyped. Particularly, >89 K markers were presented in East Asians. KoreanChip achieved higher imputation performance owing to the excellent genomic coverage of 95.38% for common and 73.65% for low-frequency variants. From GWAS (Genome-wide association study) using 6,949 individuals, 28 associations were successfully recapitulated. Moreover, 9 missense variants were newly identified, of which we identified new associations between a common population-specific missense variant, rs671 (p.Glu457Lys) of *ALDH2*, and two traits including aspartate aminotransferase (P = 5.20 × 10^−13^) and alanine aminotransferase (P = 4.98 × 10^−8^). Furthermore, two novel missense variants of *GPT* with rare frequency in East Asians but extreme rarity in other populations were associated with alanine aminotransferase (rs200088103; p.Arg133Trp, P = 2.02 × 10^−9^ and rs748547625; p.Arg143Cys, P = 1.41 × 10^−6^). These variants were successfully replicated in 6,000 individuals (P = 5.30 × 10^−8^ and P = 1.24 × 10^−6^). GWAS results suggest the promising utility of KoreanChip with a substantial number of damaging variants to identify new population-specific disease-associated rare/functional variants.

## Introduction

In the last decade, numerous genome-wide association studies (GWAS) have been conducted to discover genetic factors associated with complex diseases and related traits^1–4^. For genomic studies, next-generation sequencing (NGS) and single nucleotide polymorphism (SNP) genotyping arrays have been widely used. NGS technologies such as whole-genome sequencing (WGS) and whole-exome sequencing (WES) have been frequently used to investigate Mendelian disorders^5,6^. Despite the extensive decline of sequencing costs, these methods are still difficult to apply to GWAS, which requires large sample sizes for complex traits^5^. Because most traditional SNP genotyping arrays are less expensive than NGS, SNP arrays have been used to identify variants with moderate to high allele frequencies in GWAS with large sample sizes^7^. However, widely used commercial arrays are designed for multiethnic populations. This causes lower genomic coverage for populations with Asian and African ancestry than for populations with European ancestry^8^. Furthermore, most commercial arrays designed before 2010 do not include the numerous variants newly discovered by NGS technologies that are of special interest^9^. Therefore, alternative genomic analysis tools are warranted to bridge the gap between SNP arrays and NGS in large-scale genomic studies of complex traits.

Recently, to address these limitations, several SNP genotyping arrays optimized for each purpose were introduced as next-generation genome-wide association tools (hereafter referred to as next-generation arrays)^10–15^. For example, the Illumina HumanExome BeadChip (Illumina exome array) was designed to identify associations between exonic rare variants and various traits. In addition, customized genotyping arrays, such as the Axiom UK Biobank array, Japonica array, and Kadoorie biobank array were designed to contain optimal sets of tagging variants for specific populations to determine genetic factors influencing complex diseases in participants of a large-scale biobank or for other specific purposes^10–15^. More recently, two different commercial chip companies have launched the Global Screening Array (GSA) (http://www.illumina.com/techniques/microarrays) and Precision Medicine Research Array (PMRA) (http://www.affymetrix.com/catalog) for global screening of diverse populations, respectively. Indeed, about a half-million genome datasets generated using the Axiom UK Biobank array introduced a new era of GWAS and provided unprecedented association mapping power to discover several hundred novel loci associated with various traits^14^.

Over the last decade, Korean Association REsource (KARE) data, comprising genotypes obtained with the Affymetrix Genome-Wide Human SNP Array 5.0 (Affymetrix 5.0) using the Ansan and Ansung cohort samples, have contributed to the discovery of numerous genetic variants associated with complex diseases^16–20^. However, these data have limitations of low genomic coverage and absence of functional variants recently discovered from NGS data. Moreover, a significant number of markers have not been available for further study because these variants are monomorphic in the Korean population^16,17^.

To address these issues and establish a large-scale Korean genomic data repository, the Korea Biobank Array Project was initiated in 2014 to implement a customized Korean genome structure-based array with high genomic coverage and abundant functional variants of low to rare frequency. Previously, the Korean National Institute of Health recruited more than 210,000 participants via the Korean Genome and Epidemiology Study (KoGES)^21^. All participants aged 40–69 years were examined through epidemiological surveys, physical examinations, and laboratory tests. All KoGES samples, with informed consent, will be genotyped with KoreanChip and made publicly available.

Here, we introduce the Korea Biobank Array Project and demonstrate the design, implementation, and performance of the Korea biobank array (hereafter referred to as KoreanChip). Furthermore, we report findings of blood biochemical trait-associated variants from the first phase analysis using 6,949 individuals followed by replication analysis including 6,000 individuals. As a result, 9 variants including six new missense variants of known loci, a novel association of a known variant, and two novel protective associations of a missense variant were identified for a lipid trait and a liver enzyme. Our methods and findings provide new insight into the discovery of rare or functional variants using large-scale GWAS with a well-designed next-generation array. In addition, the final biobank comprising genetic data of >210,000 individuals will serve as a valuable resource of East Asian genome information for large-scale single ethnic GWAS to determine population-specific disease-causing variants and provide scientific evidence for precision medicine.

## Results

### Array content

An SNP array including a total of 833,535 SNPs and insertion-deletion (indel) markers was designed (Supplementary Fig. S1). Detailed information for KoreanChip is summarized in Table 1. Briefly, more than 95% of the content consisted of about 600 K tagging variants for genome-wide coverage and about 208 K functional variants of mostly nonsynonymous variants retrieved from Korean sequencing data and indels. The tagging variants for autosomal chromosomes were selected using Affymetrix imputation-aware marker selection algorithms considering allele frequencies of 7.7 M common variants (minor allele frequency (MAF) ≥ 1%) in 2,576 Korean sequencing data (397 WGS and 2,179 WES)^22^. Among 208 K functional variants, approximately 149 K variants were nonsynonymous SNPs with MAF ≥ 0.1%, and about 45 K variants were nonsynonymous SNPs with MAF < 0.1%, selected only if predicted to be damaging based on the annotations from the dbNSFP database v2.4 (see Methods)^23^. Similar to the Axiom UK Biobank array, KoreanChip also included ~36 K markers of specific interest, such as the available modules in the Affymetrix Axiom Genomic Database (AxiomGD) for absorption, distribution, metabolism, and excretion of drugs (ADME); expression quantitative trait loci (eQTL); NHGRI GWAS catalog^24^; and HLA/KIR as well as mitochondrial DNA and Y chromosome (Table 1).

**Table 1:** Contents summary of KoreanChip.

Category	Number of SNPs*	Contents (%)
Tag SNPs for genome-wide coverage	600,294	72.02
Functional loci (nonsynonymous SNPs and Indels)	208,039	24.96
eQTL	16,690	2.00
HLA	6,659	0.80
Fingerprint	255	0.03
NHGRI GWAS catalog	7,811	0.94
KIR	1,544	0.19
Pharmacogenetics/ADME	1,881	0.23
Common mitochondrial DNA variants	178	0.02
Y chromosome markers	806	0.10
Total	833,535	—

*Some SNPs are overlapped among categories.

eQTL, expression Quantitative Trait Loci; HLA, Human leukocyte antigen; KIR, Killer cell immunoglobulin like receptors; ADME, Absorption, Distribution, Metabolism, and Excretion.

### Reliability and accuracy

High reliability of a genotyping platform is critical to achieve confidence in SNP data and interpretation of GWAS results^25^. To assess the reliability of KoreanChip, we measured reproducibility and accuracy. The reproducibility of KoreanChip was measured via duplicate blind comparisons, which entailed duplicating 35 samples. The genotype concordance rate between duplicates was 99.8%.

To evaluate the accuracy of KoreanChip, concordance of genotypes was assessed by comparison with that of identical genotypes from three different platforms (Supplementary Table S1): 6,949 from Affymetrix 5.0, 5,793 from the Illumina exome array, and 155 from exome sequencing. In total, 41,246, 34,683, and 90,020 variants overlapped among KoreanChip and Affymetrix 5.0, Illumina exome array, and WES data, respectively. Overall, genotype concordance was more than 99.8% for all platforms.

In KoreanChip, about 25% of the content comprised nonsynonymous functional variants, and most of them had a frequency less than 1%. It is well known that genotype calling of low-frequency (MAF < 5%) and rare variants (MAF < 1%) may produce spurious genotype calls^26,27^. Therefore, we assessed the accuracy of heterozygote genotypes since most rare alleles are carried by heterozygotes. Genotype concordances of heterozygotes were 99.5%, 99.7%, and 99.7% between KoreanChip and Affymetrix 5.0, Illumina exome array, and WES data, respectively.

### Comparison of content between KoreanChip and other SNP arrays

#### Shared content with existing arrays

Supplementary Tables S2–3 show the results of the array content comparison. As expected, the fully customized KoreanChip shared less than 25% of its content with other platforms. In the case of widely used commercial arrays such as Affymetrix 5.0, Affymetrix Genome-Wide Human SNP Array 6.0 (Affymetrix 6.0), and Illumina HumanOmni1-Quad BeadChip (Illumina Omni 1 M) that were designed based on information from the International HapMap project, KoreanChip shared 47,846, 90,057, and 123,761 markers with these platforms, respectively (Supplementary Table S2). Regarding next-generation arrays designed for specific purposes, KoreanChip shared 219,690, 238,929, 42,807, and 275,312 markers with Axiom Biobank array, Axiom UK Biobank array, Illumina exome array, and Axiom PMRA, respectively (Supplementary Table S3).

#### Nonsynonymous variants

The numbers of nonsynonymous variants are summarized in Table 2. KoreanChip contains 183,607 nonsynonymous markers (22.1%). Axiom Biobank includes the highest number of nonsynonymous markers (251,080 of 645,060 markers, 38.9%). The Illumina exome array contains 217,775 markers, showing the highest ratio of nonsynonymous markers to total markers (90.0%). In the case of Axiom PMRA (44,819 markers, 5.2%) and Illumina GSA (87,759 markers, 12.8%), these arrays have fewer nonsynonymous variant markers than the Axiom Biobank array and Illumina exome array. With regard to widely used commercial arrays, as expected, Illumina Omni 1 M contains 45,832 markers (4.3%), whereas Affymetrix 5.0 (2,179 markers, 0.4%) and Affymetrix 6.0 (4,889 markers, 0.5%) include relatively small numbers of nonsynonymous variants.

**Table 2:** Comparison of contents between KoreanChip and other genotyping platforms.

Platform	Total marker	Annotated marker^a^	Nonsyn marker^b^	ASN marker^c^
	N	N	N (%)	N (%)
Affymetrix 5.0	500,568	489,457	2,179 (0.4)	769 (0.2)
Affymetrix 6.0	934,969	892,584	4,889 (0.5)	1,750 (0.2)
Illumina Omni 1 M	1,099,726	1,066,324	45,832 (4.3)	12,516 (1.2)
Illumina Exome array	242,901	241,923	217,775 (90.0)	39,480 (16.3)
Illumina GSA	700,078	688,062	87,759 (12.8)	21,371 (3.1)
Axiom Biobank	718,212	645,060	251,080 (38.9)	46,416 (7.2)
Axiom UK Biobank	845,487	823,336	104,058 (12.6)	19,487 (2.4)
Axiom PMRA	920,744	856,797	44,819 (5.2)	6,088 (0.7)
**KoreanChip**	**833**,**535**	**829**,**635**	**183**,**607 (22**.**1)**	**89**,**413 (10**.**8)**

^a^Annotated by snpEff v4.1d based on the database of dbNSFP2.7 (functional prediction and annotation of nonsynonymous marker).

^b^Proportion of nonsynonymous markers among annotated markers.

^c^Proportion of nonsynonymous makers, if damaging effect is predicted by more than one functional annotation software, and allele frequency >0 observed in East Asian ancestry among annotated markers.

ASN, Asian; Affymetrix 5.0, Affymetrix Genome-wide Human SNP array 5.0; AFFY6.0, Affymetrix Genome-wide Human SNP array 6.0; ILLU Omni 1 M, Illumina HumanOmni1-Quad BeadChip; Illumina Exome array, Illumina HumanExome BeadChip; Illumina GSA, Illumina Global Screening Array; Axiom PMRA, Axiom Precision Medicine Research Array.

We investigated nonsynonymous variants observed in East Asian populations in each array. Axiom Biobank (251,080 markers) and Illumina exome arrays (217,775 markers) contained more nonsynonymous variants than KoreanChip (183,607 markers). However, the numbers of nonsynonymous variants presented in East Asians with MAF >0 (from 1000 Genomes Project) were 46,416 and 39,480, respectively; these numbers are relatively smaller than that of KoreanChip (89,413 markers) (Table 2).

### Genomic coverage

Genomic coverage, the proportion of genomic variation captured, is an important factor for association mapping^28^. In this study, we assessed the imputation-based genomic coverage (See Methods) of KoreanChip and 8 other commercial genotyping platforms. On the basis of the frequency from 504 genome datasets from the East Asian population of 1000 Genomes Project Phase 3, we set 8,699,350 variants with MAF ≥ 1% in East Asians as the reference to estimate the genomic coverage for common variants. Among 8,699,350 variants, 6,488,804 were common variants (MAF ≥ 5%), and 2,211,346 were low-frequency variants (MAF 1–5%). Table 3 summarizes the results of the genomic coverage comparison between KoreanChip and other commercial arrays. The estimated genomic coverage of KoreanChip was 95.38% for variants with MAF ≥ 5%, which was the highest among all arrays. In particular, KoreanChip showed the highest genomic coverage for low-frequency variants (73.65%). Illumina Omni 1 M showed 94.10% and 66.01% genomic coverage for common and low-frequency variants, respectively. Affymetrix 6.0 showed 91.67% genomic coverage for common and 61.23% for low-frequency variants. As expected, the estimated genomic coverage of Affymetrix 5.0 was relatively lower than that of other arrays (84.78% for common and 51.23% for low-frequency variants). Because we only genotyped 96 samples for Axiom PMRA, Axiom UK Biobank, Axiom Biobank, and Illumina GSA (Table 3), we randomly selected 96 samples from KoreanChip data, and the imputation-based genomic coverage was calculated. Although a relatively small number of samples may cause reduced imputation performance compared with that of a larger sample size^29^, KoreanChip showed the best imputation coverage for common (95.24%) and low-frequency variants (68.22%). Supplementary Figs S2–3 demonstrate plots of estimated genomic coverage by each chromosome.

**Table 3:** Comparison of genomic coverage.

Platform	Allele frequency			
	# of samples	Total (MAF ≥ 0.01)	Common (MAF ≥ 0.05)	Less common. (0.01 ≤ MAF < 0.05)
**KoreanChip**	**6**,**949**	**89**.**86**	**95**.**38**	**73**.**65**
Affymetrix 5.0	6,949	76.25	84.78	51.23
Affymetrix 6.0	3,695	83.93	91.67	61.23
Illumina Omni 1 M	3,666	86.97	94.10	66.01
**KoreanChip**	**96**	**88**.**37**	**95**.**24**	**68**.**22**
Axiom Biobank	96	81.94	91.56	53.74
UK Biobank	96	85.21	94.05	59.30
Axiom PMRA	96	87.09	94.48	65.42
Illumina GSA	96	84.38	92.27	61.24

All genomic coverage information was calculated using imputed data.

Genomic coverages of KoreanChip are shown in bold-face.

Affymetrix 5.0, Affymetrix Genome-wide Human SNP array 5.0; AFFY6.0, Affymetrix Genome-wide Human SNP array 6.0; ILLU Omni 1 M, Illumina HumanOmni1-Quad BeadChip; Illumina Exome array, Illumina HumanExome BeadChip; Illumina GSA, Illumina Global Screening Array; Axiom PMRA, Axiom Precision Medicine Research Array.

### GWAS for blood biochemical traits

We performed preliminary GWAS using 6,945 samples of KoreanChip data for various biochemical traits including lipids (high-density lipoprotein cholesterol (HDLc), low-density lipoprotein cholesterol (LDLc), total cholesterol (TCHL), and triglyceride (TG)), and liver enzymes (aspartate aminotransferase (AST) and alanine aminotransferase (ALT)). First, we compared the association results with those of 6,945 identical samples genotyped with Affymetrix 5.0. Supplementary Fig. S5 shows side-by-side Manhattan plots of each trait of the two GWASs. The pattern of associations in the Manhattan plots was very similar to that of previous studies. We compared P-values of previously reported biochemical trait-associated SNPs between KoreanChip and Affymetrix 5.0^17^. Supplementary Table S4 shows that the association results from the two different platforms were similar. We also compared the imputation performance of variants with P ≤ 10^−6^ in the results of either association (Supplementary Fig. S4). Overall, KoreanChip showed a highly enhanced imputation quality compared with Affymetrix 5.0.

GWAS using KoreanChip identified 28 loci with P < 5 × 10^−8^ and MAF >0.01. All of these loci were previously known (Supplementary Table S5). Among them, recently identified East Asian population-specific missense variants such as rs151193009 on *PCSK9*, rs13306194 on *APOB*, rs2075291 on *APOA5*, and rs2303790 on *CETP*^30^ were recapitulated in our study. Taking advantage of functional variants included in the KoreanChip, we further investigated nonsynonymous variants responsible for the variation of traits. Nonsynonymous variants were selected if P was < 1 × 10^−4^ and MAF was >0 within any locus containing a lead signal with P < 5 × 10^−7^ and one or more supporting signals with P < 1 × 10^−4^. As a result, 18 missense variants were discovered and were subjected to replication analysis by TaqMan SNP genotyping assay using 6,000 independent cohort samples. Among them, 3 rare variants were not successfully genotyped, and 6 rare variants did not satisfy statistical significance or had a different direction of effect; 9 variants were successfully replicated (P < 0.05, Table 4). All 9 variants were newly discovered in this study. Seven new associated variants were located within known loci. Among them, two variants that were Asian-specific with MAF >0.1% in East Asians were monomorphic or extremely rare in populations with different ancestries. Moreover, a negative correlation of two novel missense variants, rs200088103 (p.Arg133Trp) and rs748547625, (p.Arg143Cys), with rare frequency in a novel locus (*GPT*) was identified. Additive genetic effects of two variants showed a reduction in ALT level of 7.0% (1.982 IU/L) and 5.9% (1.658 IU/L) of the mean value, respectively. *GPT* encodes cytosolic alanine aminotransaminase 1 (ALT1), which is also known as glutamate-pyruvate transaminase 1. This enzyme plays a key role in the intermediary metabolism of glucose and amino acids (http://www.genecards.org). Interestingly, despite the close relationship between the ALT trait and *GPT*, to our knowledge, this is the first report demonstrating a genetic link between ALT and functional variants of *GPT*.

**Table 4:** Nine Newly identified missense variants in known or novel loci.

Gene	SNPs	Position	RA/AA	EA/OA	Trait(s)	EAF(%)				Discovery		Replication		Availability in commercial chips
						KOR	gnomAD			(~6,949 samples)		(~6,000 samples)		
							EAS	EUR	AFR	Beta(SE)	P-value	Beta(SE)	P-value	
**5 variants at known loci**														
AC109829.1	rs965813	chr2:27789861	T/A	A/T	TG	33.31	37.00	20.86	21.53	−0.0415(0.0089)	3.27E-06	−0.0483(0.0105)	4.26E-06	ILMN 1 M
C2orf16	rs1919128	chr2:27801759	A/G	G/A	TG	52.87	47.81	27.16	6.61	0.0379(0.0084)	7.20E-06	0.0560(0.0100)	2.36E-08	ABA, PMRA, UKB, ILMN 1 M, ILMN exome
BUD13	rs10488698	chr11:116633947	G/A	A/G	HDL	6.61	7.22	6.06	1.16	0.0330(0.0073)	7.04E-06	0.0229(0.0081)	4.66E-03	ABA, UK Biobank, ILMN 1 M, ILMN exome
C19orf80, DOCK6	rs2278426	chr19:11350488	C/T	T/C	LDL	27.31	25.93	4.42	18.05	−0.0203(0.0056)	3.16E-04	−0.0281(0.0058)	1.57E-06	ABA, PMRA, UKB, ILMN 1 M
					TCHL	27.02				−3.8231(0.6689)	1.14E-08	−3.6170(0.7294)	7.29E-07	
APOE	rs440446	chr19:45409167	C/G	G/C	LDL	37.47	39.62	63.57	85.81	−0.2010(0.0052)	1.23E-04	−0.0210(0.0055)	1.31E-04	UKB
					TG	37.60				0.0314(0.0087)	2.84E-04	0.0263(0.0104)	1.17E-02	
**2 variants at known loci (Asian-specific)**														
APOB	rs13306206	chr2:21242731	G/A	A/G	LDL	0.97	0.26	0	0	0.1509(0.0259)	5.87E-09	0.1117(0.0256)	1.27E-05	ABA, PMRA, ILMN exome
					TCHL	0.94				15.9680(3.1140)	3.01E-07	13.2300(3.2040)	3.69E-05	
ALDH2	rs671	chr12:112241766	G/A	A/G	ALT	15.67	25.65	0.002	0.02	−0.0586(0.0107)	4.98E-08	−0.0481(0.0114)	2.86E-05	ABA, PMRA, UKB, ILMN 1 M, ILMN exome, ILMN GSA
					AST	15.67				−0.0541(0.0075)	5.20E-13	−0.0372(0.0075)	8.14E-07	
**2 novel variants at novel loci (Asian-specific)**														
GPT	rs200088103	chr8:145730416	C/T	T/C	ALT	0.12	0.10	0.004	0	−0.6843(0.1140)	2.02E-09	−0.5574(0.1023)	5.30E-08	ILMN GSA
GPT	rs748547625	chr8:145730446	C/T	T/C	ALT	0.14	0.11	0	0	−0.5058(0.1048)	1.41E-06	−0.4972(0.1024)	1.24E-06	—

Chromosomal position is based on hg19. Traits were transformed to follow a normal distribution by natural log except TCHL.

CHR, chromosome; POS, position; RA, Reference allele; AA, Alternative allele; EA, Effective allele; OA, Other Allele; EAF, Estimated Allele Frequency; GnomAD, the Genome Aggregation Database; KOR, Korean; EAS, East Asian; EUR, European (non-finnish); ABA, Axiom Biobank array; PMRA, Axiom Precision medicine research array; UKB, Axiom UK Biobank array; ILMN 1 M, Illumina HumanOmni1-Quad BeadChip; ILMN Exome, Illumina HumanExome BeadChip; ILMN GSA, Illumina Global Screening Array.

## Discussion

The Korea Biobank Array Project was initiated to identify the contributions of genomic variants to health and diseases frequently identified in Koreans. To this end, we planned to produce genome data optimized for the Korean genomic structure, with the goal of integrating genomic data and comprehensive cohort resources, including human-derived materials and epidemiological data collected from the prospective cohort in the general population.

With careful attention to performance, including accuracy, reliability, and utility, we designed and implemented an axiom platform-based genome-wide genotyping array named KoreanChip. Our primary goals in the design of KoreanChip were as follows: (1) minimize the number of markers filtered by QC because of ethnic difference, resulting in maximum utilization of KoreanChip; (2) include the highest possible amount of potentially damaging variants observed in Koreans that can directly affect coding sequence; (3) achieve higher imputation-based genomic coverage of common and low-frequency variants; (4) ensure cost-effectiveness to provide more genomic information on the same budget to facilitate genome–phenome studies.

The results of the performance test confirmed that KoreanChip is a reliable genotyping platform and comparable to validated, widely used genotyping platforms. The average reproducibility over 35 duplicates was 99.8%. When comparing the accuracy using the same markers in existing platforms, directly genotyped variants showed >99.8% and >99.7% accuracy for common and rare variants, respectively (Supplementary Table S1).

Over the past decade, Korean genome studies have employed commercial SNP arrays such as Affymetrix 5.0, Affymetrix 6.0, and Illumina exome arrays designed for populations of European ancestry or multiethnic populations. However, only approximately ~70% of the markers were available for further association study after the QC process^16,17^. With respect to marker availability, KoreanChip achieved higher availability than existing commercial arrays for Korean genome study. Indeed, the percentage of quality-controlled markers in KoreanChip was ~97% (Supplementary Table S6), resulting in maximally available markers by minimizing the markers excluded because of ethnic difference.

Rare variants are not well imputed, and aggregate tests are sensitive to genotype errors^28^. Therefore, direct detection of rare variants is likely to be required for significant progress in detection of trait variation caused by rare variants^28^. With respect to functional and rare variant detection, the most notable feature of KoreanChip is its content, which includes the highest number of nonsynonymous SNPs in East Asians compared with other commercial arrays (Table 2). KoreanChip newly introduced approximately 208 K functional variants including nonsynonymous SNPs in Koreans. Notably, ≥89 K SNPs were estimated to be damaging in East Asian populations (1000 Genomes Project). These variants are likely to directly detect disease/trait-associated rare variants. Moreover, directly genotyped rare variants would be helpful to enhance imputation performance and genomic coverage when KoreanChip is used for generating an imputation panel with a combination of existing SNP arrays and sequencing data^31,32^.

In this study, two missense rare variants at GPT were newly identified. Interestingly, a genetic link between ALT and functional variants of *GPT* has not been previously reported by GWAS of liver enzymes. Our finding suggests that our strategy for the selection of directly genotyped rare/functional variants would be effective to identify functional variants observed at rare and low frequencies in East Asian, including Korean, populations. In fact, a database recently developed by the Genome Aggregation Database (gnomAD) consortium (http://gnomad.broadinstitute.org/) showed that these two novel loci tended to have rare allele frequencies in East Asians (MAF ≥ 0.1%) but were extremely rare (≤0.005%) or monomorphic in other ethnic groups (Table 4). Our association results also showed MAFs (rs200088103, MAF = 0.12% and rs748547625, MAF = 0.14%) similar to those of gnomAD (Table 4). Recently, the exome-wide association study using WES by DiscovEHR reported 7 genetic variants on *GPT* associated with ALT. In contrast, in our GWAS results, 6 variants were monomorphic, and one variant was extremely rare (≤0.005%) in East Asians. Interestingly, no common variant has been found on *GPT* to be associated with ALT in GWAS to date. Furthermore, studies using both European (DiscovEHR) and East Asian (KoreanChip) descents did not find any such common variant. Because rare-frequency variants tend to be population-specific, these variants are not listed in existing commercial arrays. Therefore, our approach represents a promising method to determine new disease-associated rare/functional variants that were not identified previously with existing commercial arrays. Moreover, our approach is more cost-effective than WGS or WES-based approaches.

With regard to another missense variant of *ALDH2*, rs671, many studies have demonstrated the association of this variant with metabolic diseases/traits such as T2D, alcohol consumption, metabolic syndrome, and coronary heart disease^33^. More recently, a study from Biobank Japan (BBJ) demonstrated a pleiotropic association of *ALDH2* with AST and ALT, but rs671 did not demonstrate such an association^34^. Furthermore, although several existing commercial and next-generation arrays contain rs671, no relationship between this missense variant and ALT level has been reported^17,35^.

Even if ethnic differences are not taken into consideration, this new association reveals the current limitations of GWAS due to the lack of non-European genomic data, suggesting that rich resources of genomic data from diverse populations, including those of East Asia, are needed to provide further insight into complex diseases.

KoreanChip was used to conduct genomic studies using detailed phenotypes and human-derived resources collected from government-funded biobanks such as Axiom UK Biobank, Million Veteran Program, Kadoorie biobank, and BBJ. Currently, the Korea biobank array has provided data to ≥100 Korean research groups performing disease-related genome research and is anticipated to have ≥210,000 genomic datasets by 2019. Even though more genome data from diverse populations are essential to elucidate the potential impact of ethnicity, non-European participants represent only 19% of individuals studied in GWAS^36^. Therefore, together with BBJ, KoreanChip will be an important resource to address this lack of East Asian genomic information, as one of the largest Asian genome resources originating from a single ancestry.

In conclusion, we designed and implemented KoreanChip with the most current content optimized for the Korean population and highest coverage and accuracy for GWAS. The performance of KoreanChip was evaluated through several reliability tests. The large-scale KoreanChip data with many features may represent a valuable resource to fill the lack of diversity of genomic data. Furthermore, our findings in the preliminary GWAS suggested that abundant functional variants, which were directly genotyped, can provide insight into novel variants contributing to population-specific diseases as well as potential implications for clinical practice.

## Methods

### The Korea Biobank Array Project

The Korea Biobank Array Project was launched in 2014 by the Korea National Institute of Health. It aims to produce KoreanChip data of all participant samples collected by KoGES and stored in the National Biobank of Korea (NBK). In population-based cohorts of KoGES, approximately 210,000 participants were independently recruited from three different cohorts^21^. Supplementary Table S7 provides detailed information regarding KoGES. All participants aged 40–69 years were examined through epidemiological surveys, physical examinations, and laboratory tests. The study design and samples were approved by an institutional review board at the Korean National Institute of Health, Republic of Korea. In the current study, we evaluated the KoreanChip design process and its characteristics using approximately 7,000 subjects. Supplementary Table S8 shows demographic characteristics of the study population.

Moreover, over the last decade, ≥25,000 samples as a part of KoGES were genotyped^16,17,26^, and GWAS have been conducted to identify complex trait-associated variants^16,17,19,20^. These data were also used for evaluation of KoreanChip performance.

### Array design and marker selection

Initially, we obtained information regarding MAF, chromosomal position, and alleles from approximately 22,000,000 variants of 2,576 sequenced Korean samples. These data consisted of 397 WGS from the Korean Reference Genome^37^ and 2,179 WES encompassing 1,087 Korean samples from the T2D-GENES consortium^1^, 200 Korean samples from the Ansung and Ansan study^38^, 200 samples from a cardiovascular disease sequencing study^39^, and 692 samples from the Korean Children and Adolescents Obesity Cohort study^40^.

To select markers optimized for the Korean genome, we especially focused on maximizing genome-wide tagging markers that boosted imputation performance and potentially functional markers influencing disease risk. Supplementary Fig. S1 shows the schematic representation of the KoreanChip design process. To screen validated SNPs among 22 M variants, we referenced the Affymetrix Axiom Genomic Database (AxiomGD), which contains over 26 M validated high-performance markers (https://tools.thermofisher.com/content/sfs/brochures/axiom_biobank_solution_brochure.pdf). Subsequently, *de novo* variants not in the AxiomGD were experimentally validated using a customized Axiom myDesign genotyping array (Life Technologies, Carlsbad, CA, USA), a screening array with 1.6 M selected variants of interest.

We used dosage r^2^ and F_st_ for selecting variants with accurate genotype calling^41^. Generally, dosage r^2^ is squared Pearson correlation coefficient between imputed dosages (continuous variable rating 0~2) and true genotypes (0, 1, and 2). This refers to the magnitude the of linear relationship between two variables. Therefore, dosage r^2^ value 1 indicates that there is a perfect linear relationship between two variables. Using genotypes of 384 samples from both 1.6 M screening array and WGS data, we measured dosage r^2^ for each variant by calculating squared Pearson correlation coefficient between genotypes from the screening array and WGS data. Moreover, F_st_ was calculated using genotypes from the screening array and WGS data. Variants were considered validated if dosage r^2^ was ≥0.7, F_st_ was ≤0.025, and if the genotype cluster was acceptable based on SNPolisher analysis (https://tools.thermofisher.com/content/sfs/manuals/SNPolisher_User_Guide.pdf). Only validated markers were used for further marker selection. Considering the 7.7 M variants with MAF ≥ 1% in 2,579 Korean sequencing data (397 WGS and 2,179 WES), the Affymetrix imputation-aware selection algorithm was used to select genome-wide tagging markers providing genome-wide coverage for autosomal chromosomes^22^. Content modules were selected based on the following criteria: (1) nonsynonymous SNPs with MAF ≥ 0.1%; (2) nonsynonymous SNPs with MAF < 0.1% predicted to be damaging based on the annotations from dbNSFP database v2.4^23^, where a variant was selected if a damaging effect was predicted by SIFT^42^ or Polyphen 2^43^ (HDIV and HVAR); and (3) markers of specific interest such as the available modules in AxiomGD of eQTL, human leukocyte antigen (HLA), and NHGRI GWAS catalog variants^24^.

To maximize the number of variants on the chip and prevent ambiguous strand problems, variants with A/T or C/G alleles were not included except in the case of indispensable tagging variants, nonsynonymous SNPs with MAF ≥ 0.1%, and previously known markers of specific interest. The variants with A/T or C/G alleles required four features, compared with the one or two features required for variants with other allele combinations, depending on quality metrics related to genotype calling^10^.

### Genome data for performance tests

**Figure 1:**
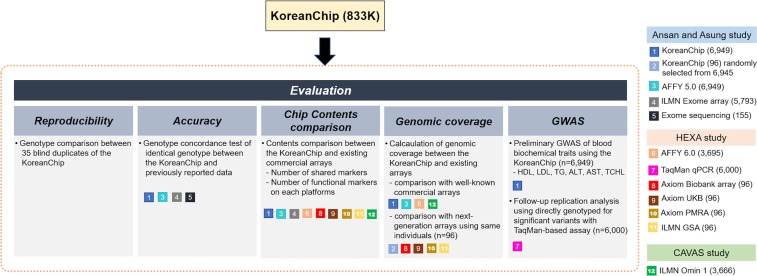
Overall scheme of KoreanChip evaluation process. Evaluation of KoreanChip consisted of 5 steps: reproducibility, accuracy, content comparison, genomic coverage, and application to GWAS. All subjects were selected from three independent cohorts: Ansan and Ansung study, Health EXAminee (HEXA), and CArdioVascular disease Association Study (CAVAS). Datasets for the test are shown in colored squares with number. The number in parentheses indicates the number of subjects genotyped with the corresponding platform.

Ten different platforms were used to conduct performance evaluation in terms of genotyping accuracy, genomic coverage, and utility in genome-wide association analysis. Figure 1 shows the overall evaluation process of the KoreanChip performance.

Identical samples from the Ansan and Ansung study were genotyped with KoreanChip (6,949 subjects), Affymetrix 5.0 (6,949 subjects), and Illumina exome (5,793 subjects) arrays (Fig. 1 and Supplementary Table S6). For genomic coverage and chip content comparison, 96 subjects randomly selected from the Ansan and Ansung study were genotyped with KoreanChip. Furthermore, 96 identical samples from the Health Examinee (HEXA) study were genotyped with the Axiom Biobank array, Axiom UK Biobank array, Axiom PMRA, and Illumina Global Screening Array (GSA). An additional 3,695 and 3,666 subjects from HEXA and a cardiovascular disease association study (CAVAS) were genotyped with Affymetrix 6.0 and Illumina Omni 1 M, respectively. The datasets used in this study are summarized in Supplementary Table S6.

Genotypes of all samples were called in a single batch except for Affymetrix 5.0 genomic data, of which we used previously called genotypes from Cho *et al*.^16^. For Axiom platforms of Affymetrix, genotypes were called using BRLMM-P algorithm of apt-genotype-axiom (http://www.affymetrix.com/support/developer/powertools/changelog/apt-genotype-axiom.html). Genotypes of Illumina arrays were called using genotype calling module of GenomeStudio software (https://www.illumina.com/techniques/microarrays/array-data-analysis-experimental-design/genomestudio.html). Genotyping with Affymetrix 5.0, Affymetrix 6.0, Illumina exome array, and Illumina Omni 1 M and quality control procedures have been described in detail previously^16,17,26,44^. Briefly, only genotyped samples that passed the following exclusion criteria were used for further analysis: low call rate (<96–99%), excessive heterozygosity, cryptic first-degree relatives, and sex inconsistency. SNPs were removed based on the following criteria: Hardy–Weinberg equilibrium P < 10^−6^, genotype call rates < 95%, and MAF < 1% (only applied to Affymetrix 5.0, 6.0, and Illumina Omni 1 M) (see Supplementary Table S6).

Among the 6,949 genotyped samples of the Ansan and Ansung study, 155 were subjected to WES using Agilent Human Exon v2 capture (Fig. 1 and Supplementary Table S6). Read-pairs were aligned to the reference genome hg19, and the subsequent variant calling was performed using the Genome Analysis Toolkit v2 (https://software.broadinstitute.org/gatk/). The mean mapped read depth was approximately 60×^38^.

### Genotype imputation

Quality-controlled data were phased using ShapeIT v2^45^. IMPUTE v2^46^ was used for imputation analysis of phased genotype data with 1000 Genomes Phase 3 data as a reference panel. After imputation, imputed variants with imputation quality score < 0.4 or MAF < 1% were excluded from further analysis.

### Content comparison

#### Shared content with existing arrays

All markers of autosomal regions of seven genotyping arrays including KoreanChip were compared. Array SNP information based on reference genome hg19 was retrieved from related annotation files. Shared contents, i.e., overlapping markers between two different genotyping arrays, were selected if variants with exactly identical chromosomal positions and alleles existed in both arrays.

#### Nonsynonymous/rare variant markers

Rare variants have gathered much attention as an alternative source of missing heritability^47^. However, widely used genotyping arrays have been designed mainly for common variants. The imputation approach enabled us to study rare variants, but imputed rare variants have shown low accuracy^31,48^. Therefore, an exome array was designed to contain exonic rare variants for exome-wide association study^12^. More advanced strategies have been adopted in the design of next-generation genotyping arrays; for example, genome-wide tag SNPs and selected rare variants were included in a single array, i.e., the Axiom UK Biobank array^10^. We compared the content of nonsynonymous SNPs, most of which were rare, among KoreanChip and eight other commercial arrays. All SNPs of each array were annotated using SNPEff v4.1d^49^ and dbNSFP v2.7^23^.

#### Nonsynonymous markers observed in East Asians

The low amount of usable content was mainly due to the population specificity of rare variants^50,51^. Because most sequencing data used for the design of the exome array were from Europeans, the majority of the variants on the exome array were monomorphic in Koreans. For example, previously, we analyzed 14,056 samples with the Illumina exome array^26^. In detail, relatives defined based on identity-by-descent and samples with sex inconstancies were excluded. Furthermore, samples with low call rate (<99%) were thrown out. Next, variants with completely missing data or low call rate (genotype call rate <95%) were removed. Moreover, additional variants were excluded based on deviation from Hardy-Weinberg Equilibrium (HWE), a below-threshold *P* value (HWE *P* < 10^−6^), and minor allele count (MAC) < 2. Duplicated variants were removed. As a result, only 77,204 (about 31.8%) of 242,901 variants remained after quality control (Supplementary Table S6).

To assess possible usable nonsynonymous SNPs in East Asians, we examined the number of nonsynonymous variants (MAF >0) in 504 East Asians of the 1000 Genomes Project Phase 3 across KoreanChip and other commercial arrays.

### Performance measures

#### Reproducibility and accuracy

Reproducibility, i.e., concordance rate based on blind duplicate comparisons, was calculated by blindly genotyping 35 samples with KoreanChip. Accuracy was assessed by calculating genotype concordance between KoreanChip and other platforms. Genotype concordance was calculated using vcftools v0.1.1.12b^52^.

#### Genomic coverage

Genomic coverage represents the total proportion of genomic variation captured by an array, either by direct observation or through indirect means such as linkage disequilibrium (LD) or imputation^28^. Current GWAS have increasingly used genotype imputation methods to improve the coverage of variants not directly measured by microarrays using publicly available reference panels^53,54^. Therefore, improved genomic coverage is an important factor for enhancing association mapping performance.

Previously, Nelson *et al*. reported imputation-based genomic coverage of widely used genotyping chips^28^. In the present study, imputation-based genomic coverage was calculated as the number of imputed variants with imputation quality score ≥ 0.8 divided by the total number of common variants (MAF ≥ 1%) in East Asians of the 1000 Genome Project Phase 3. To draw the plot, the mean value of genomic coverage by 5 Mb genomic window size was calculated.

### Application to GWAS

#### Phenotypes

For the performance test of GWAS, we used the same parameters as those of our previous GWAS^17^. In detail, parameters were measured for plasma lipids, i.e., HDLc, LDLc, TCHL, and triglyceride TG, and liver enzymes, i.e., AST and ALT. Biochemical measurements were obtained in the morning before the first meal of the day. Friedewald’s formula was used for calculation of the LDLc concentration. Missing values for individuals with TG >400 mg/dL were assigned to NA. All measurements were transformed with natural log to approximate a normal distribution, except TCHL. Individuals receiving lipid-lowering therapy were excluded from analysis of LDLc, HDLc, and TG. We excluded any participants taking medication likely to influence liver enzyme traits (AST and ALT). All study participants aged ≥ 40 years provided written informed consent, and approval was obtained from the institutional review board. The basic characteristics used in the association analysis are summarized in Supplementary Table S8.

#### Statistical analysis

Association analysis, assuming an additive mode of inheritance, was performed using SNPTEST v2.5.2^53^ adjusting for age, sex, and recruitment area. Top signals were selected using the following criteria: (1) P ≤ 5 × 10^−8^ for MAF ≥ 1%, (2) Nonsynonymous variants with P < 1 × 10^−4^ and MAF >0 for any locus containing a lead signal with P ≤ 5 × 10^−7^ and one or more supporting signals with P ≤ 1 × 10^−4^. Damaging nonsynonymous variants were predicted to be damaging based on annotation by dbNSFP v2.7^23^. We determined the novelty of the identified variants if a known GWAS signal of the same trait existed within a 1 Mb range centred on the variant. Manhattan plots were generated using qqman package R statistics software (https://cran.r-project.org/web/packages/qqman/index.html).

#### Genotyping for de novo replication study

Selected damaging nonsynonymous variants were taken forward to a replication study using the TaqMan SNP genotyping assay (Life Technologies, Foster City, CA, USA). *De novo* genotyping was performed in 6,000 samples from the HEXA study. Associations were tested between variants from *de novo* genotyping and each trait using PLINK v1.9 software (http://zzz.bwh.harvard.edu/plink/).

### Ethics approval

Institutional review board (IRB) approval for human research was obtained, and the principles outlined in the Declaration of Helsinki were followed.

## Supplementary information

Supplementary Materials

## Data Availability

The genome-wide analysis summary statistics for association with blood biochemical traits are available for download from the KoreanChip consortium website, www.koreanchip.org/downloads.
