# Supplementary material for: The Korea Biobank Array: Design and Identification of Coding Variants Associated with Blood Biochemical Traits
Source: Sci Rep. 2019 Feb 4;9:1382. doi: 10.1038/s41598-018-37832-9 (PMC6361960; doi:10.1038/s41598-018-37832-9)
Supplement: Supplementary file 1 — Supplementary Materials [file 41598_2018_37832_MOESM1_ESM.pdf]

Supplementary Materials

The Korea Biobank Array: Design and Identification of Coding Variants Associated with Blood Biochemical Traits

Sanghoon Moon[1‡], Young Jin Kim[1‡], Sohee Han[1], Mi Yeong Hwang[1], Dong Mun Shin[1], Min Young Park[3], Yontao Lu[4], Kyungheon Yoon[1], Hye-Mi Jang[1], Yun Kyoung Kim[1], Tae-Joon Park[1], Dae Sub Song[2], Jae Kyung Park[2], Jong-Eun Lee[3], and Bong-Jo Kim[1*]

1 Division of Genome Research, **2** Division of Epidemiology and Health Index, Center for Genome Science, National Institute of Health, Chungcheongbuk-do, 28159, Republic of Korea

3 DNA link, Incorporated, Seoul, 03759, Republic of Korea

4 Affymetrix, Inc., Santa Clara, USA,

‡ SM and YJK contributed equally to this work.

*Correspondence: Bong-Jo Kim, PhD.

Division of Genome Research, Center for Genome Science, Korea National Institute of Health, Chungcheongbuk-do, 28159, Korea

Phone: +82-43-719-8870; FAX: +82-43-719-8908; E-mail: kbj6181@cdc.go.kr

Fig. S1. Overview of KoreanChip design process.

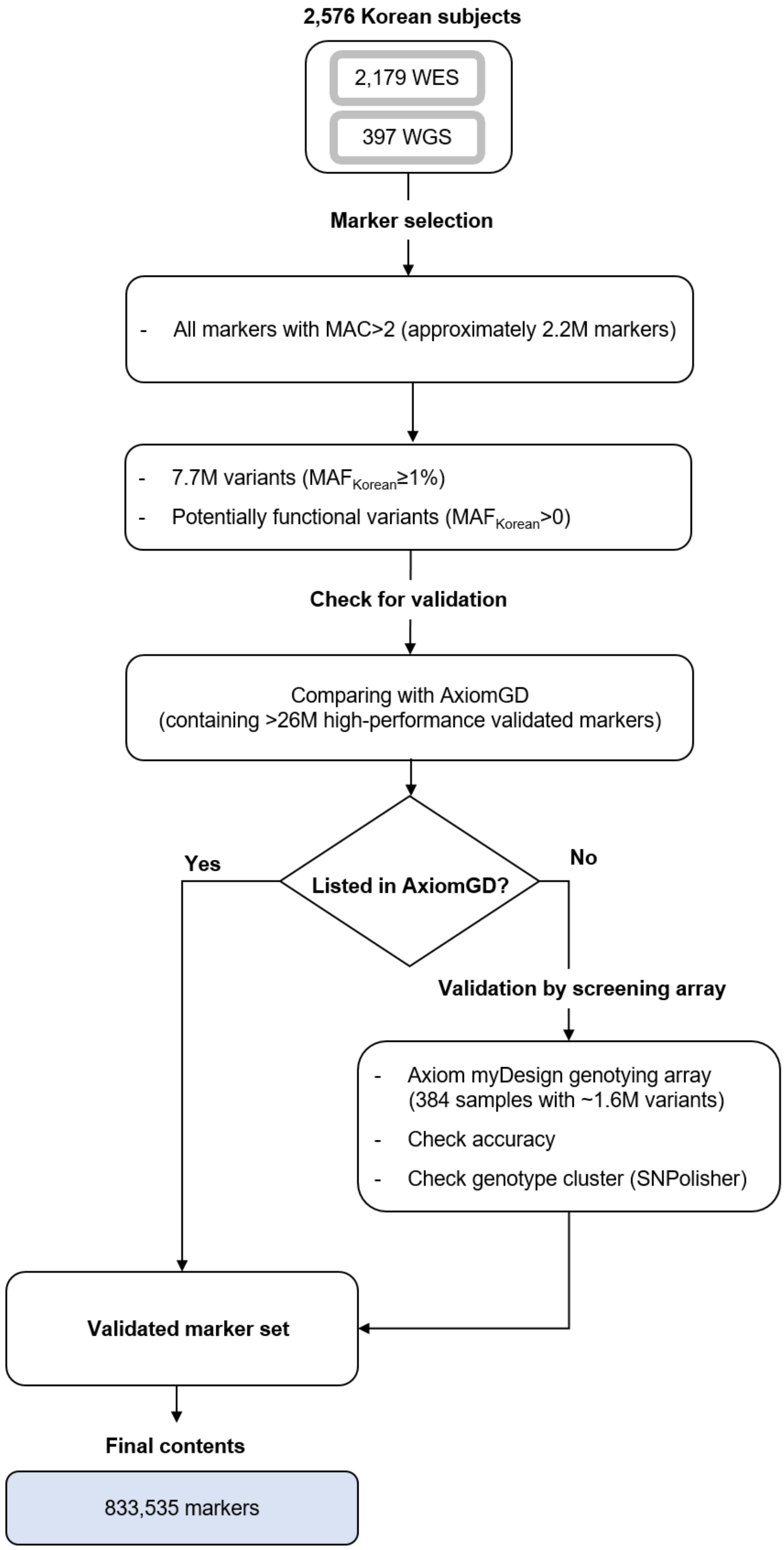

Fig. S2. Genomic coverage of each chromosome (MAF ≥ 5%).

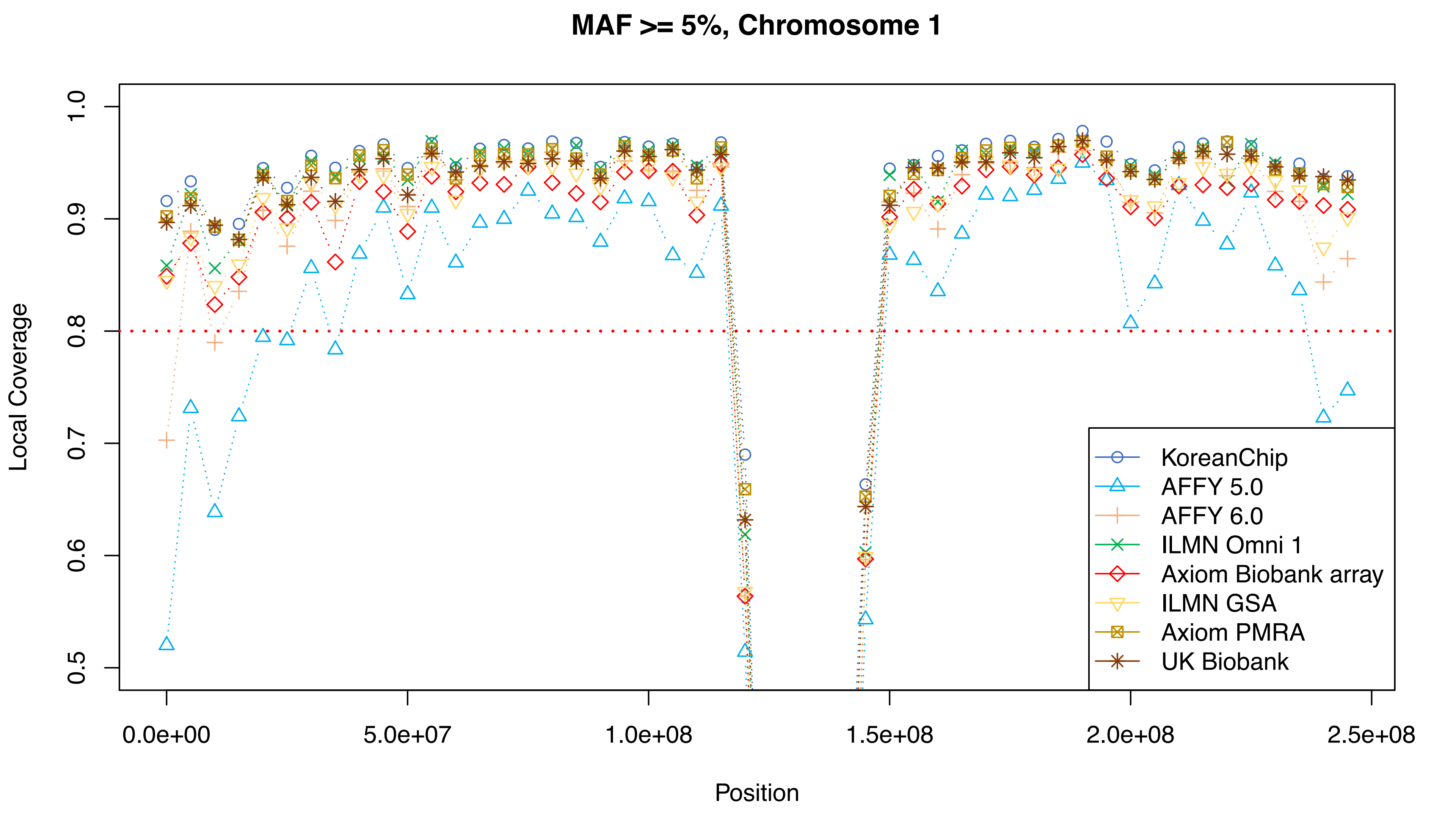

MAF >= 5%, Chromosome 1
Local Coverage
1.0
0.9
0.8
0.7
0.6
0.5
0.0e+00
5.0e+07
1.0e+08
1.5e+08
2.0e+08
2.5e+08
Position
KoreanChip
AFFY 5.0
AFFY 6.0
ILMN Omni 1
Axiom Biobank array
ILMN GSA
Axiom PMRA
UK Biobank

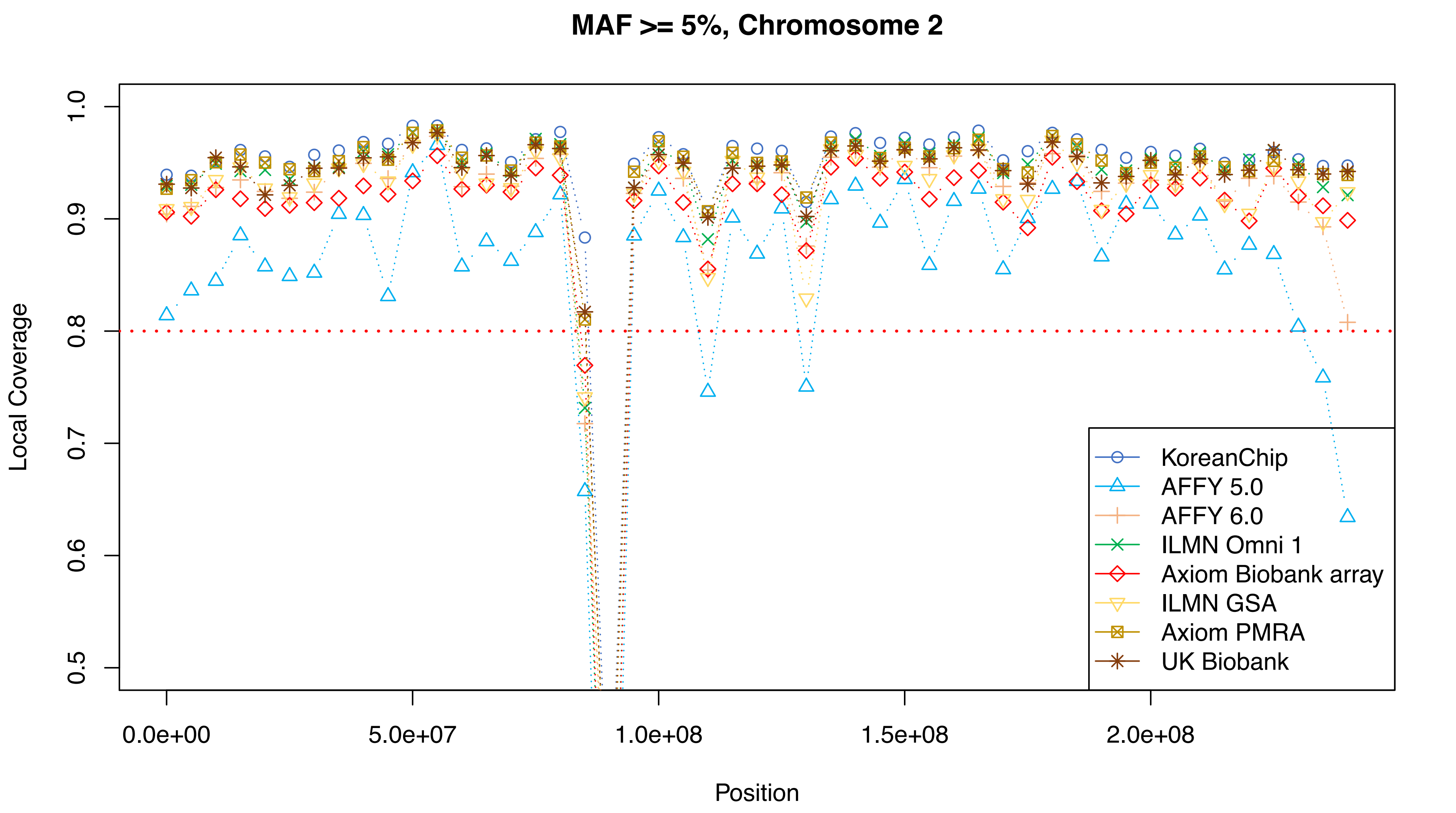
MAF >= 5%, Chromosome 2
Local Coverage
Position
1.0
0.9
0.8
0.7
0.6
0.5
0.0e+00
5.0e+07
1.0e+08
1.5e+08
2.0e+08
KoreanChip
AFFY 5.0
AFFY 6.0
ILMN Omni 1
Axiom Biobank array
ILMN GSA
Axiom PMRA
UK Biobank

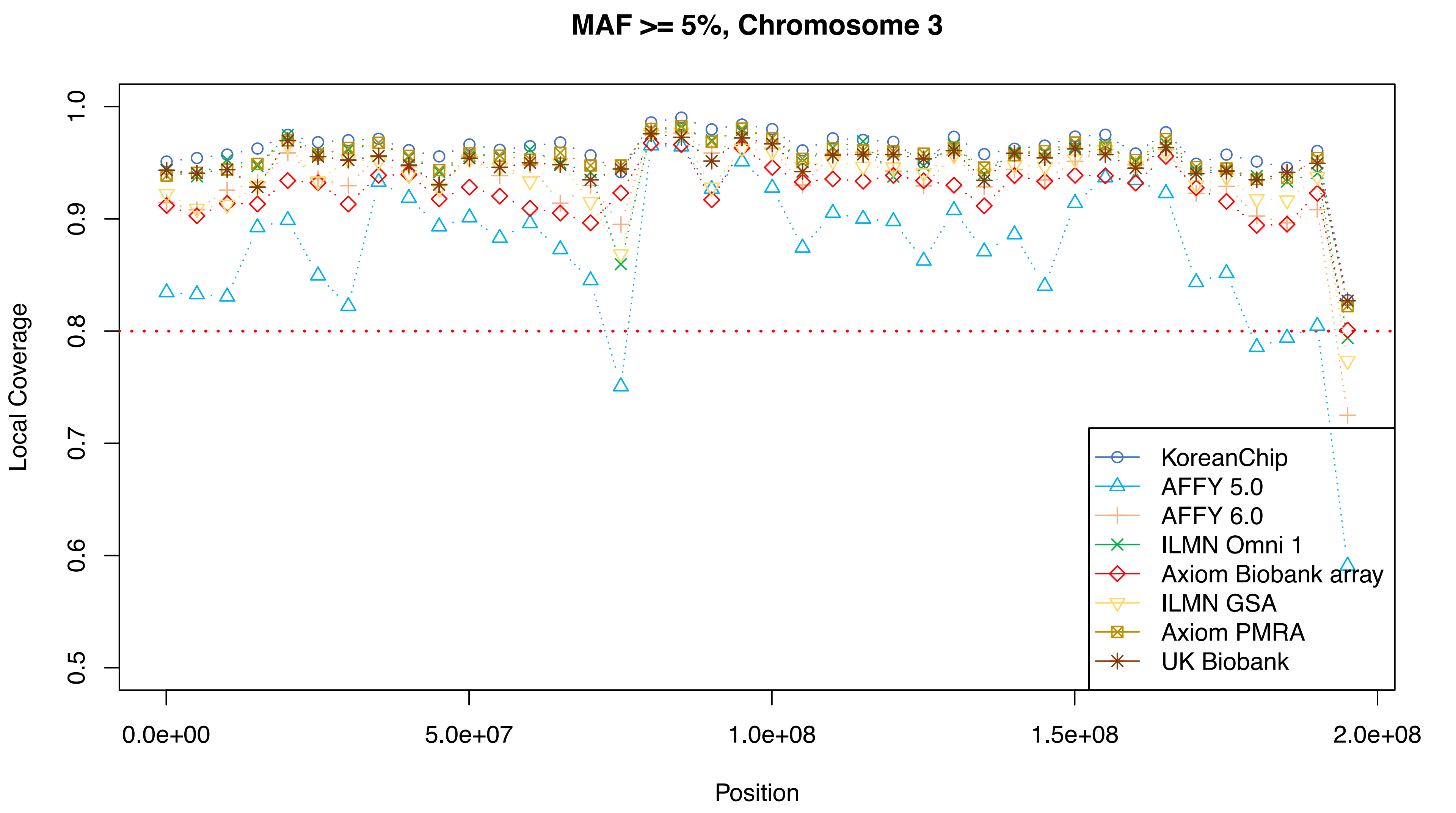

MAF >= 5%, Chromosome 3
Local Coverage
Position
0.5
0.6
0.7
0.8
0.9
1.0
0.0e+00
5.0e+07
1.0e+08
1.5e+08
2.0e+08
KoreanChip
AFFY 5.0
AFFY 6.0
ILMN Omni 1
Axiom Biobank array
ILMN GSA
Axiom PMRA
UK Biobank

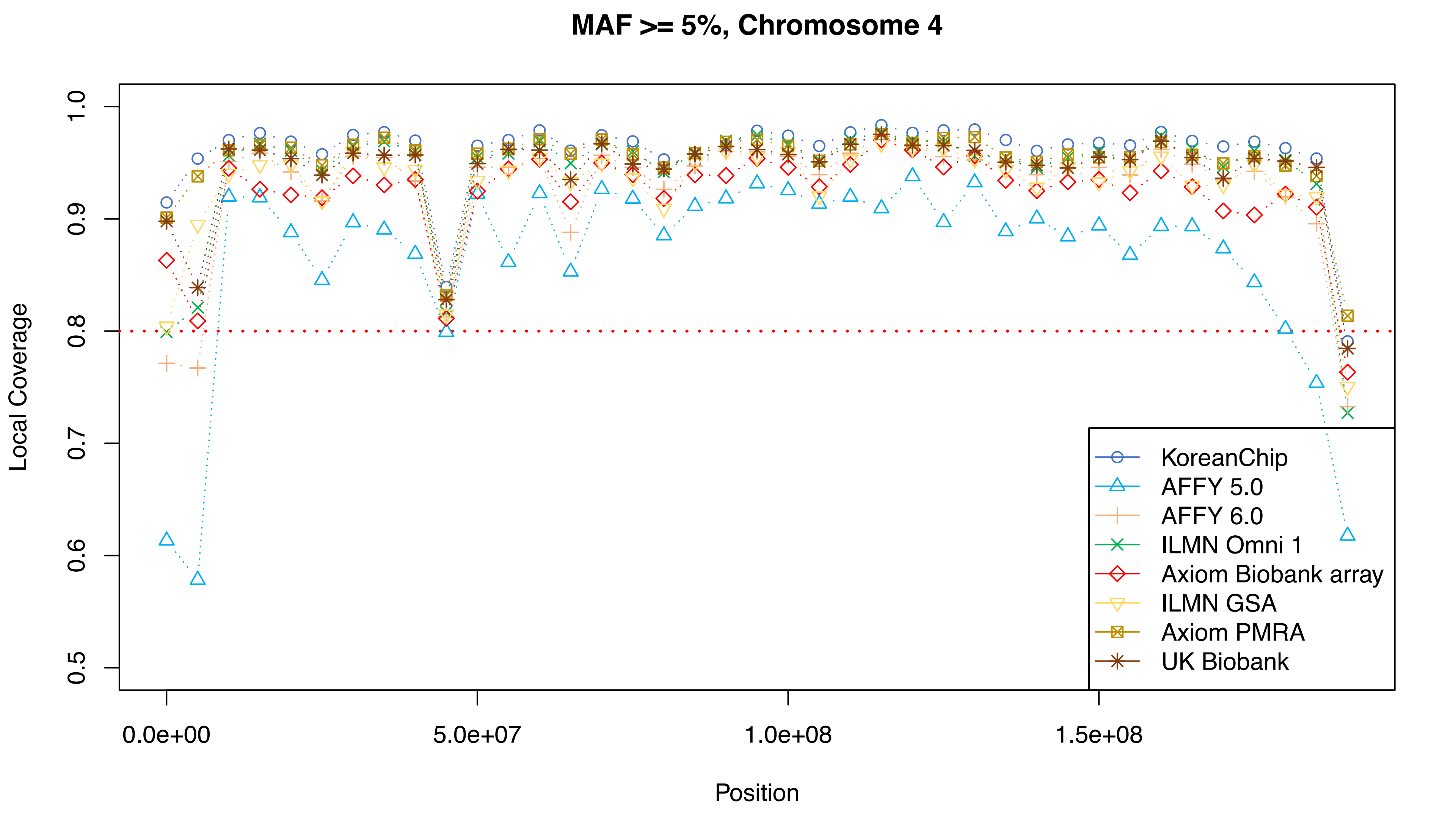
MAF >= 5%, Chromosome 4
Local Coverage
Position
0.5
0.6
0.7
0.8
0.9
1.0
0.0e+00
5.0e+07
1.0e+08
1.5e+08
KoreanChip
AFFY 5.0
AFFY 6.0
ILMN Omni 1
Axiom Biobank array
ILMN GSA
Axiom PMRA
UK Biobank

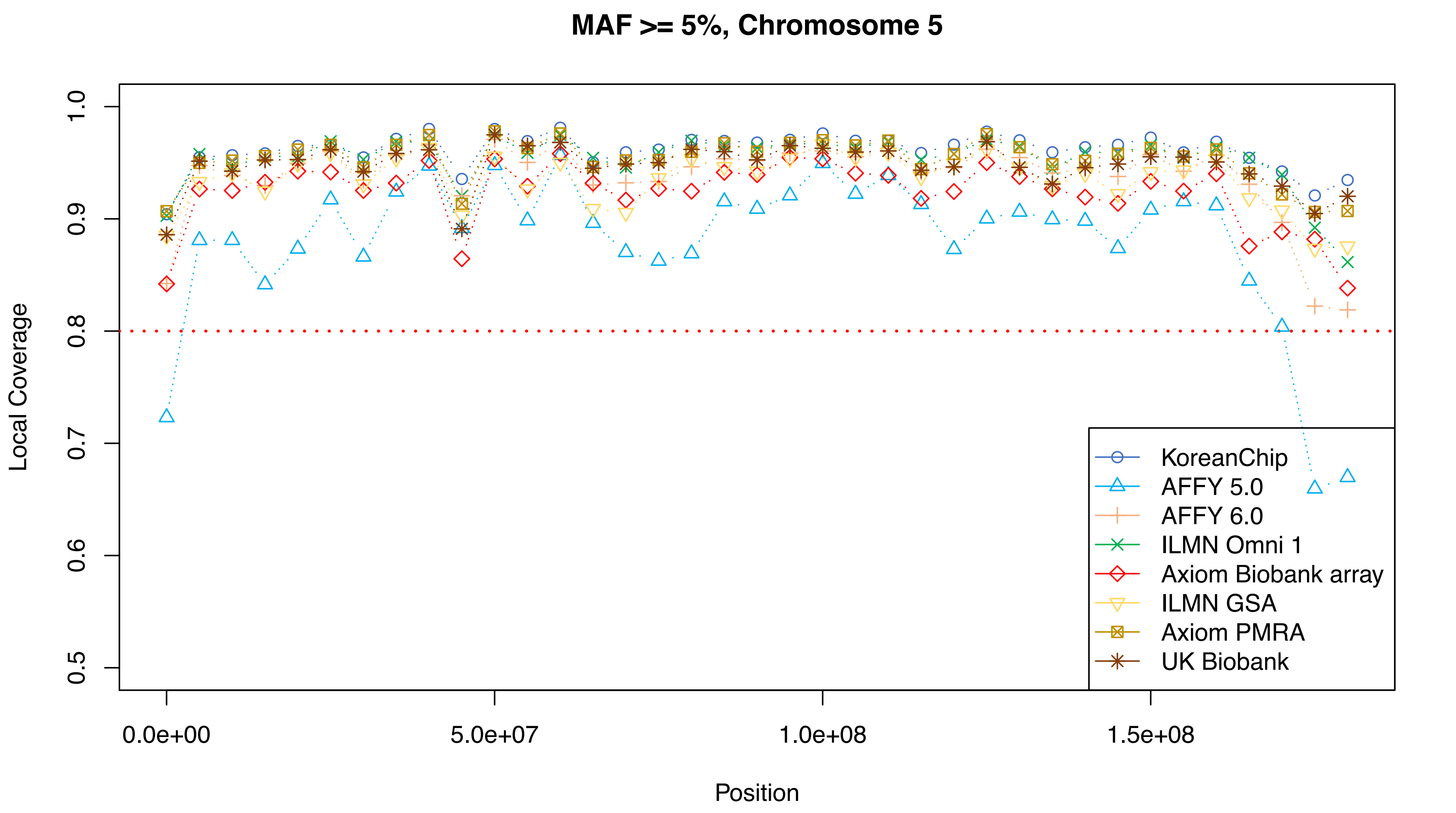

MAF >= 5%, Chromosome 5
Local Coverage
Position
1.0
0.9
0.8
0.7
0.6
0.5
0.0e+00
5.0e+07
1.0e+08
1.5e+08
KoreanChip
AFFY 5.0
AFFY 6.0
ILMN Omni 1
Axiom Biobank array
ILMN GSA
Axiom PMRA
UK Biobank

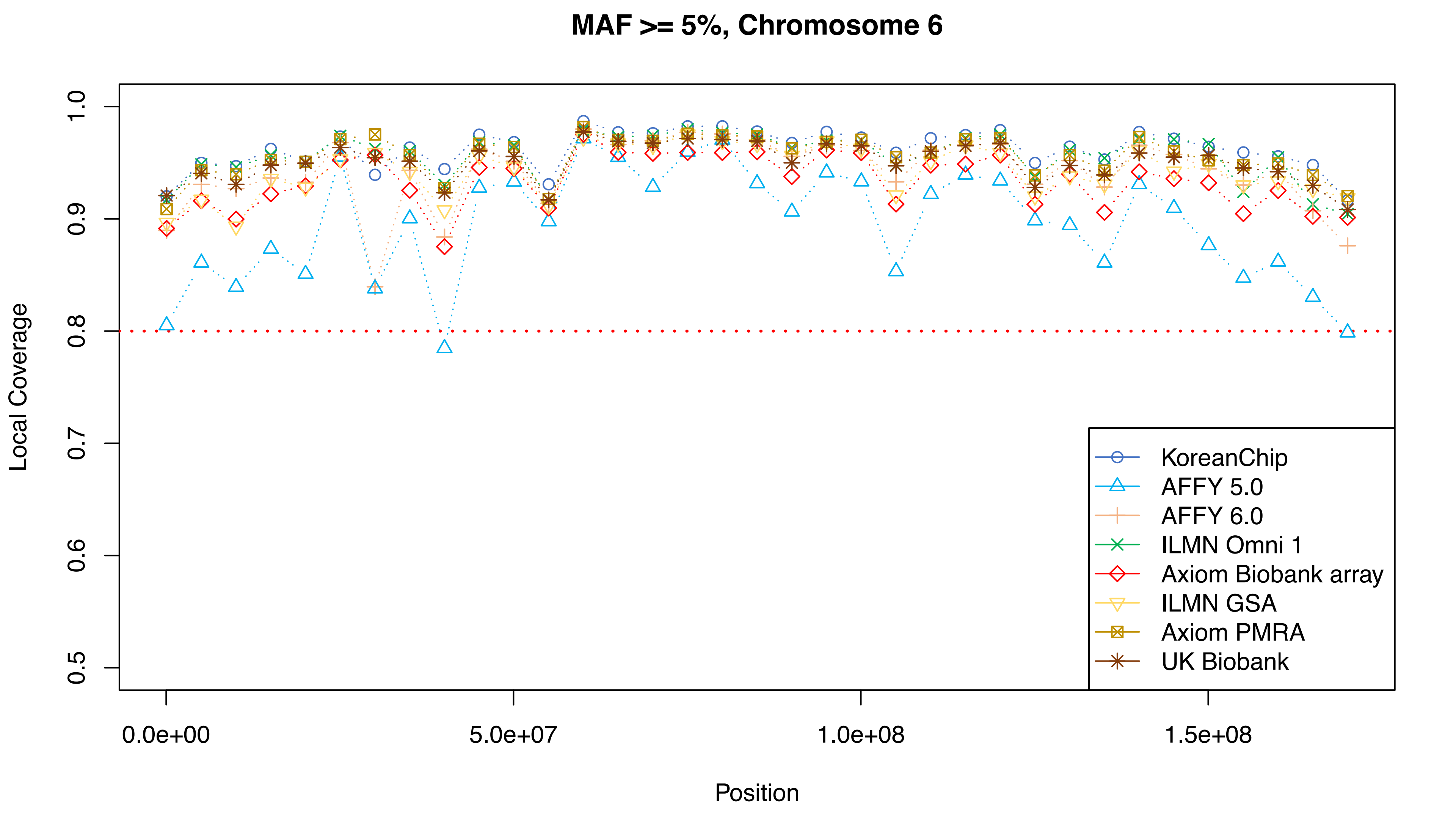
MAF >= 5%, Chromosome 6
Local Coverage
Position
1.0
0.9
0.8
0.7
0.6
0.5
0.0e+00
5.0e+07
1.0e+08
1.5e+08
KoreanChip
AFFY 5.0
AFFY 6.0
ILMN Omni 1
Axiom Biobank array
ILMN GSA
Axiom PMRA
UK Biobank

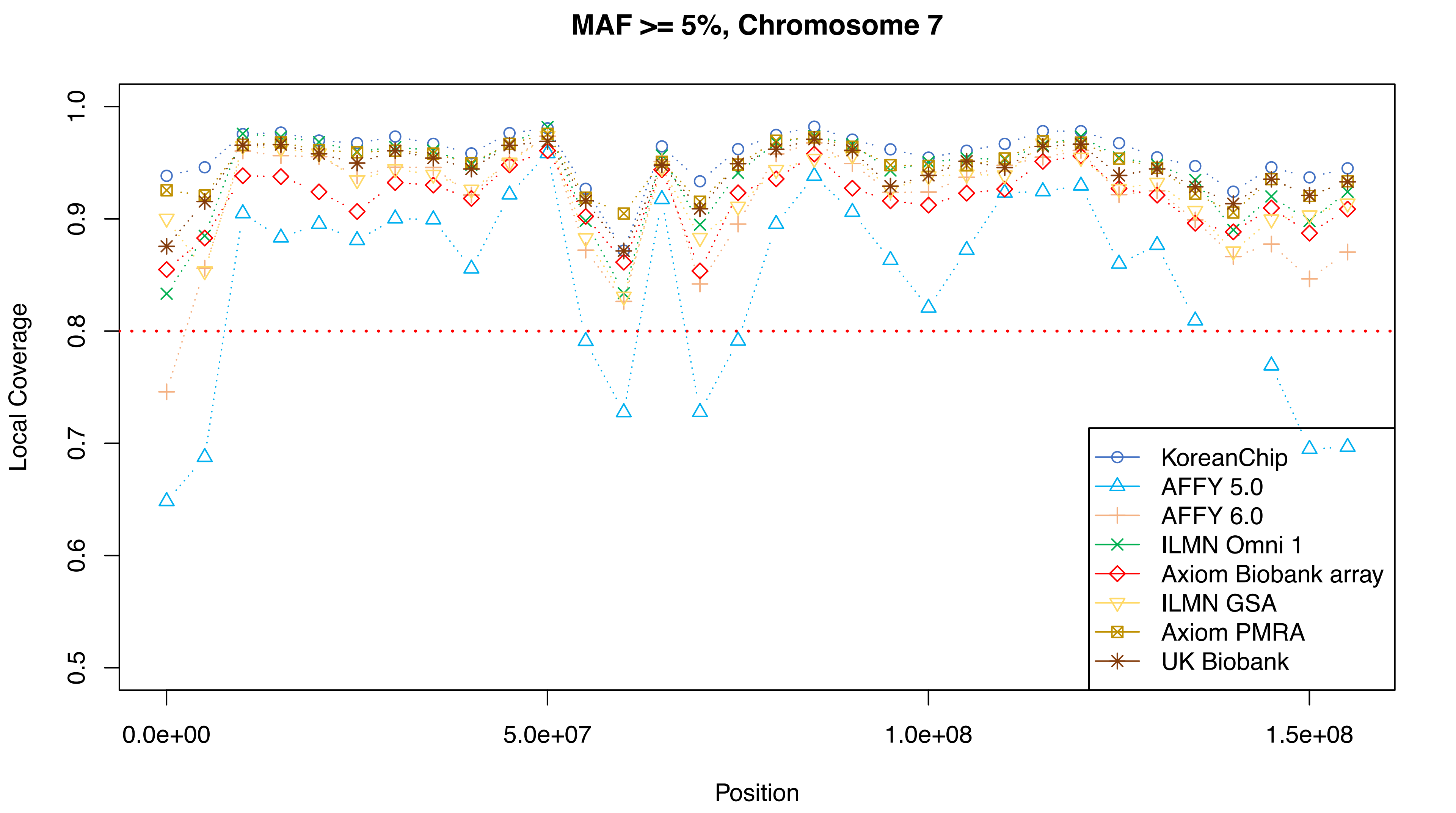
MAF >= 5%, Chromosome 7
Local Coverage
1.0
0.9
0.8
0.7
0.6
0.5
0.0e+00
5.0e+07
1.0e+08
1.5e+08
Position
KoreanChip
AFFY 5.0
AFFY 6.0
ILMN Omni 1
Axiom Biobank array
ILMN GSA
Axiom PMRA
UK Biobank

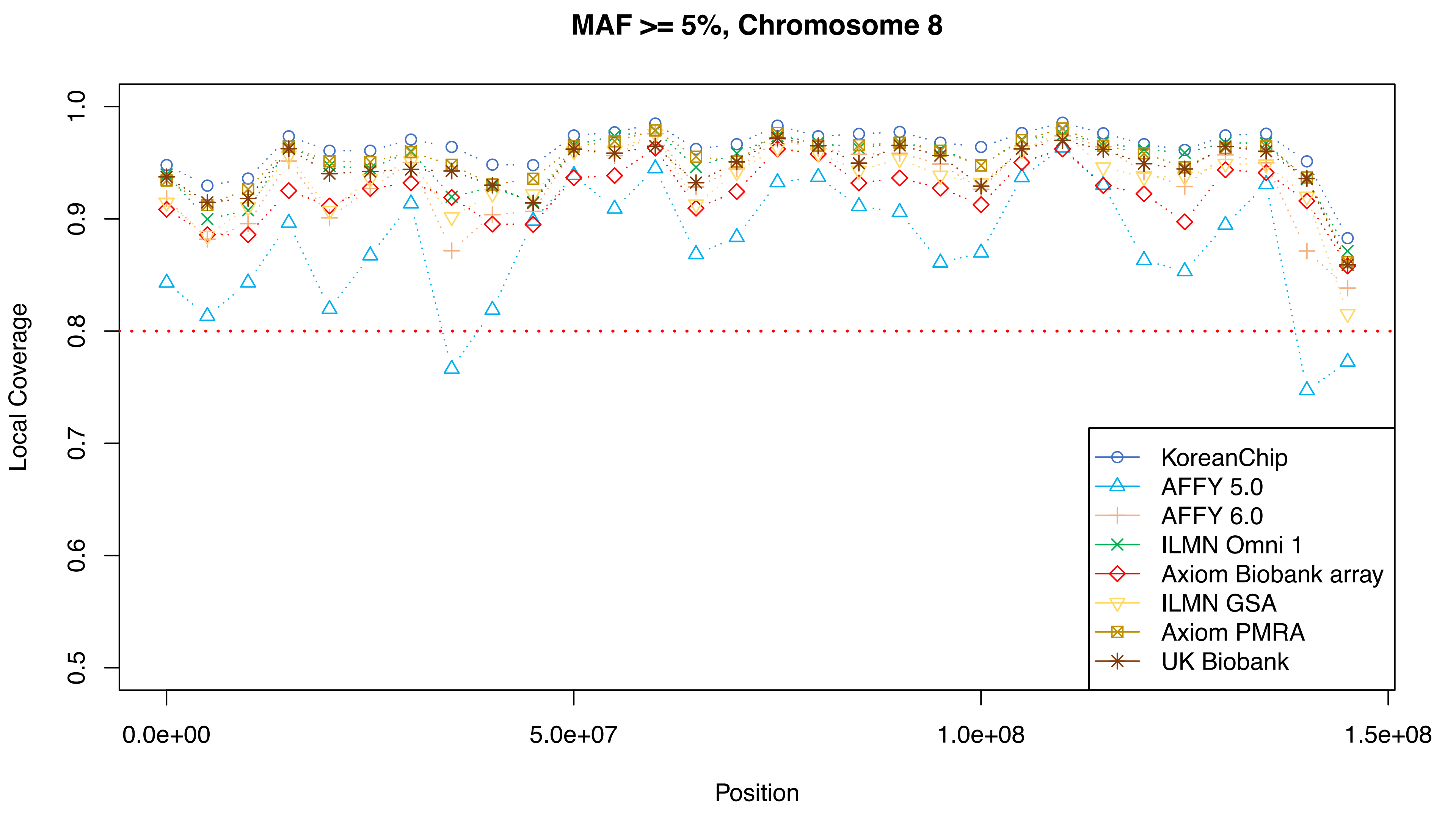

MAF >= 5%, Chromosome 8
Local Coverage
Position
0.5
0.6
0.7
0.8
0.9
1.0
0.0e+00
5.0e+07
1.0e+08
1.5e+08
KoreanChip
AFFY 5.0
AFFY 6.0
ILMN Omni 1
Axiom Biobank array
ILMN GSA
Axiom PMRA
UK Biobank

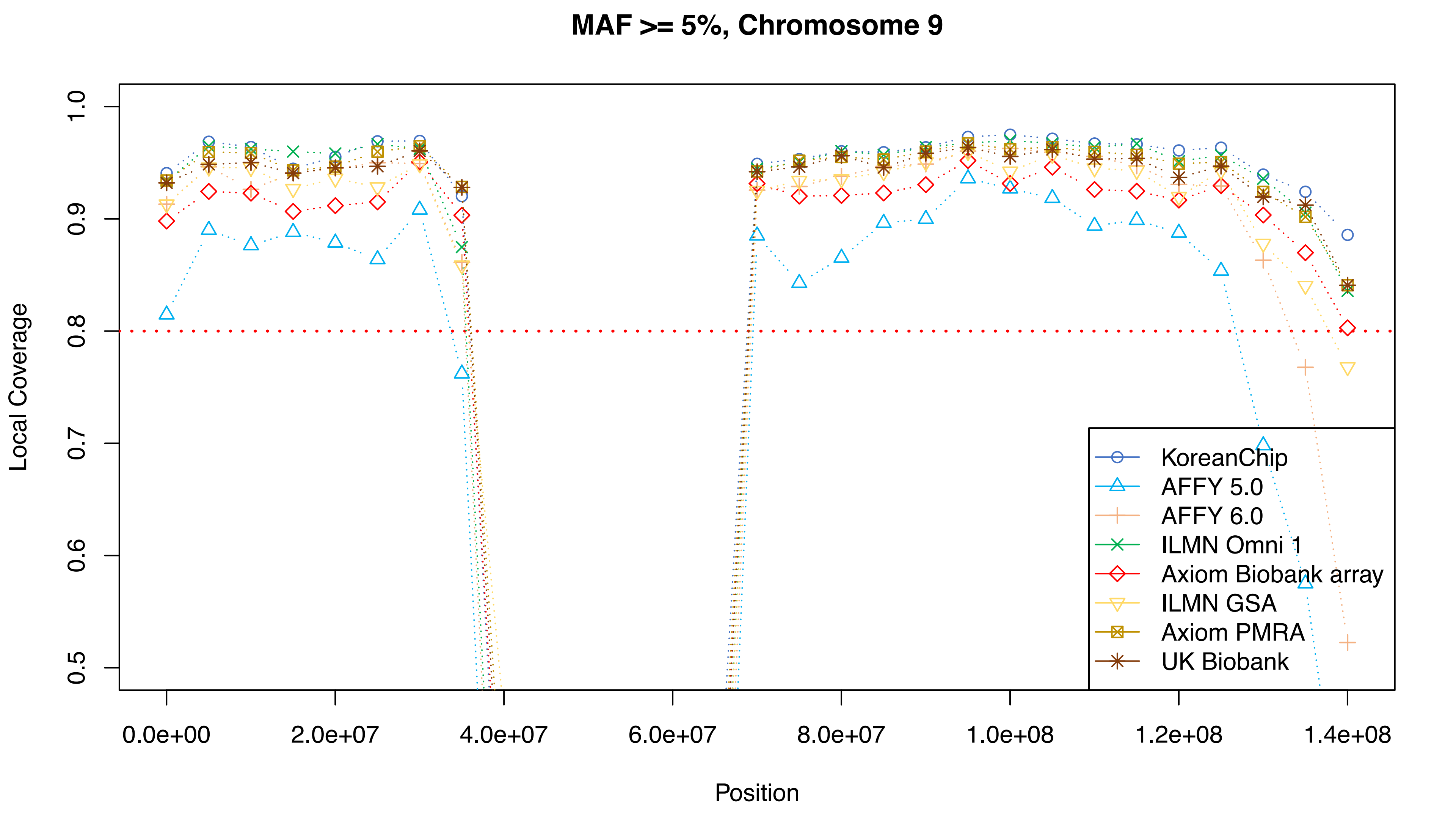

MAF >= 5%, Chromosome 9
Local Coverage
Position
1.0
0.9
0.8
0.7
0.6
0.5
0.0e+00
2.0e+07
4.0e+07
6.0e+07
8.0e+07
1.0e+08
1.2e+08
1.4e+08
KoreanChip
AFFY 5.0
AFFY 6.0
ILMN Omni 1
Axiom Biobank array
ILMN GSA
Axiom PMRA
UK Biobank

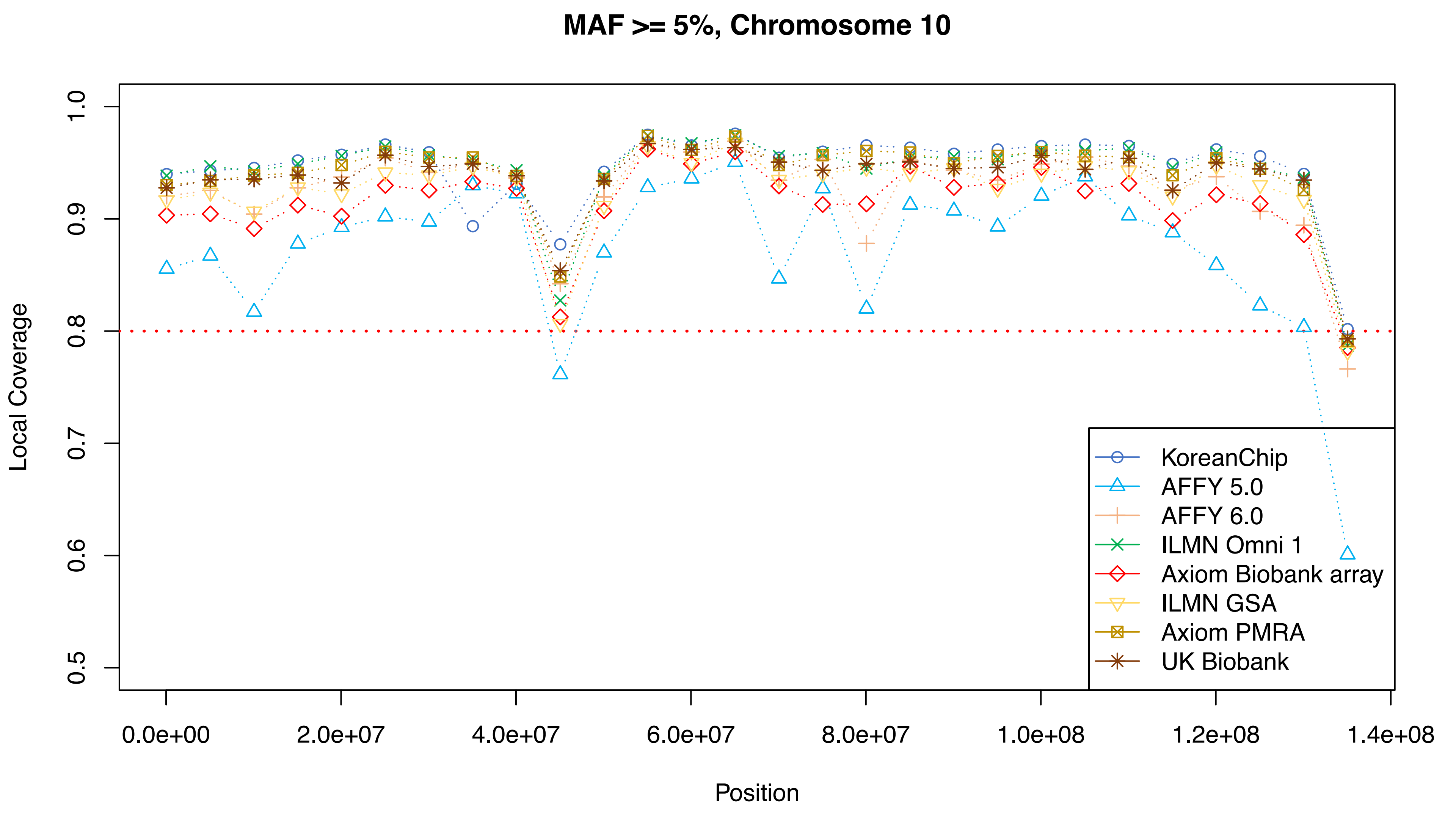
MAF >= 5%, Chromosome 10
Local Coverage
0.5
0.6
0.7
0.8
0.9
1.0
Position
0.0e+00
2.0e+07
4.0e+07
6.0e+07
8.0e+07
1.0e+08
1.2e+08
1.4e+08
KoreanChip
AFFY 5.0
AFFY 6.0
ILMN Omni 1
Axiom Biobank array
ILMN GSA
Axiom PMRA
UK Biobank

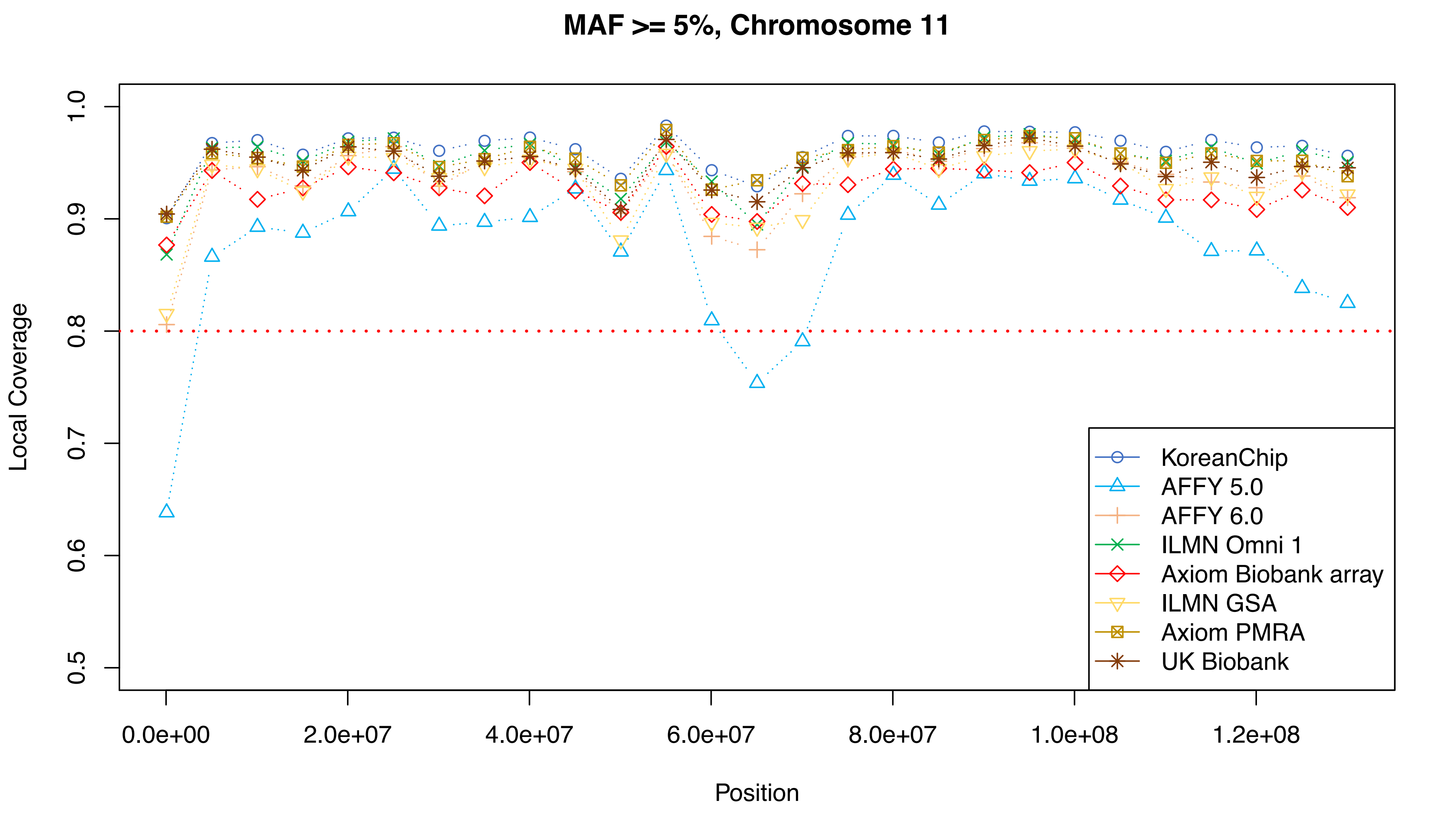
MAF >= 5%, Chromosome 11
Local Coverage
1.0
0.9
0.8
0.7
0.6
0.5
Position
0.0e+00
2.0e+07
4.0e+07
6.0e+07
8.0e+07
1.0e+08
1.2e+08
KoreanChip
AFFY 5.0
AFFY 6.0
ILMN Omni 1
Axiom Biobank array
ILMN GSA
Axiom PMRA
UK Biobank

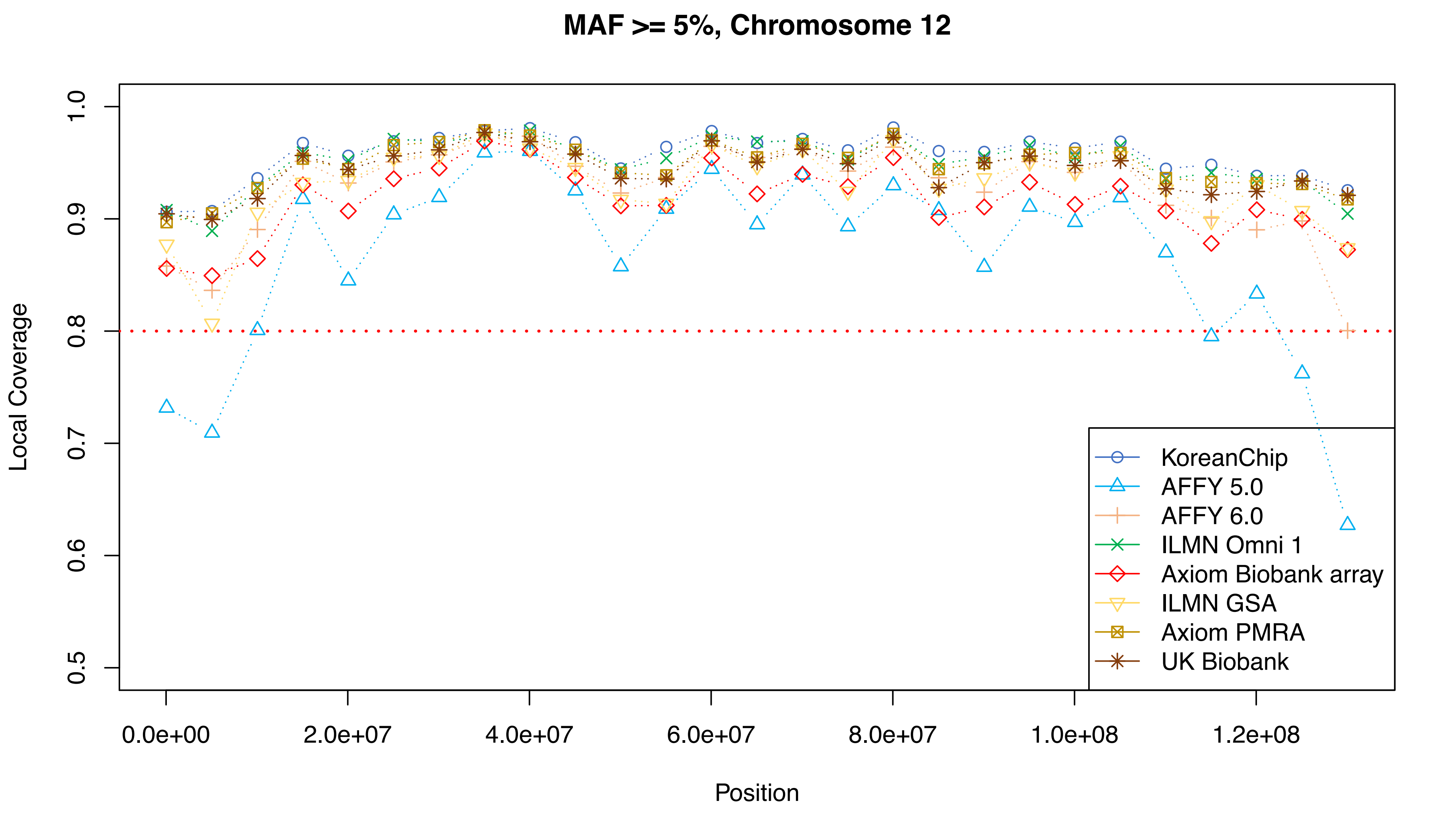

MAF >= 5%, Chromosome 12
Local Coverage
Position
0.5
0.6
0.7
0.8
0.9
1.0
0.0e+00
2.0e+07
4.0e+07
6.0e+07
8.0e+07
1.0e+08
1.2e+08
KoreanChip
AFFY 5.0
AFFY 6.0
ILMN Omni 1
Axiom Biobank array
ILMN GSA
Axiom PMRA
UK Biobank

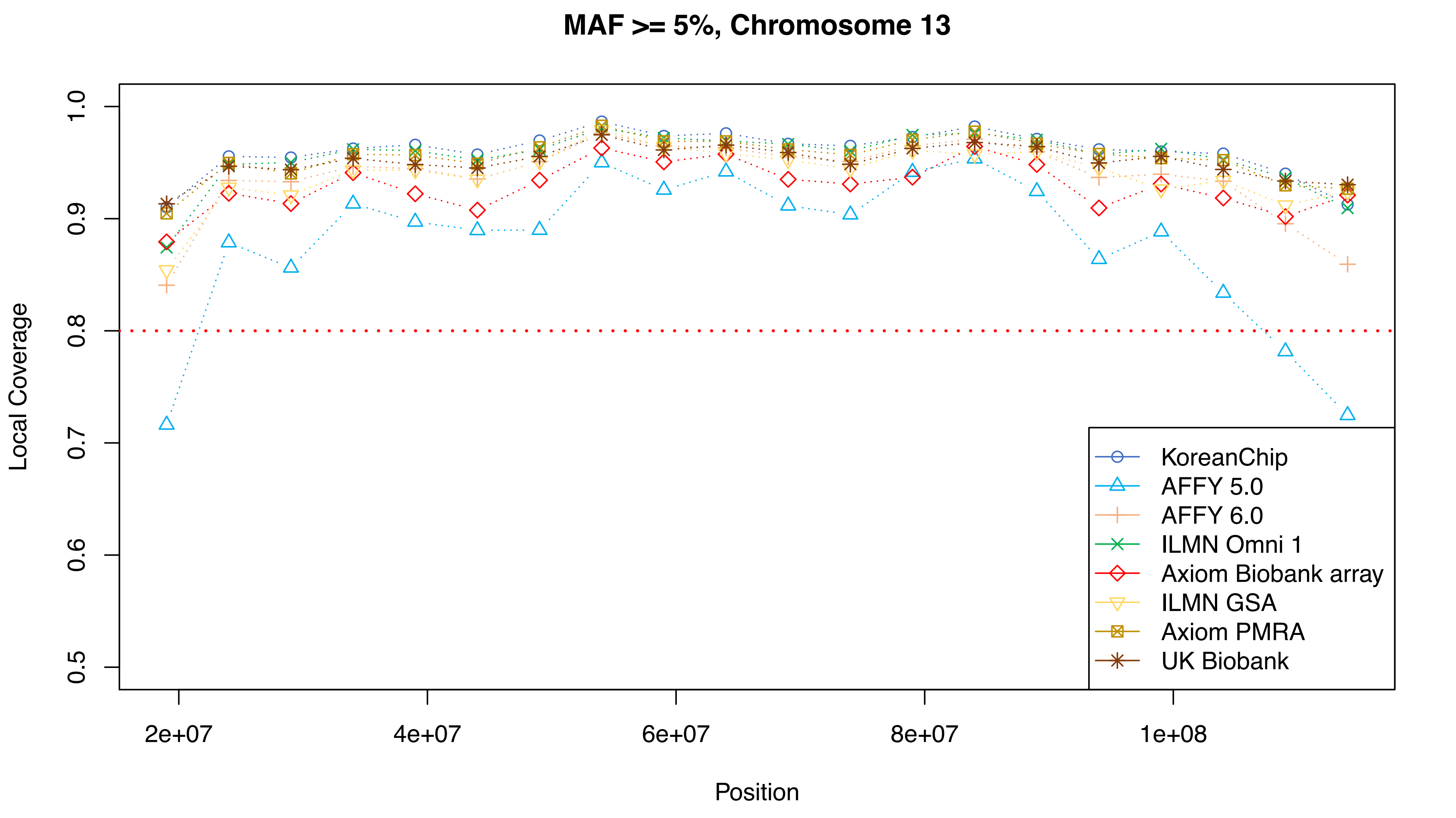

MAF >= 5%, Chromosome 13
Local Coverage
1.0
0.9
0.8
0.7
0.6
0.5
2e+07
4e+07
6e+07
8e+07
1e+08
Position
KoreanChip
AFFY 5.0
AFFY 6.0
ILMN Omni 1
Axiom Biobank array
ILMN GSA
Axiom PMRA
UK Biobank

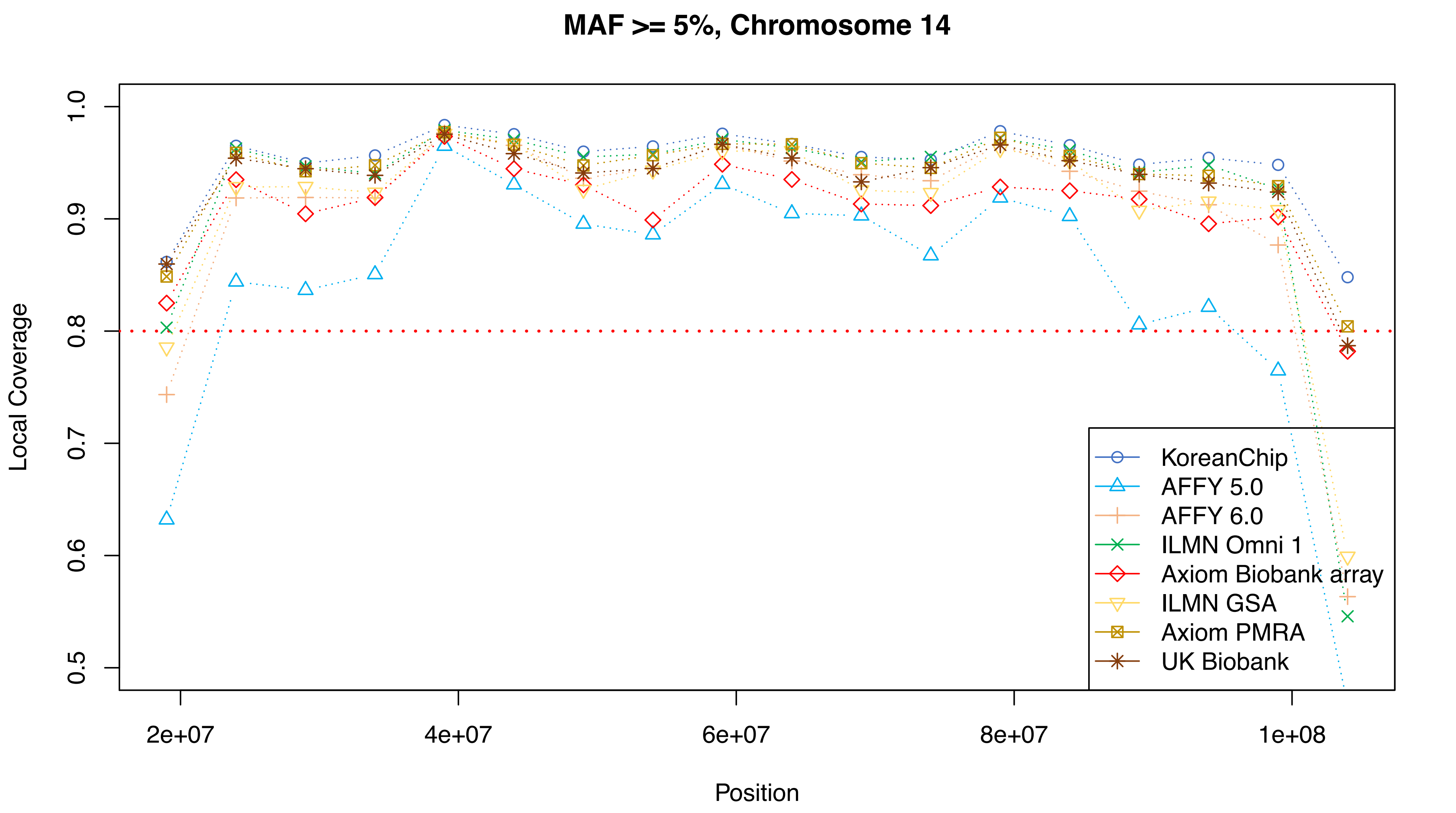
MAF >= 5%, Chromosome 14
Local Coverage
1.0
0.9
0.8
0.7
0.6
0.5
Position
2e+07
4e+07
6e+07
8e+07
1e+08
KoreanChip
AFFY 5.0
AFFY 6.0
ILMN Omni 1
Axiom Biobank array
ILMN GSA
Axiom PMRA
UK Biobank

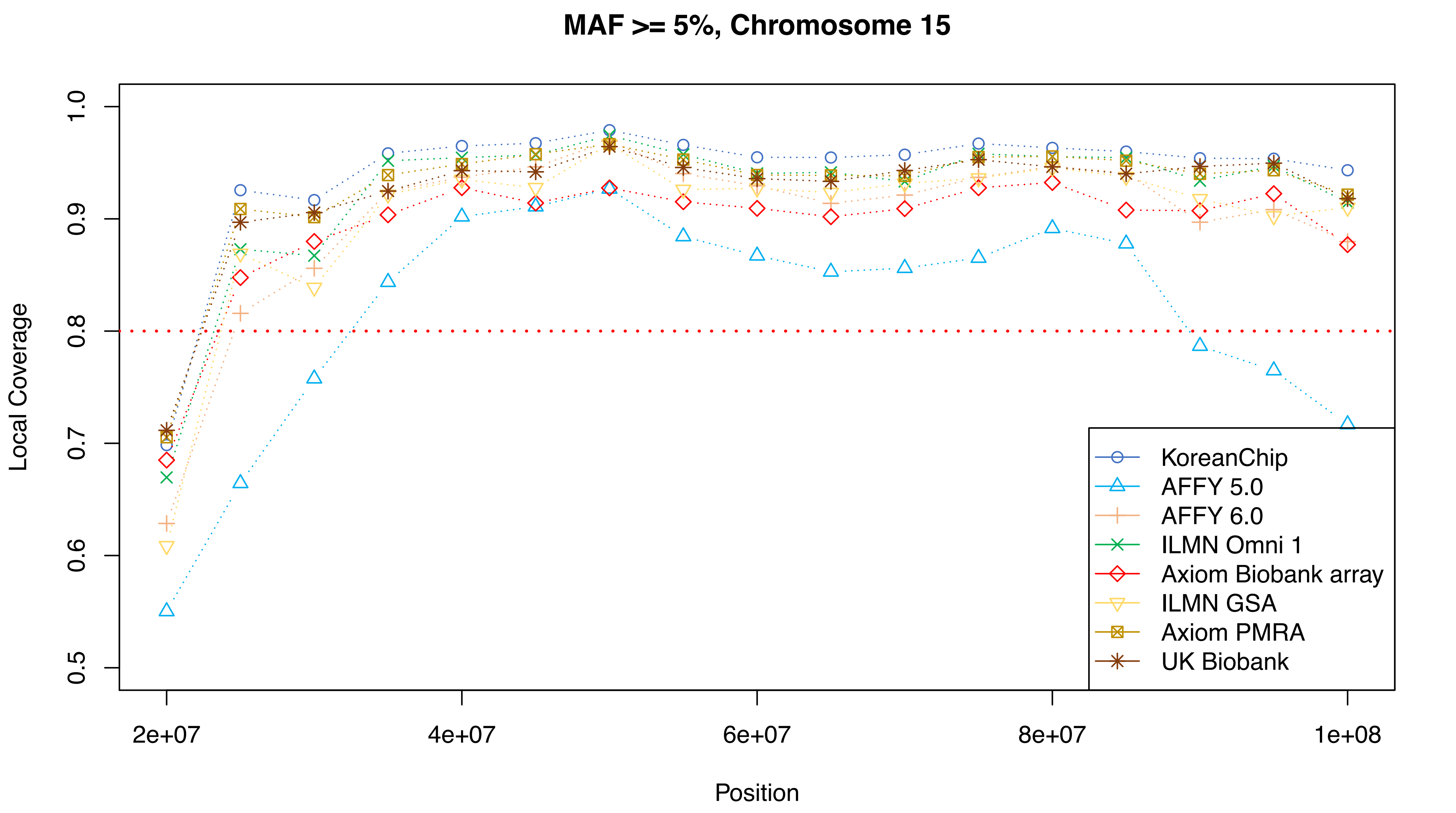
MAF >= 5%, Chromosome 15
Local Coverage
Position
1.0
0.9
0.8
0.7
0.6
0.5
2e+07
4e+07
6e+07
8e+07
1e+08
KoreanChip
AFFY 5.0
AFFY 6.0
ILMN Omni 1
Axiom Biobank array
ILMN GSA
Axiom PMRA
UK Biobank

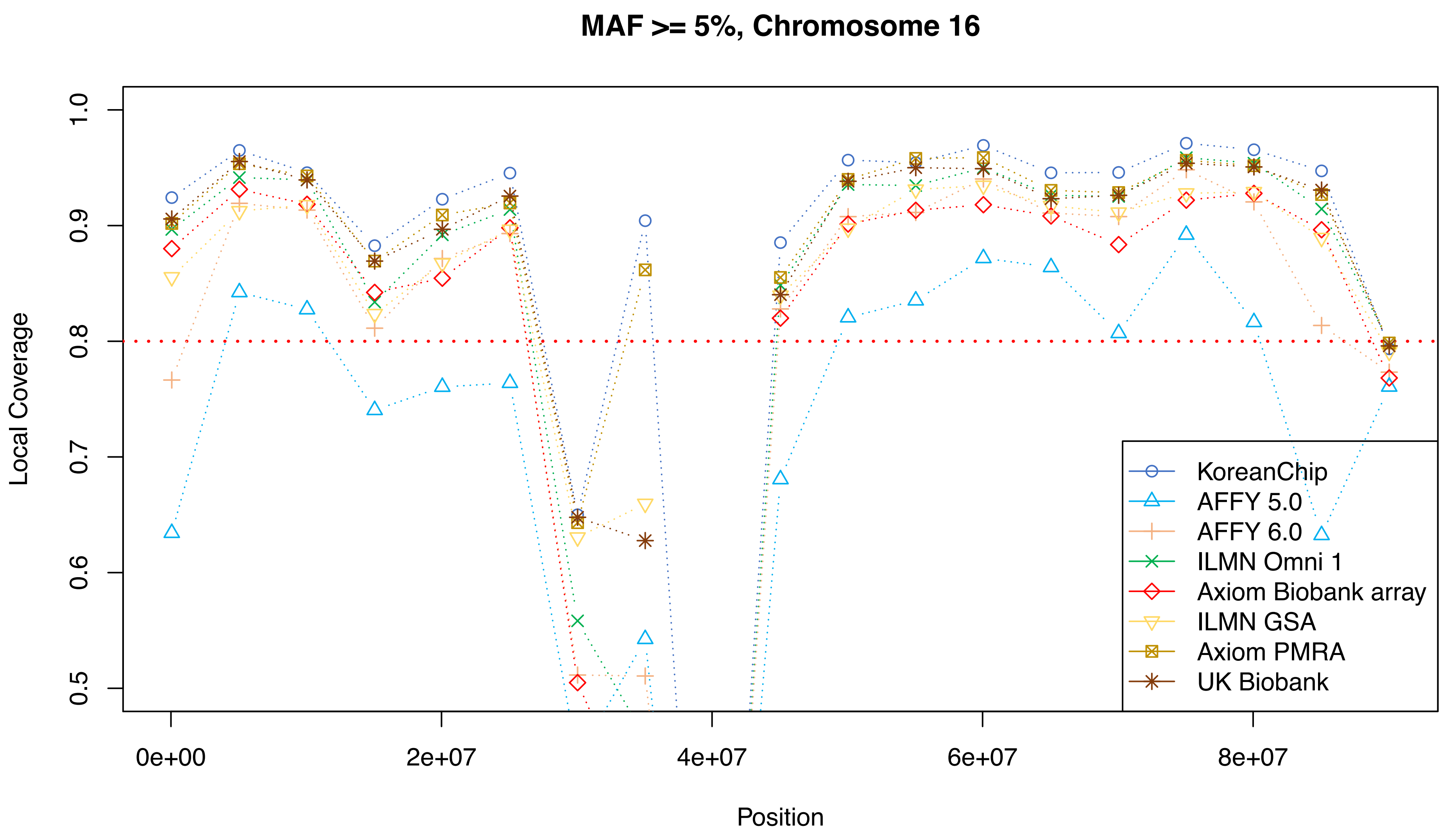
MAF >= 5%, Chromosome 16
Local Coverage
1.0
0.9
0.8
0.7
0.6
0.5
0e+00
2e+07
4e+07
6e+07
8e+07
Position
KoreanChip
AFFY 5.0
AFFY 6.0
ILMN Omni 1
Axiom Biobank array
ILMN GSA
Axiom PMRA
UK Biobank

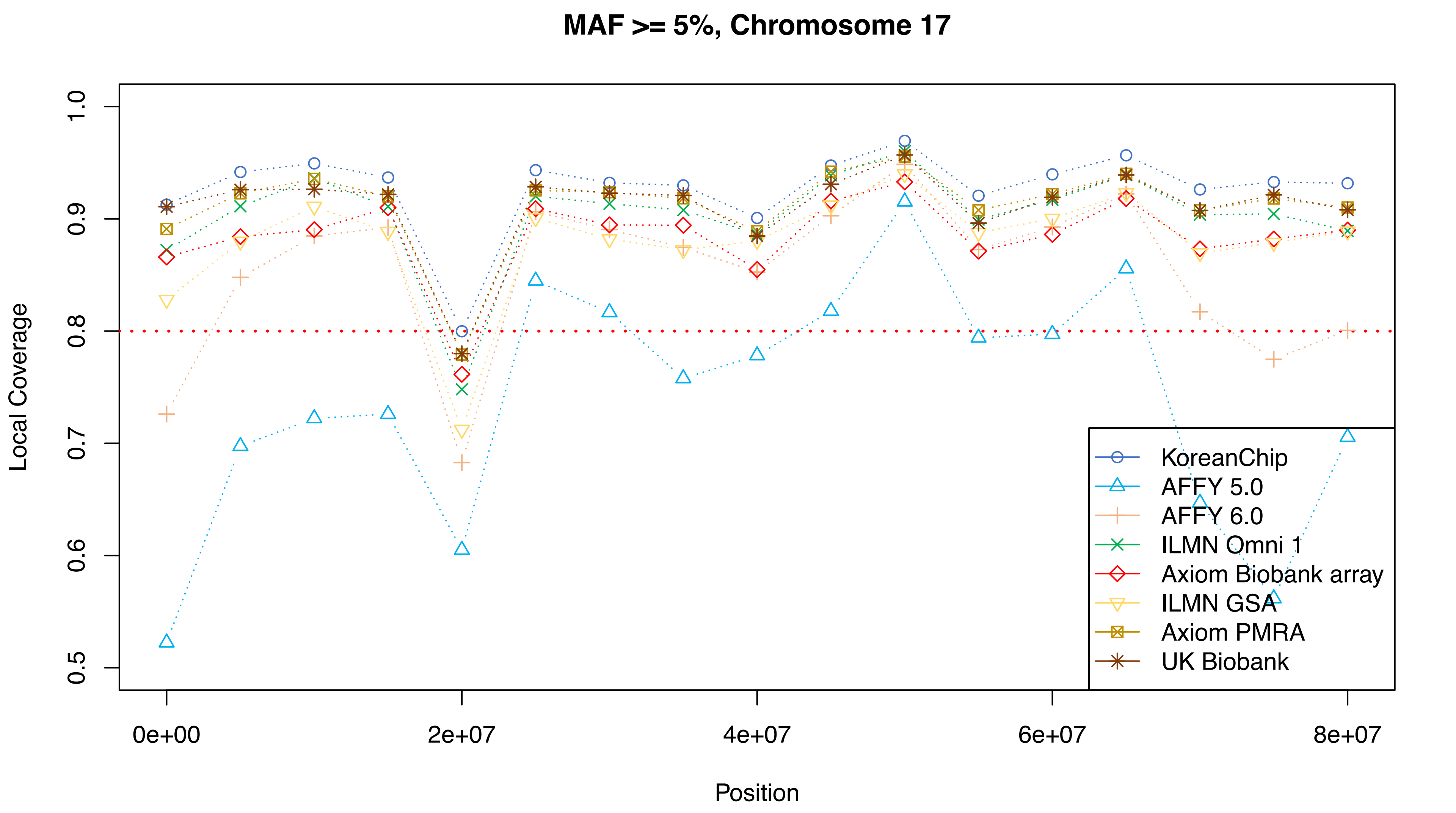

MAF >= 5%, Chromosome 17
Local Coverage
Position
1.0
0.9
0.8
0.7
0.6
0.5
0e+00
2e+07
4e+07
6e+07
8e+07
KoreanChip
AFFY 5.0
AFFY 6.0
ILMN Omni 1
Axiom Biobank array
ILMN GSA
Axiom PMRA
UK Biobank

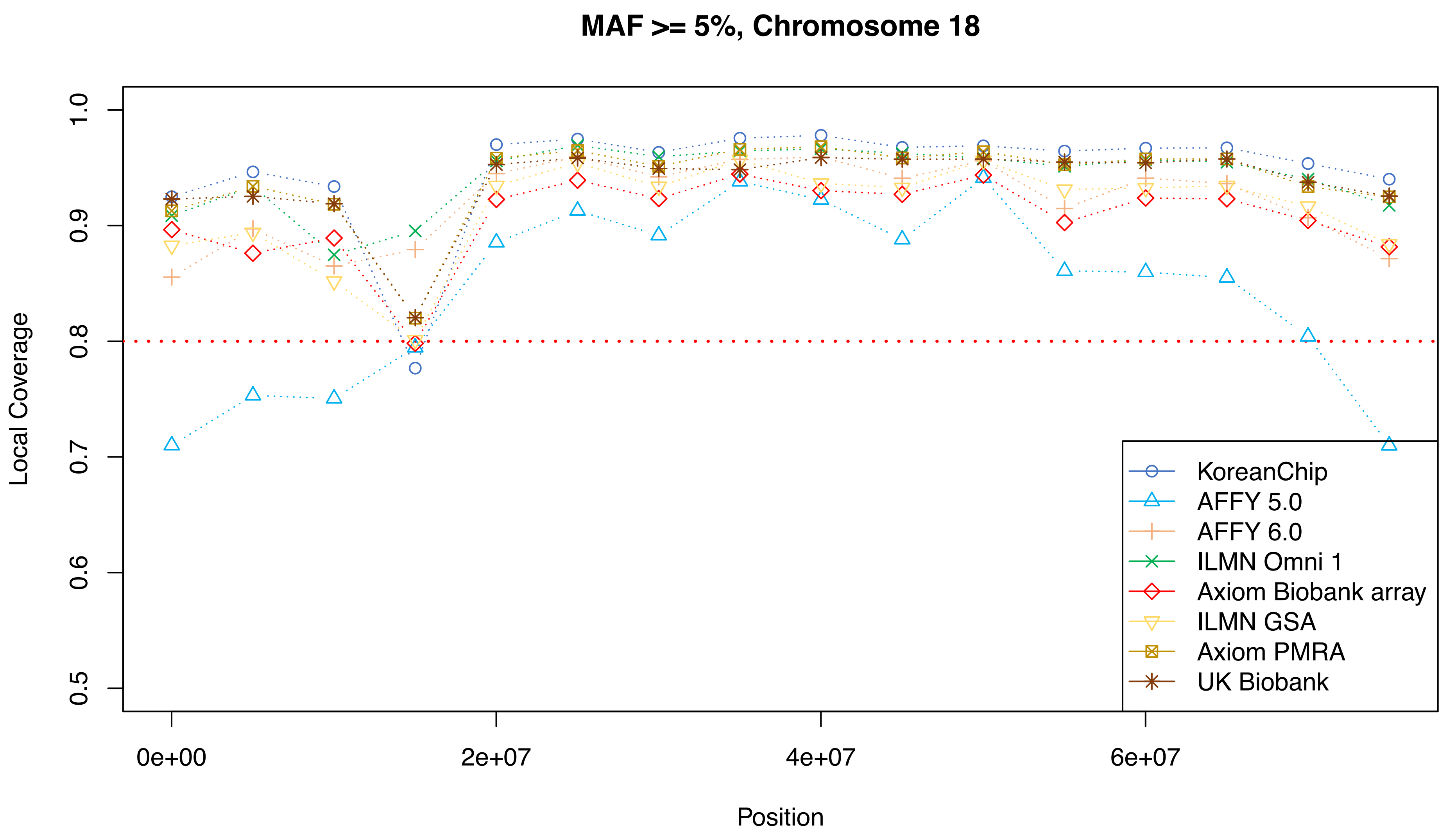

MAF >= 5%, Chromosome 18
Local Coverage
Position
0.5
0.6
0.7
0.8
0.9
1.0
0e+00
2e+07
4e+07
6e+07
KoreanChip
AFFY 5.0
AFFY 6.0
ILMN Omni 1
Axiom Biobank array
ILMN GSA
Axiom PMRA
UK Biobank

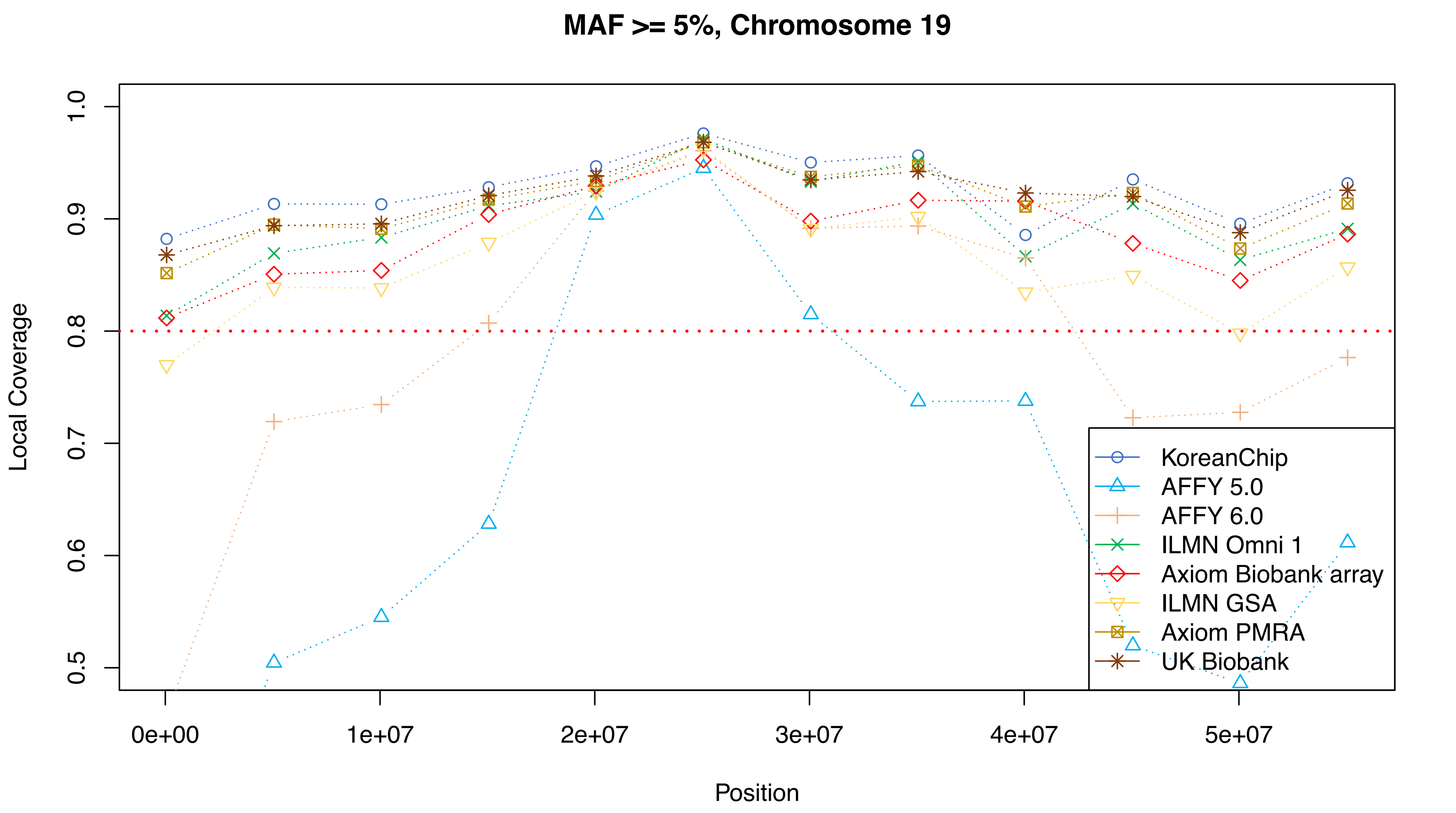

MAF >= 5%, Chromosome 19
Local Coverage
Position
0.5
0.6
0.7
0.8
0.9
1.0
0e+00
1e+07
2e+07
3e+07
4e+07
5e+07
KoreanChip
AFFY 5.0
AFFY 6.0
ILMN Omni 1
Axiom Biobank array
ILMN GSA
Axiom PMRA
UK Biobank

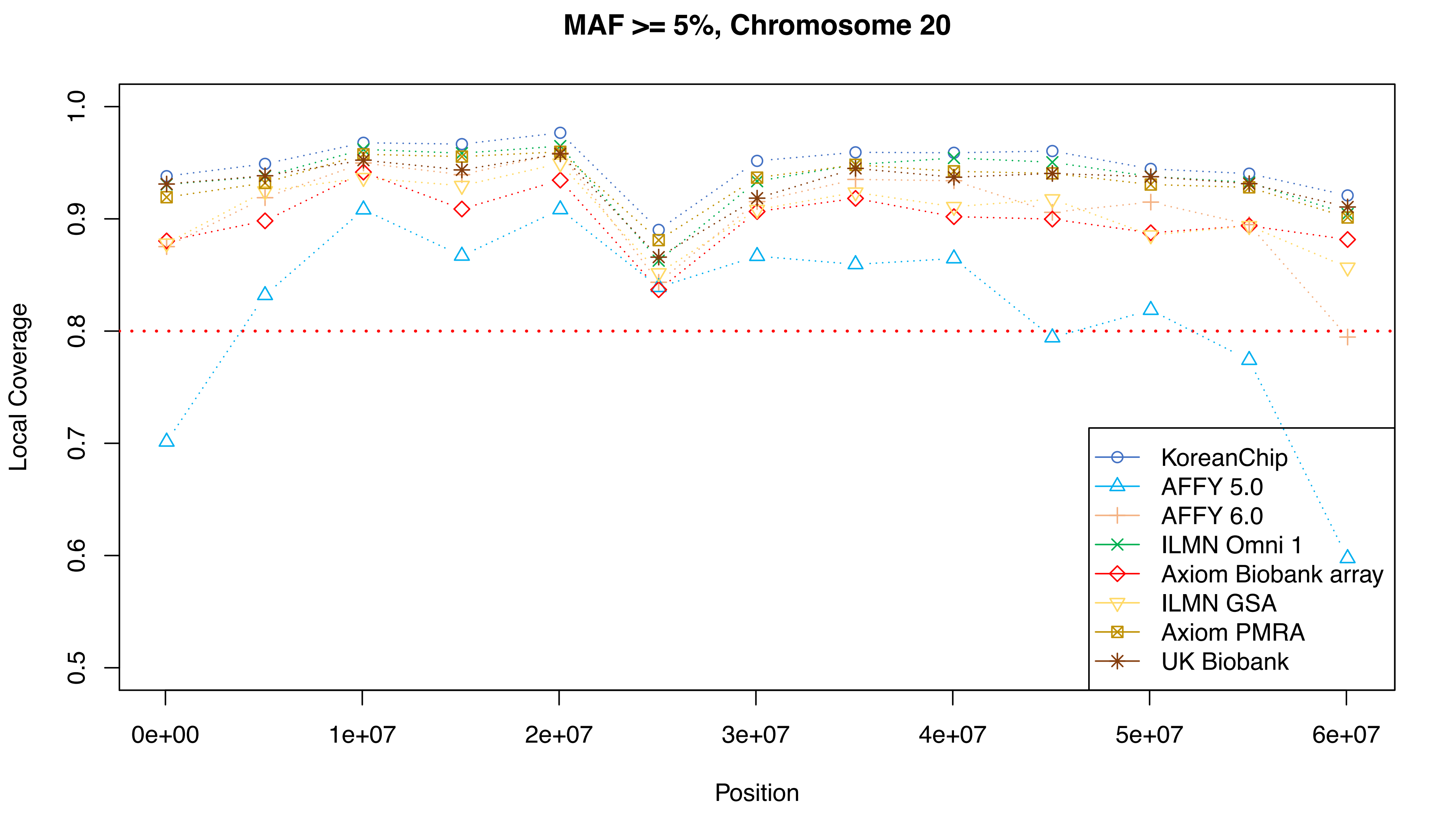

MAF >= 5%, Chromosome 20
Local Coverage
Position
1.0
0.9
0.8
0.7
0.6
0.5
0e+00
1e+07
2e+07
3e+07
4e+07
5e+07
6e+07
KoreanChip
AFFY 5.0
AFFY 6.0
ILMN Omni 1
Axiom Biobank array
ILMN GSA
Axiom PMRA
UK Biobank

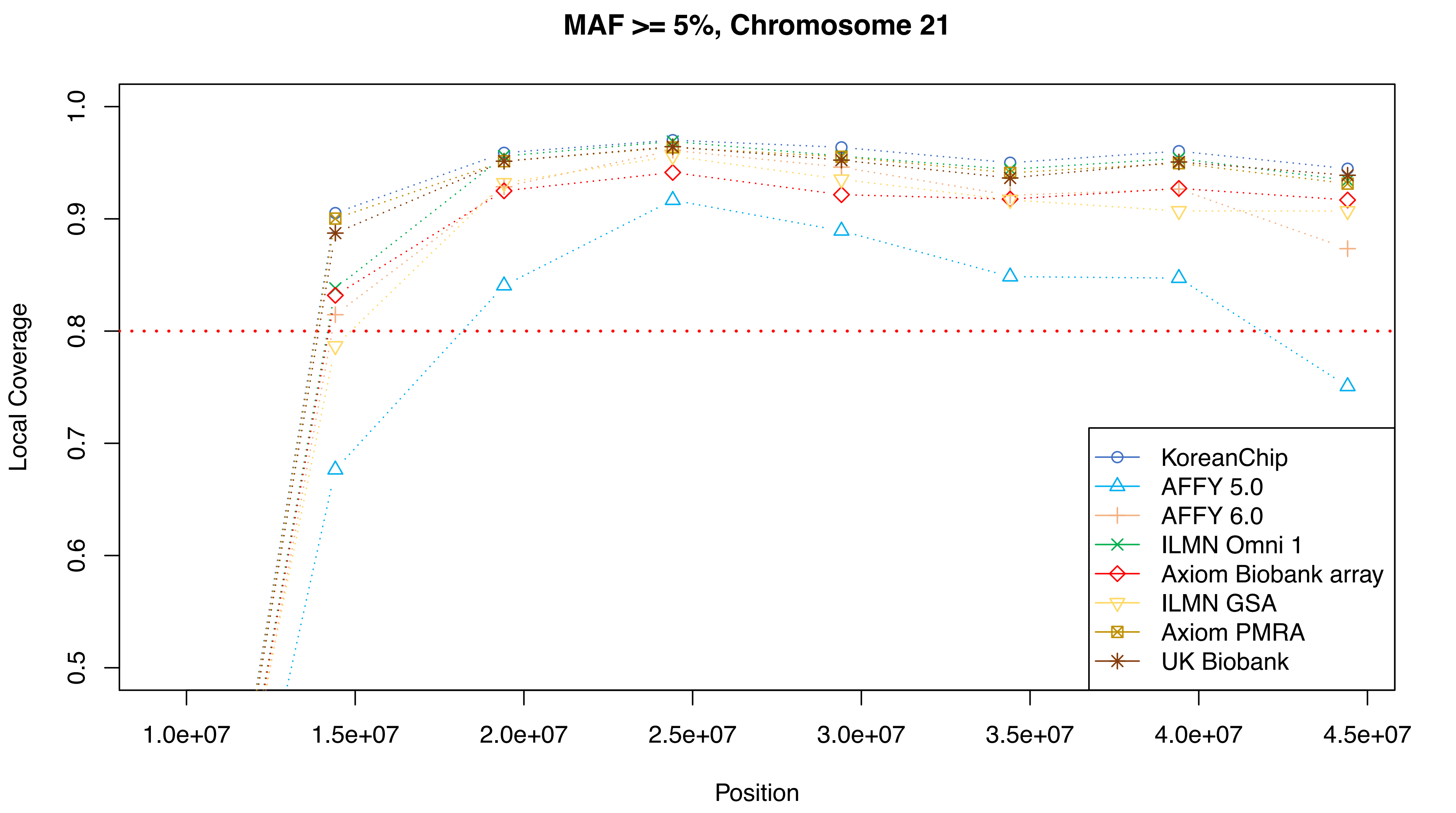
MAF >= 5%, Chromosome 21
Local Coverage
1.0
0.9
0.8
0.7
0.6
0.5
1.0e+07
1.5e+07
2.0e+07
2.5e+07
3.0e+07
3.5e+07
4.0e+07
4.5e+07
Position
KoreanChip
AFFY 5.0
AFFY 6.0
ILMN Omni 1
Axiom Biobank array
ILMN GSA
Axiom PMRA
UK Biobank

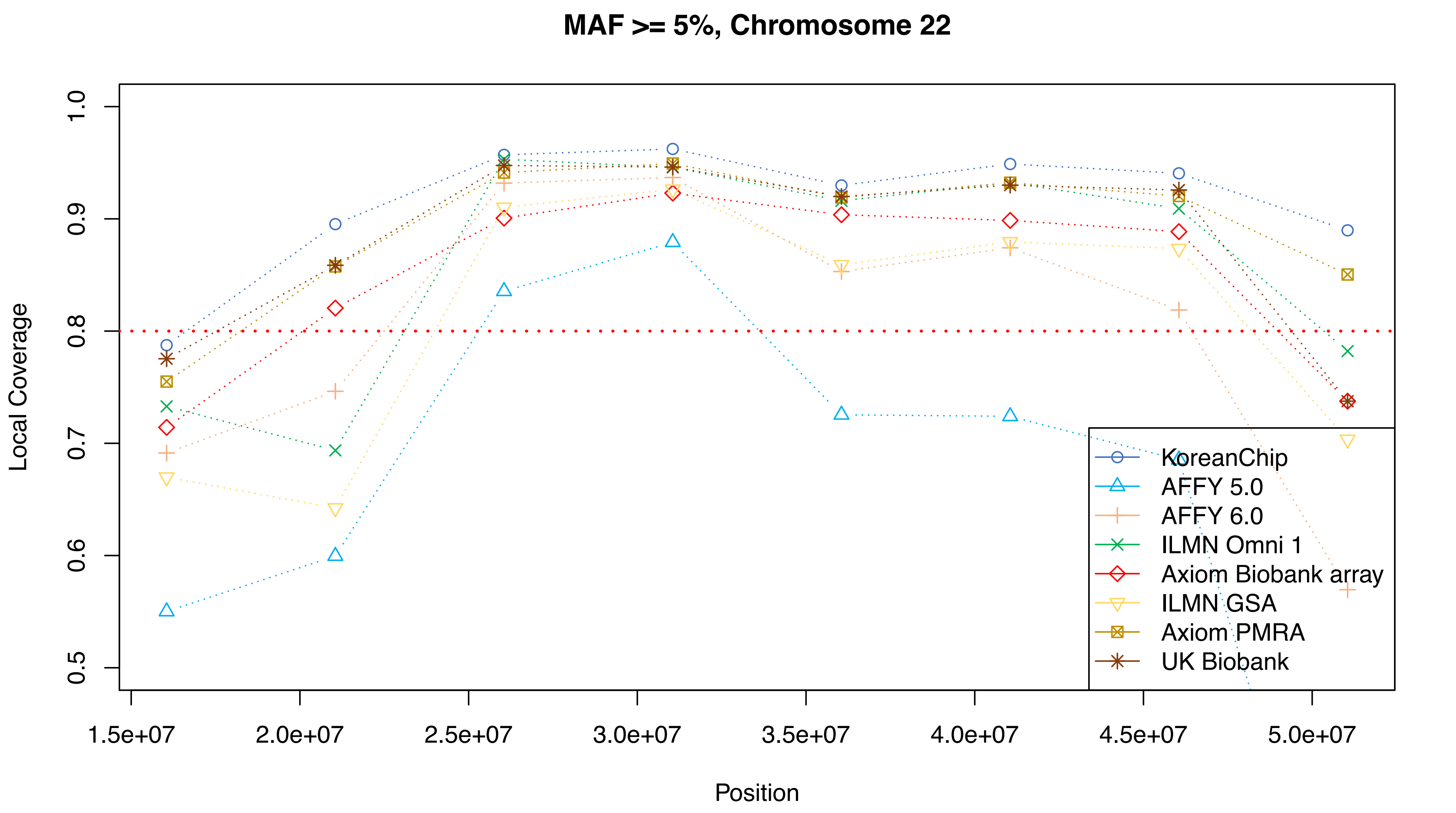

MAF >= 5%, Chromosome 22
Local Coverage
Position
1.0
0.9
0.8
0.7
0.6
0.5
1.5e+07
2.0e+07
2.5e+07
3.0e+07
3.5e+07
4.0e+07
4.5e+07
5.0e+07
KoreanChip
AFFY 5.0
AFFY 6.0
ILMN Omni 1
Axiom Biobank array
ILMN GSA
Axiom PMRA
UK Biobank

Fig. S3. Genomic coverage of each chromosome (MAF < 5%).

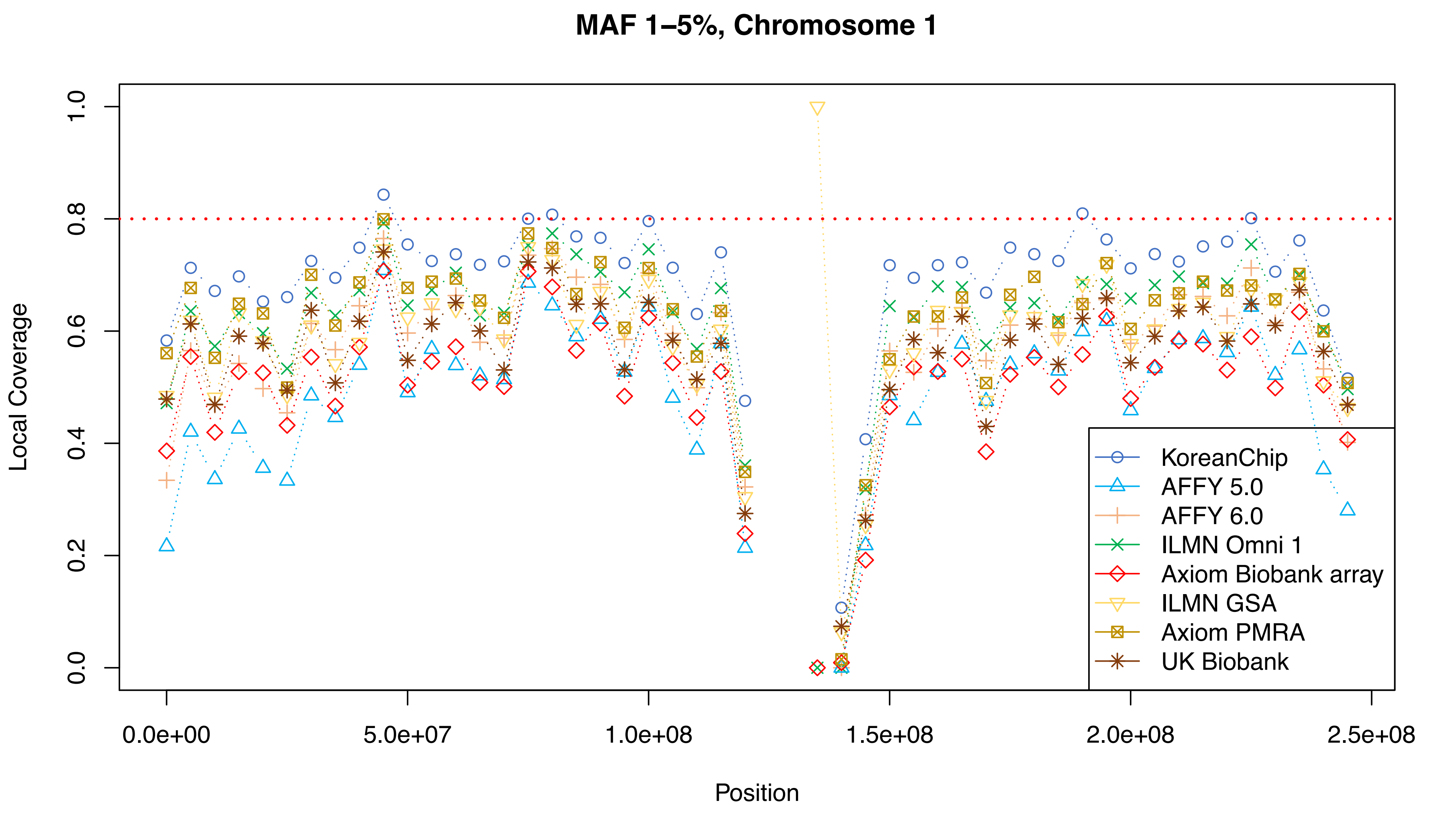
MAF 1–5%, Chromosome 1
Local Coverage
0.0
0.2
0.4
0.6
0.8
1.0
Position
0.0e+00
5.0e+07
1.0e+08
1.5e+08
2.0e+08
2.5e+08
KoreanChip
AFFY 5.0
AFFY 6.0
ILMN Omni 1
Axiom Biobank array
ILMN GSA
Axiom PMRA
UK Biobank

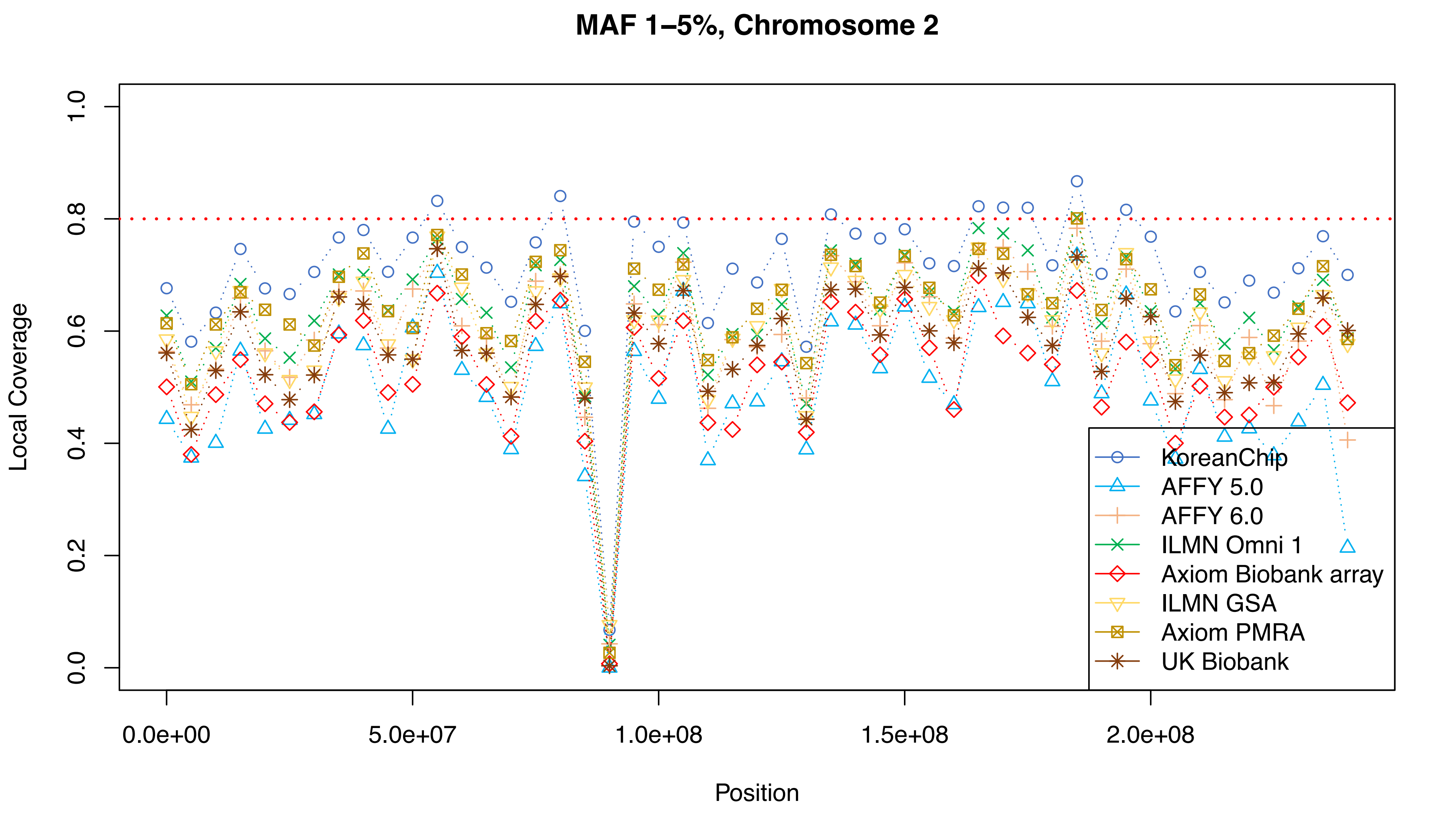

MAF 1–5%, Chromosome 2
Local Coverage
Position
0.0
0.2
0.4
0.6
0.8
1.0
0.0e+00
5.0e+07
1.0e+08
1.5e+08
2.0e+08
KoreanChip
AFFY 5.0
AFFY 6.0
ILMN Omni 1
Axiom Biobank array
ILMN GSA
Axiom PMRA
UK Biobank

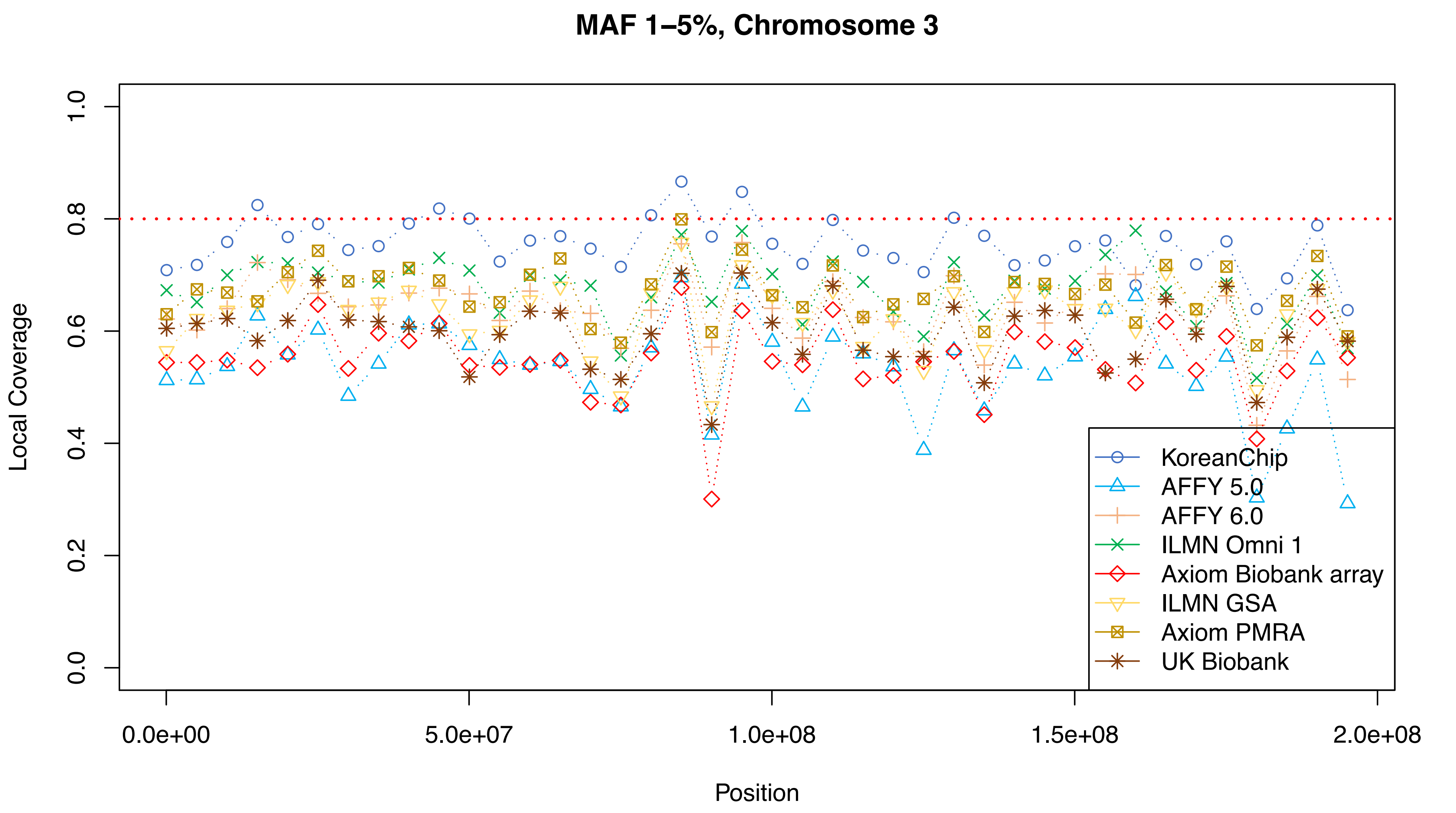
MAF 1–5%, Chromosome 3
Local Coverage
Position
0.0
0.2
0.4
0.6
0.8
1.0
0.0e+00
5.0e+07
1.0e+08
1.5e+08
2.0e+08
KoreanChip
AFFY 5.0
AFFY 6.0
ILMN Omni 1
Axiom Biobank array
ILMN GSA
Axiom PMRA
UK Biobank

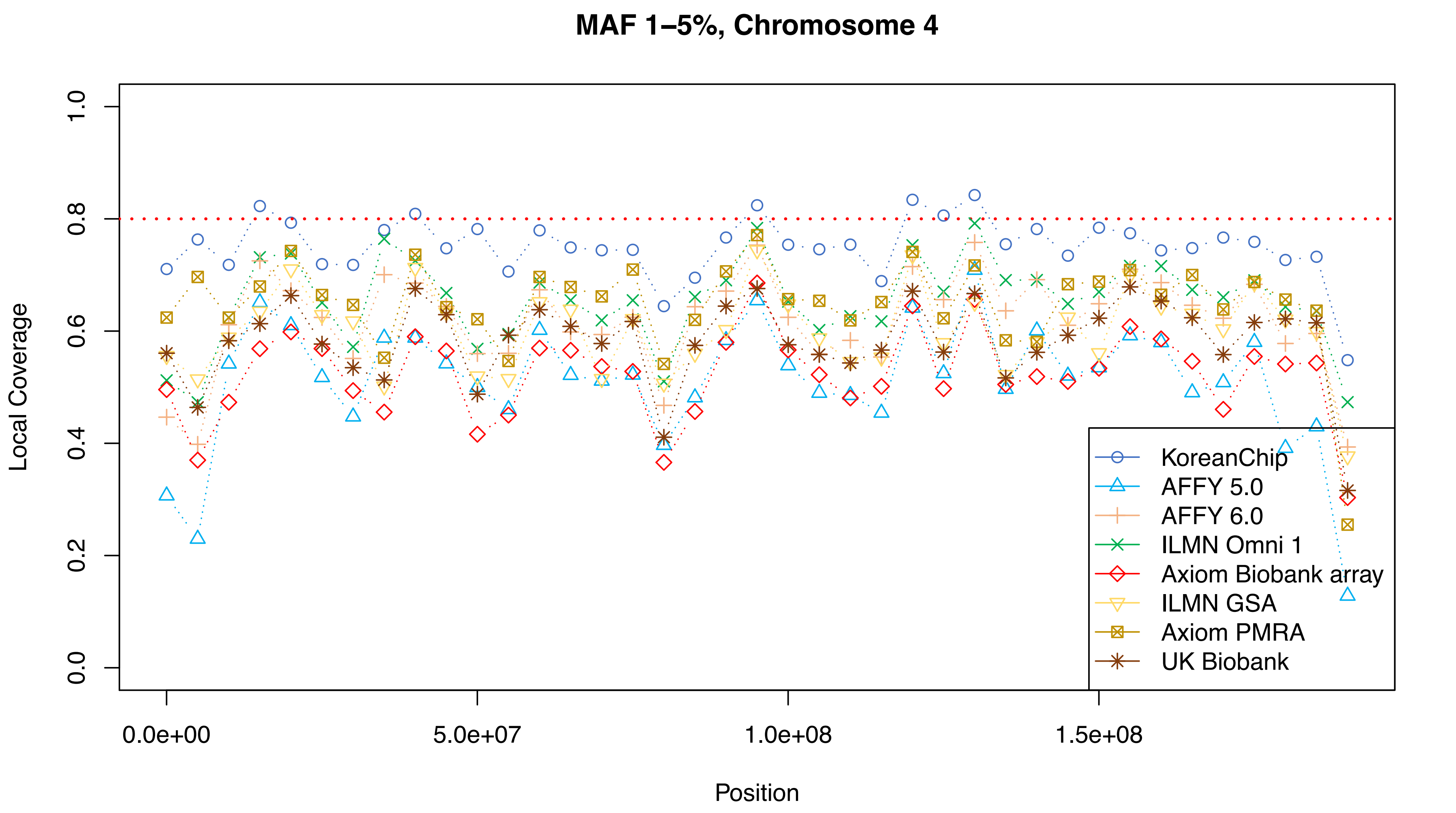

MAF 1–5%, Chromosome 4
Local Coverage
0.0
0.2
0.4
0.6
0.8
1.0
Position
0.0e+00
5.0e+07
1.0e+08
1.5e+08
KoreanChip
AFFY 5.0
AFFY 6.0
ILMN Omni 1
Axiom Biobank array
ILMN GSA
Axiom PMRA
UK Biobank

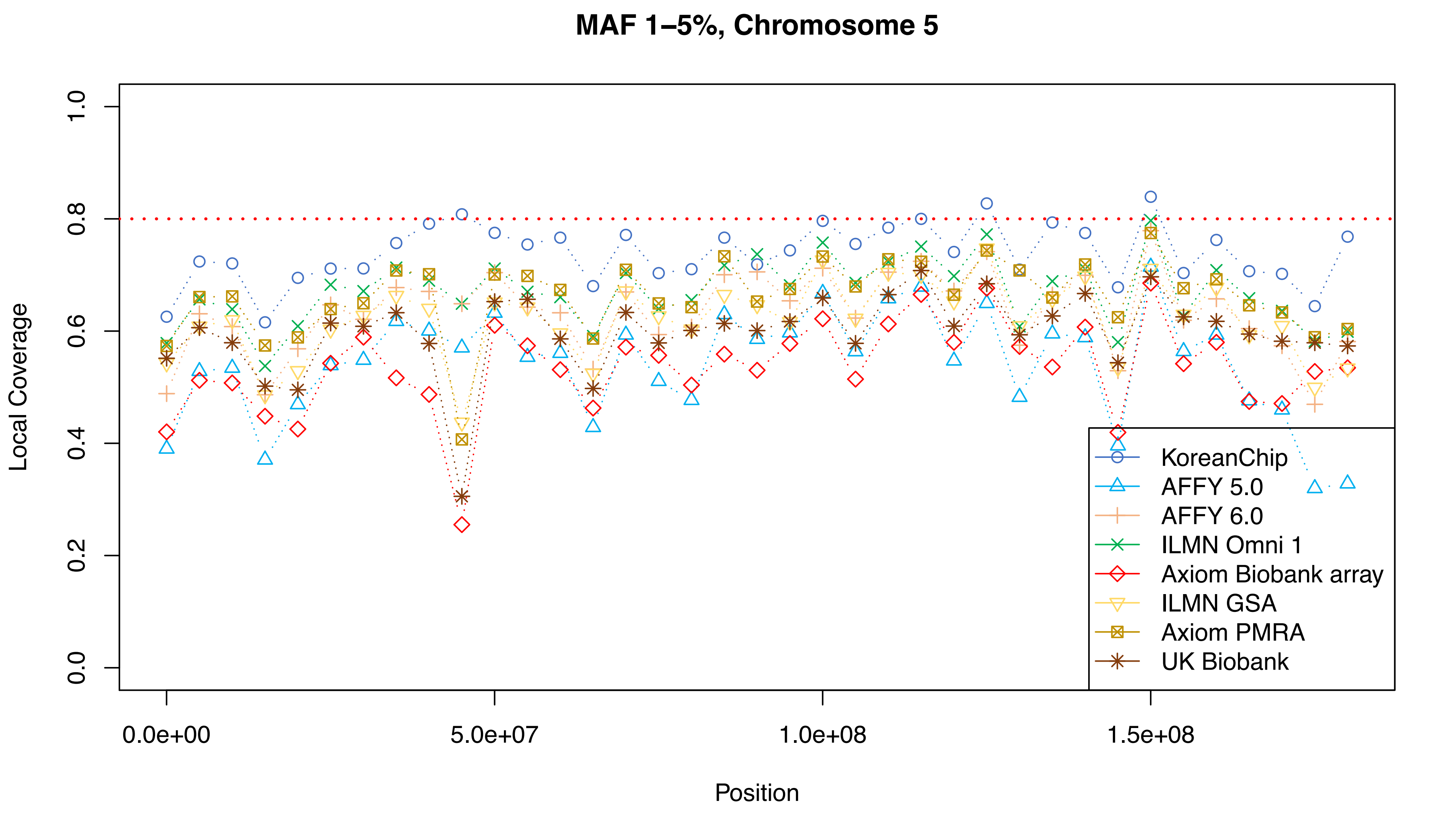
MAF 1–5%, Chromosome 5
Local Coverage
Position
0.0
0.2
0.4
0.6
0.8
1.0
0.0e+00
5.0e+07
1.0e+08
1.5e+08
KoreanChip
AFFY 5.0
AFFY 6.0
ILMN Omni 1
Axiom Biobank array
ILMN GSA
Axiom PMRA
UK Biobank

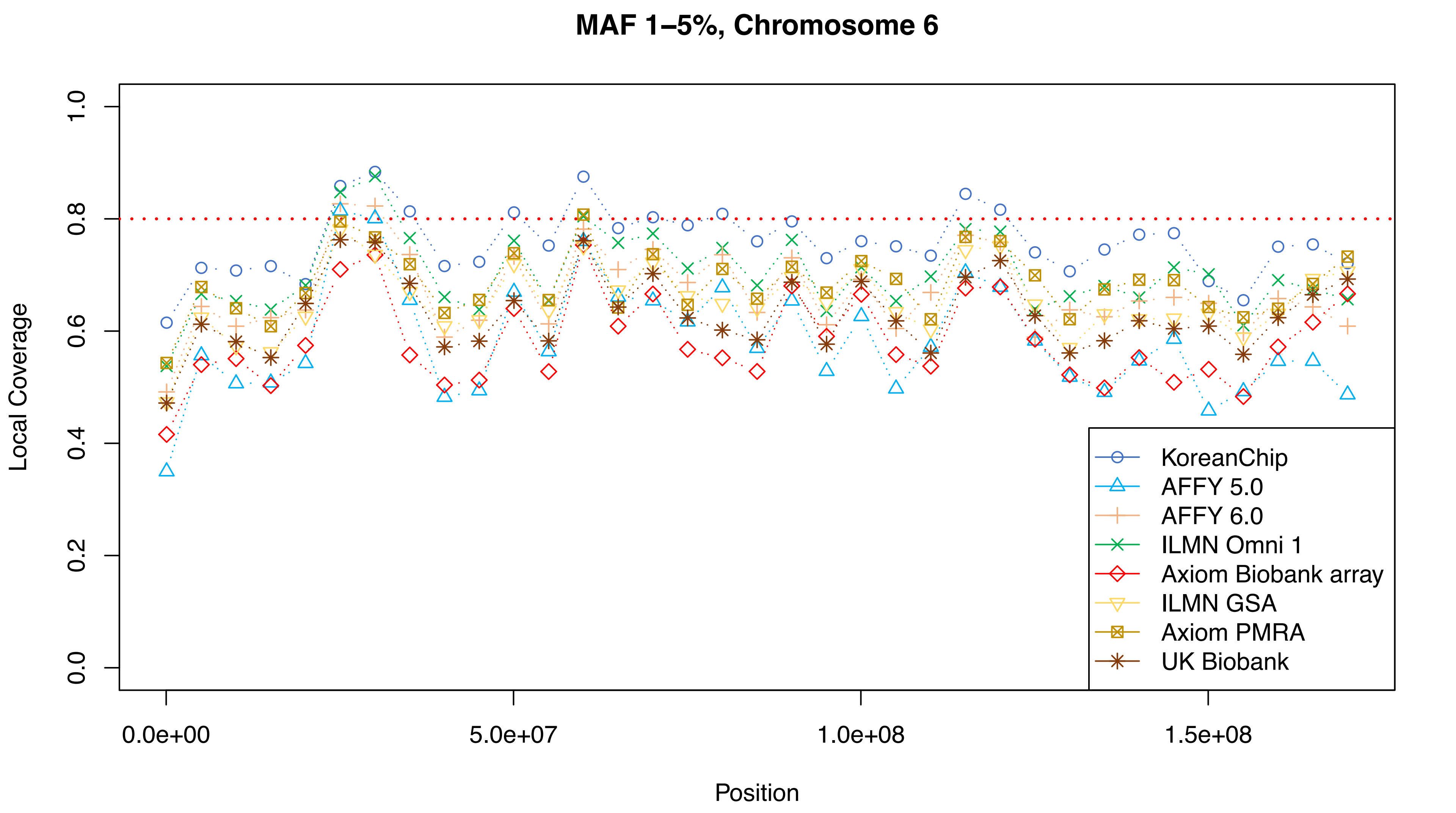

MAF 1–5%, Chromosome 6
Local Coverage
Position
0.0
0.2
0.4
0.6
0.8
1.0
0.0e+00
5.0e+07
1.0e+08
1.5e+08
KoreanChip
AFFY 5.0
AFFY 6.0
ILMN Omni 1
Axiom Biobank array
ILMN GSA
Axiom PMRA
UK Biobank

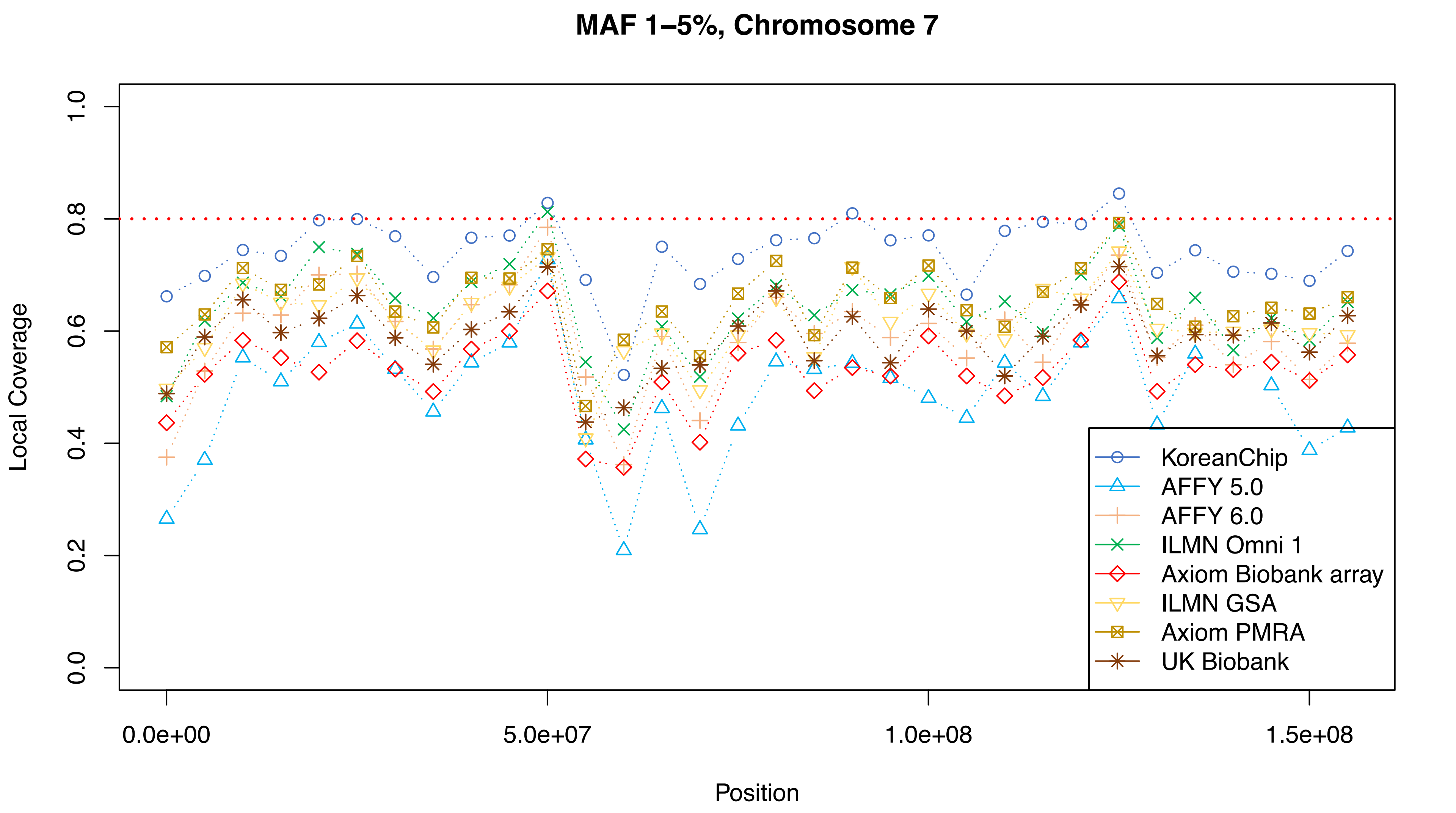

MAF 1–5%, Chromosome 7
Local Coverage
0.0
0.2
0.4
0.6
0.8
1.0
Position
0.0e+00
5.0e+07
1.0e+08
1.5e+08
KoreanChip
AFFY 5.0
AFFY 6.0
ILMN Omni 1
Axiom Biobank array
ILMN GSA
Axiom PMRA
UK Biobank

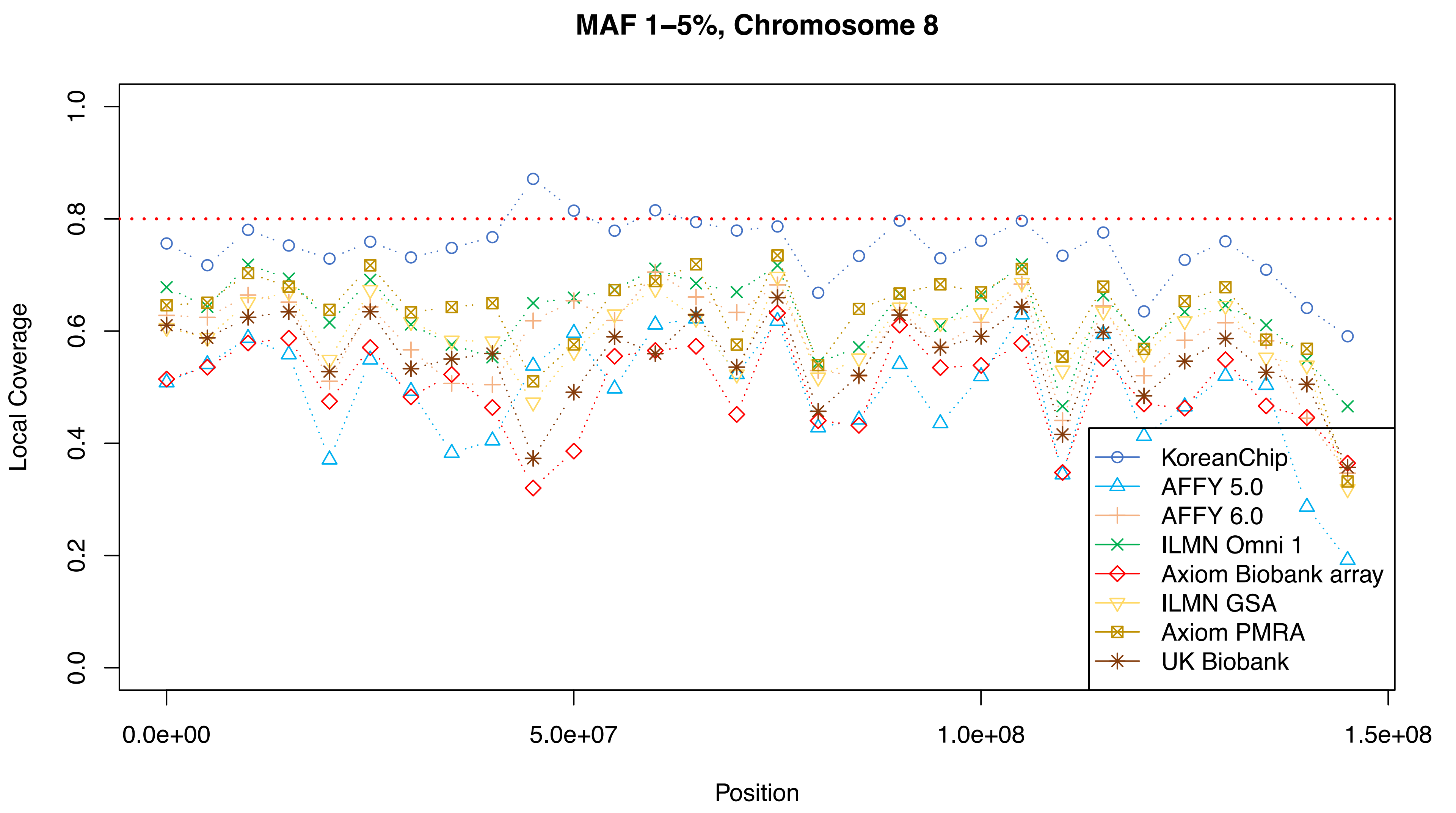
MAF 1–5%, Chromosome 8
Local Coverage
Position
0.0
0.2
0.4
0.6
0.8
1.0
0.0e+00
5.0e+07
1.0e+08
1.5e+08
KoreanChip
AFFY 5.0
AFFY 6.0
ILMN Omni 1
Axiom Biobank array
ILMN GSA
Axiom PMRA
UK Biobank

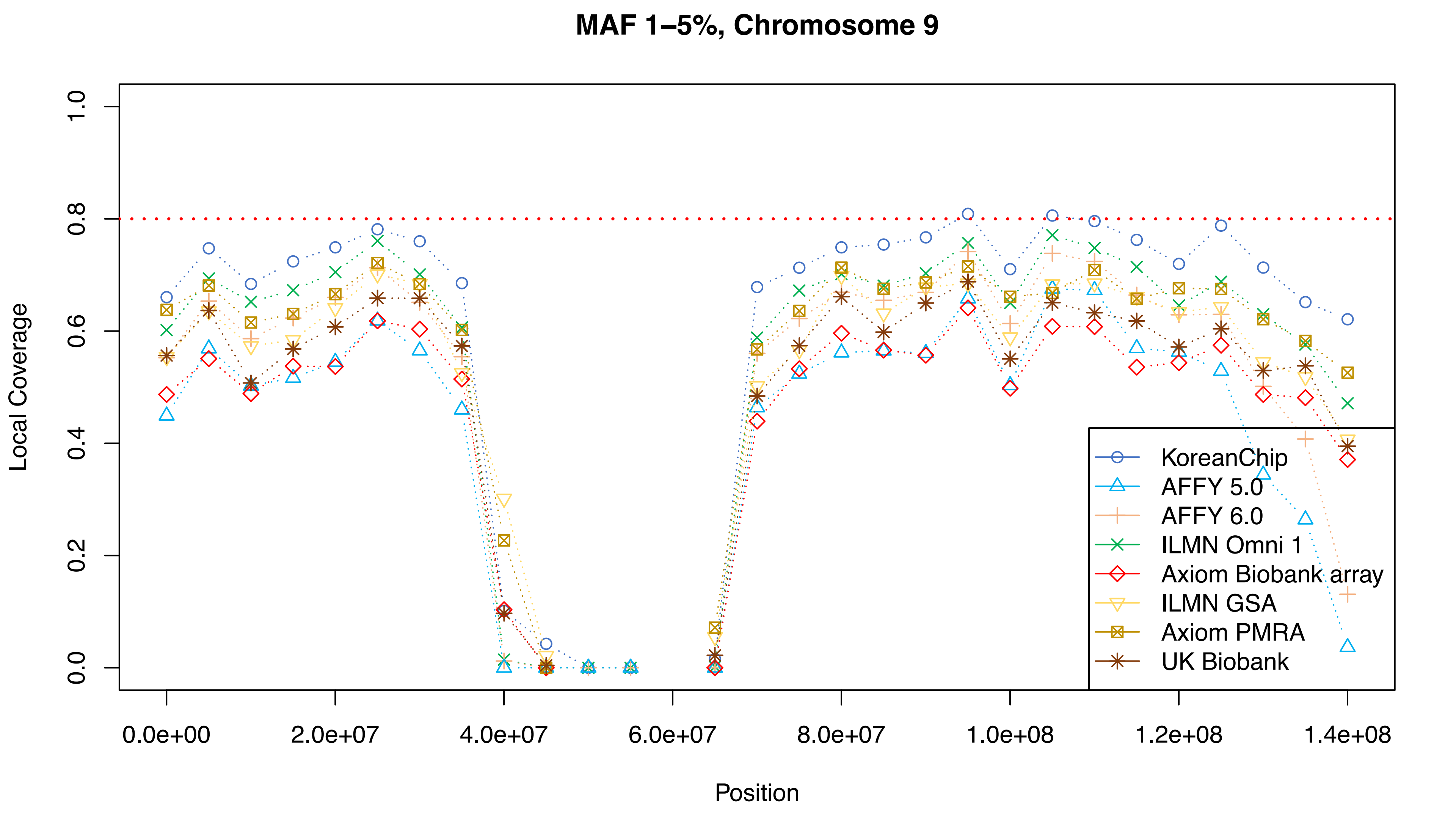
MAF 1–5%, Chromosome 9
Local Coverage
Position
0.0
0.2
0.4
0.6
0.8
1.0
0.0e+00
2.0e+07
4.0e+07
6.0e+07
8.0e+07
1.0e+08
1.2e+08
1.4e+08
KoreanChip
AFFY 5.0
AFFY 6.0
ILMN Omni 1
Axiom Biobank array
ILMN GSA
Axiom PMRA
UK Biobank

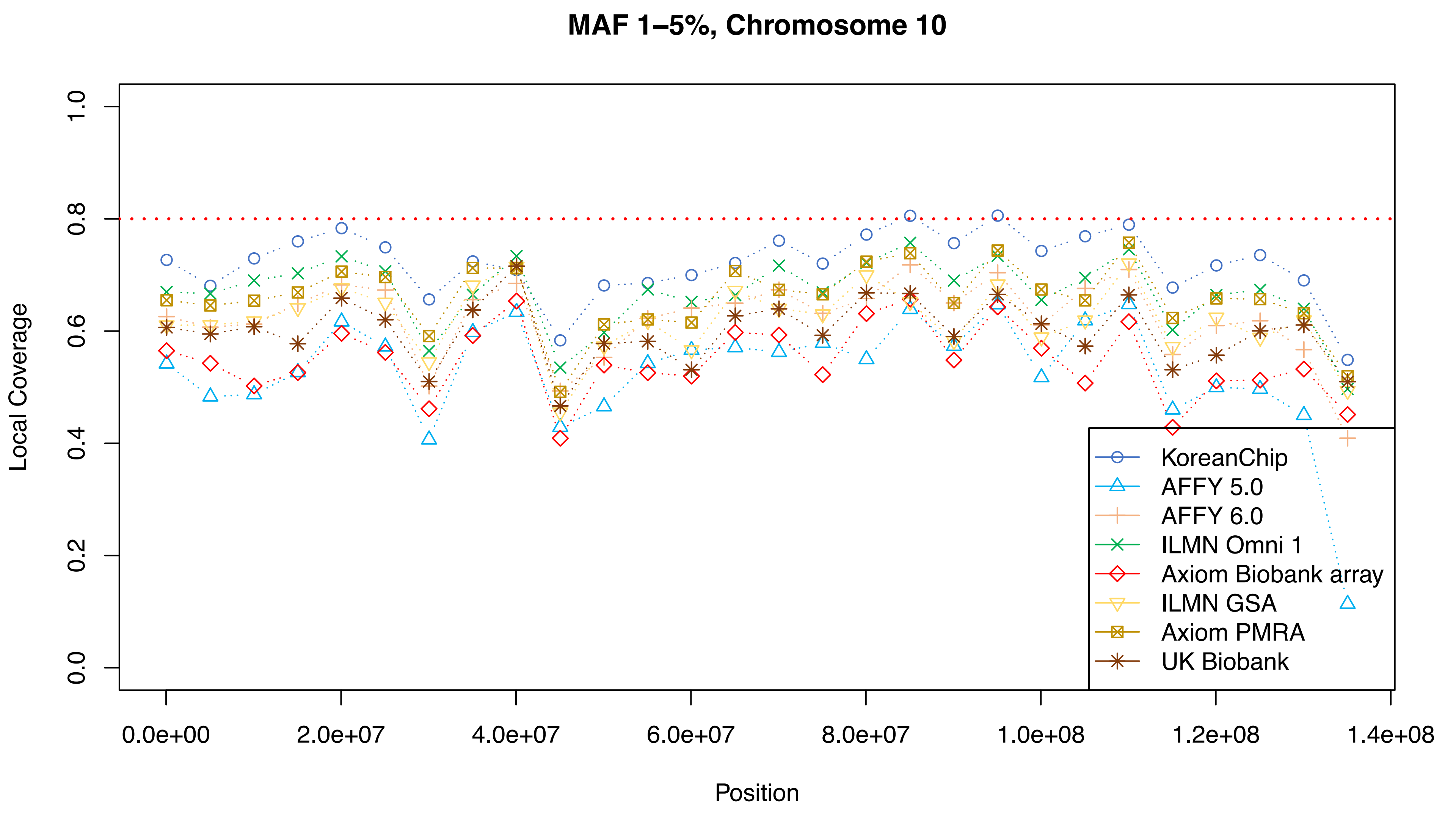

MAF 1–5%, Chromosome 10
Local Coverage
Position
0.0
0.2
0.4
0.6
0.8
1.0
0.0e+00
2.0e+07
4.0e+07
6.0e+07
8.0e+07
1.0e+08
1.2e+08
1.4e+08
KoreanChip
AFFY 5.0
AFFY 6.0
ILMN Omni 1
Axiom Biobank array
ILMN GSA
Axiom PMRA
UK Biobank

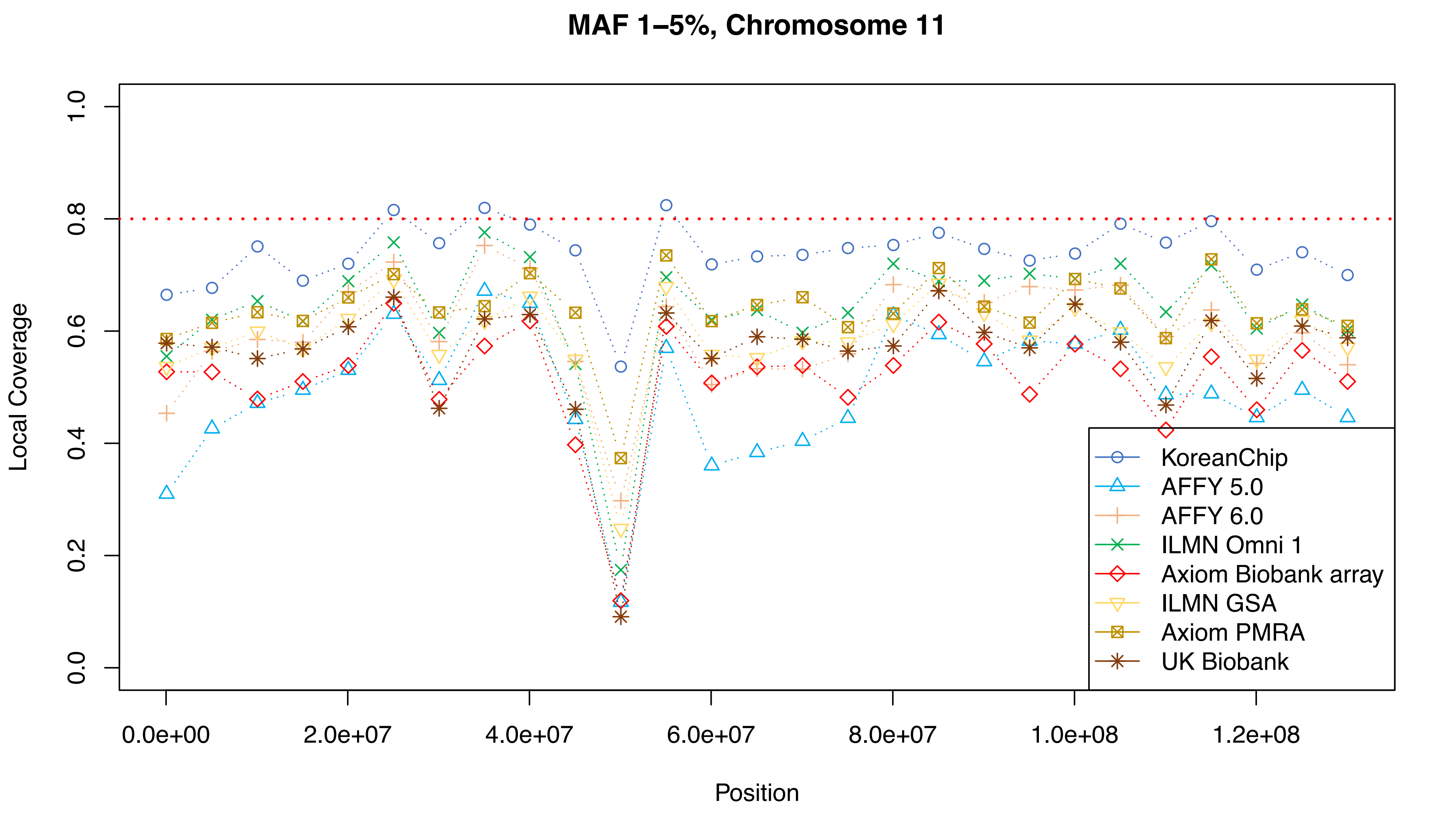

MAF 1–5%, Chromosome 11
Local Coverage
Position
0.0
0.2
0.4
0.6
0.8
1.0
0.0e+00
2.0e+07
4.0e+07
6.0e+07
8.0e+07
1.0e+08
1.2e+08
KoreanChip
AFFY 5.0
AFFY 6.0
ILMN Omni 1
Axiom Biobank array
ILMN GSA
Axiom PMRA
UK Biobank

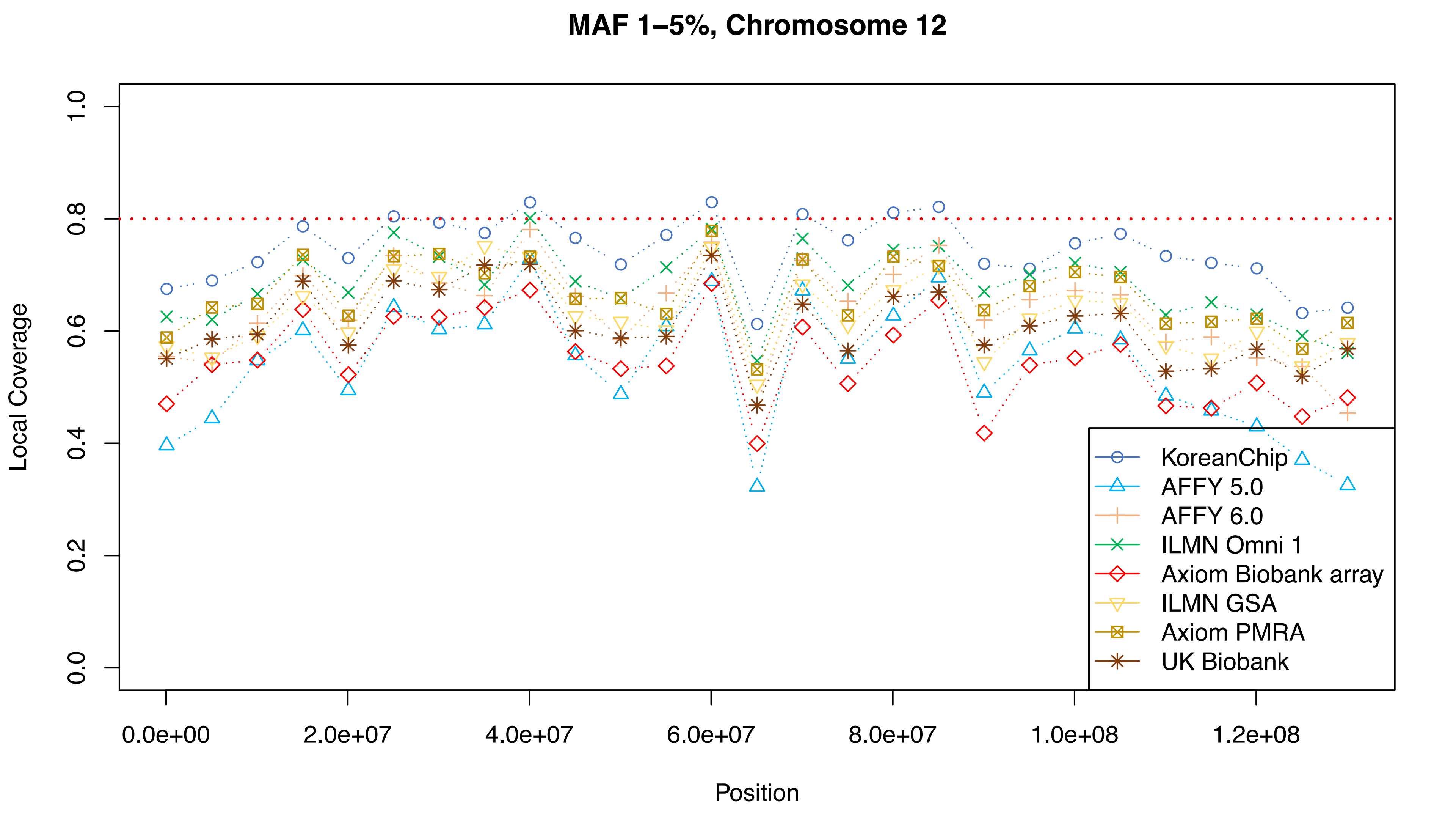
MAF 1–5%, Chromosome 12
Local Coverage
Position
0.0
0.2
0.4
0.6
0.8
1.0
0.0e+00
2.0e+07
4.0e+07
6.0e+07
8.0e+07
1.0e+08
1.2e+08
KoreanChip
AFFY 5.0
AFFY 6.0
ILMN Omni 1
Axiom Biobank array
ILMN GSA
Axiom PMRA
UK Biobank

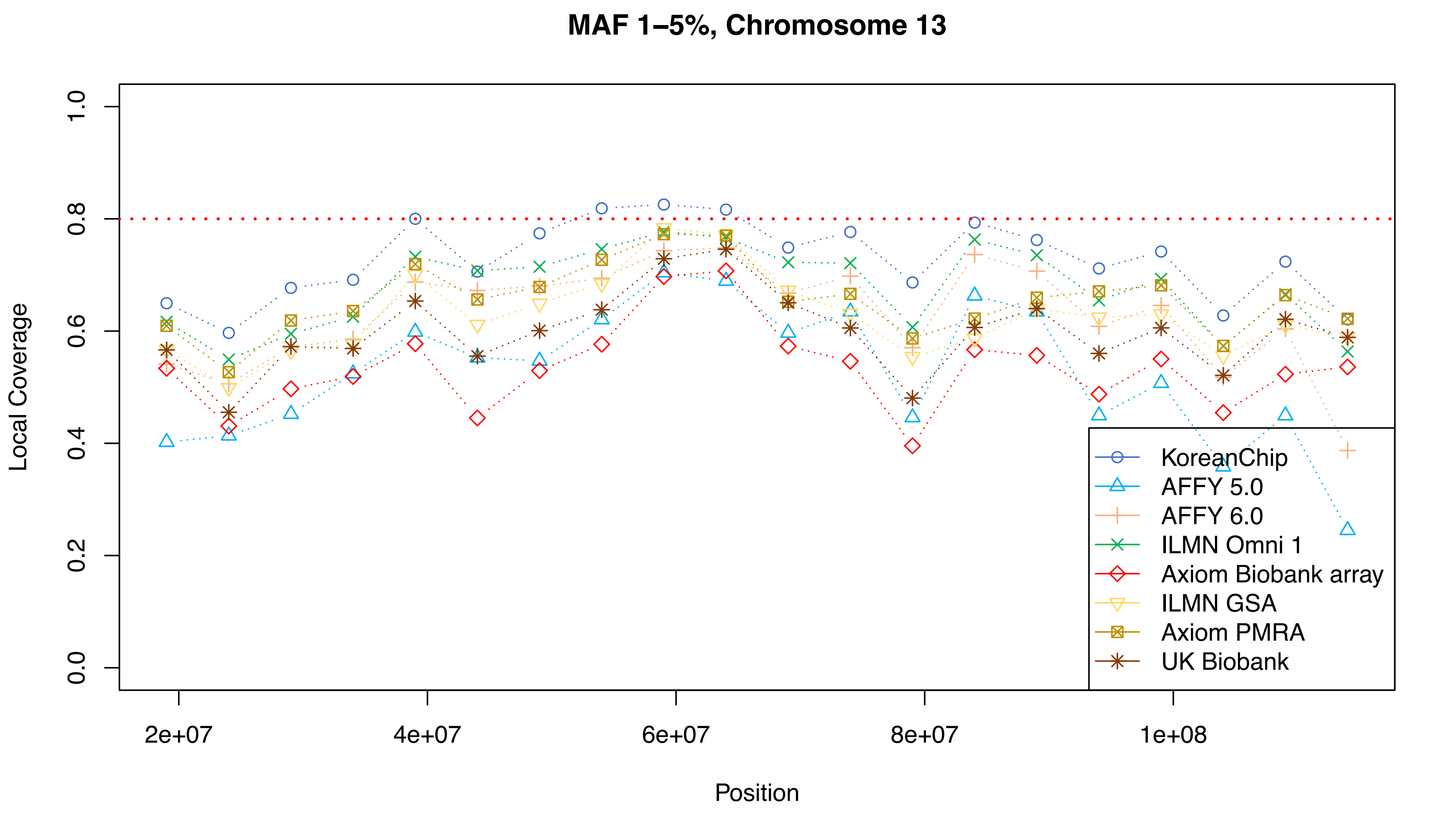
MAF 1–5%, Chromosome 13
Local Coverage
Position
0.0
0.2
0.4
0.6
0.8
1.0
2e+07
4e+07
6e+07
8e+07
1e+08
KoreanChip
AFFY 5.0
AFFY 6.0
ILMN Omni 1
Axiom Biobank array
ILMN GSA
Axiom PMRA
UK Biobank

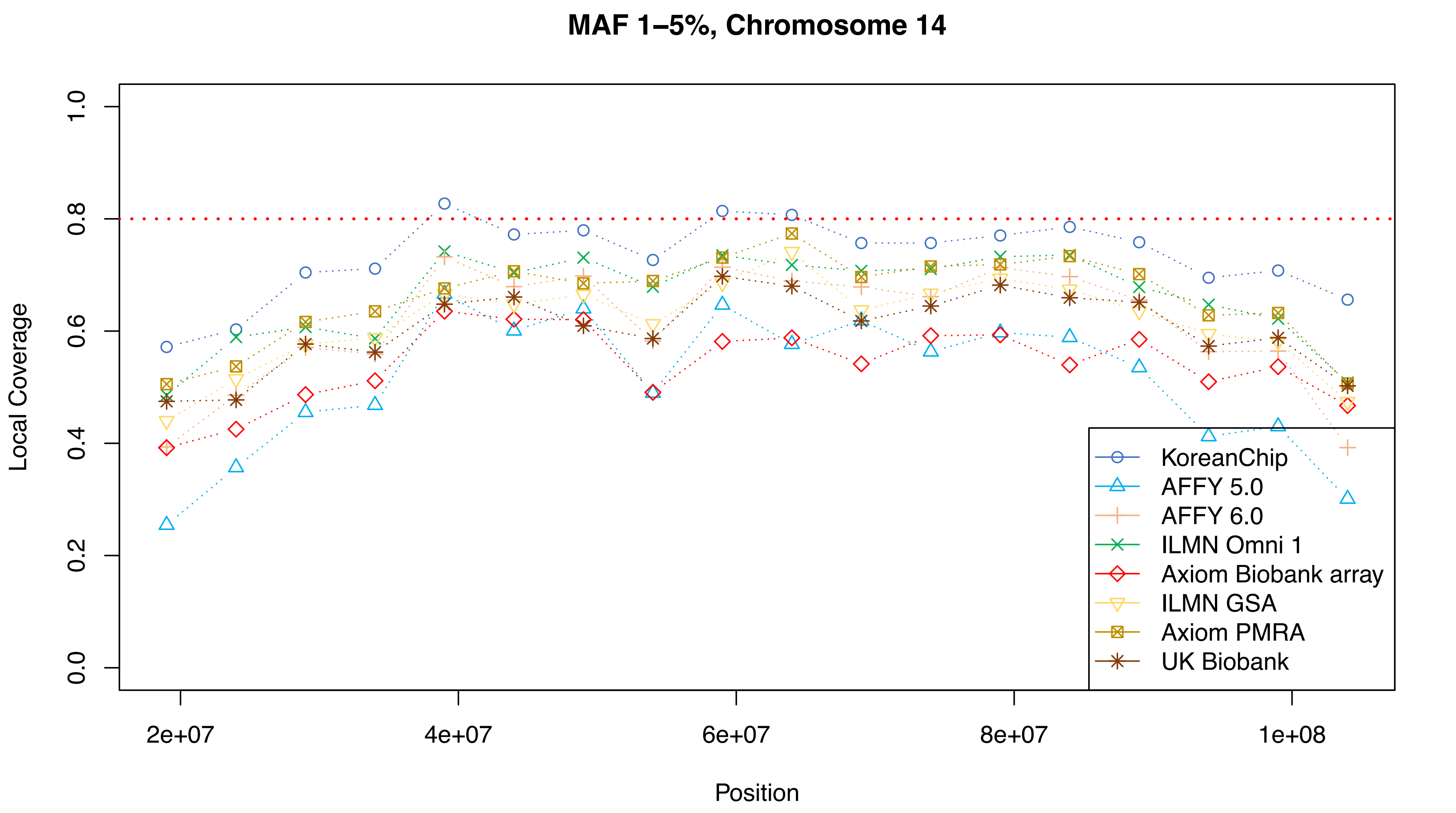

MAF 1–5%, Chromosome 14
Local Coverage
Position
0.0
0.2
0.4
0.6
0.8
1.0
2e+07
4e+07
6e+07
8e+07
1e+08
KoreanChip
AFFY 5.0
AFFY 6.0
ILMN Omni 1
Axiom Biobank array
ILMN GSA
Axiom PMRA
UK Biobank

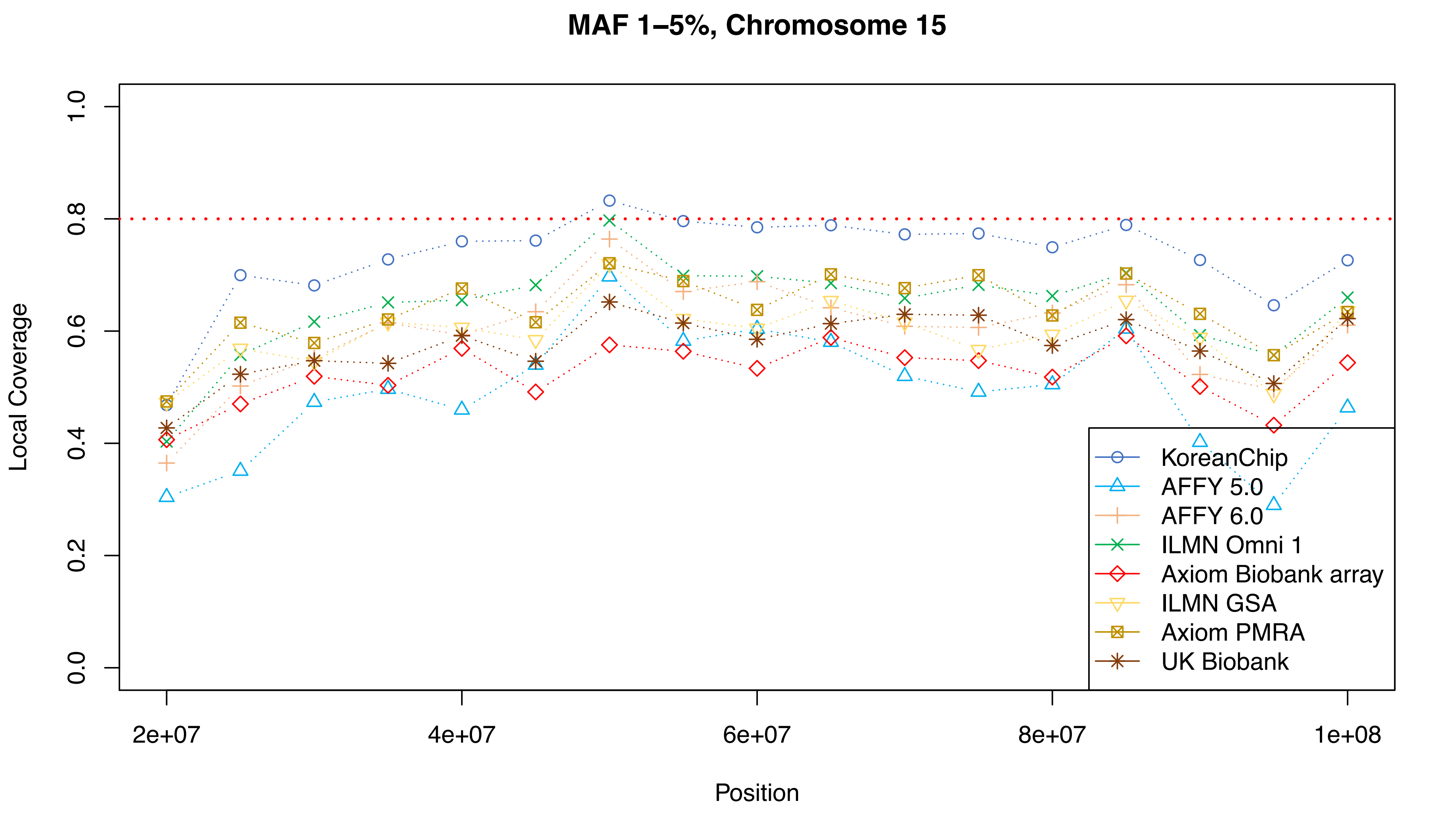

MAF 1–5%, Chromosome 15
Local Coverage
Position
0.0
0.2
0.4
0.6
0.8
1.0
2e+07
4e+07
6e+07
8e+07
1e+08
KoreanChip
AFFY 5.0
AFFY 6.0
ILMN Omni 1
Axiom Biobank array
ILMN GSA
Axiom PMRA
UK Biobank

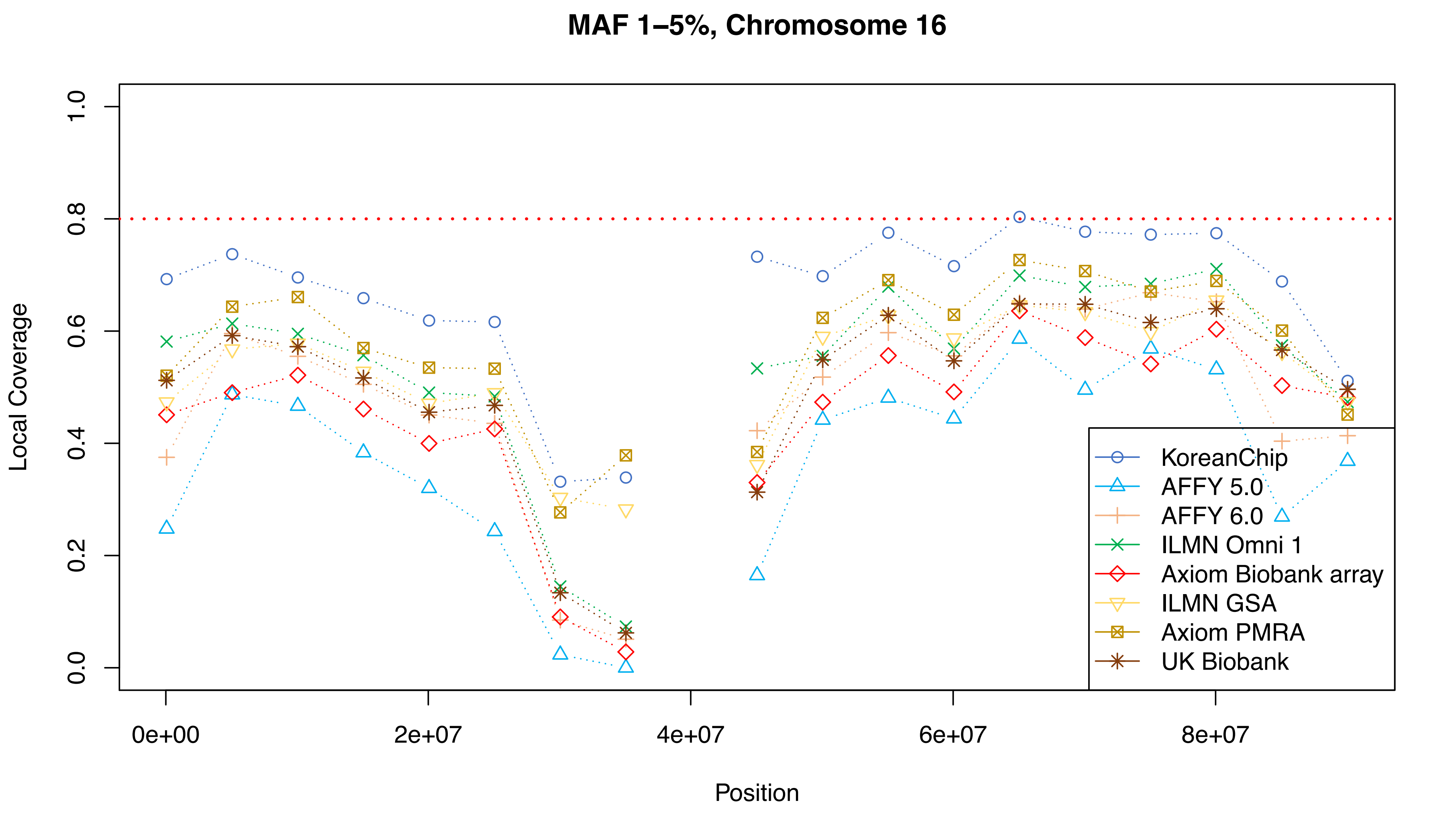
MAF 1–5%, Chromosome 16
Local Coverage
Position
0.0
0.2
0.4
0.6
0.8
1.0
0e+00
2e+07
4e+07
6e+07
8e+07
KoreanChip
AFFY 5.0
AFFY 6.0
ILMN Omni 1
Axiom Biobank array
ILMN GSA
Axiom PMRA
UK Biobank

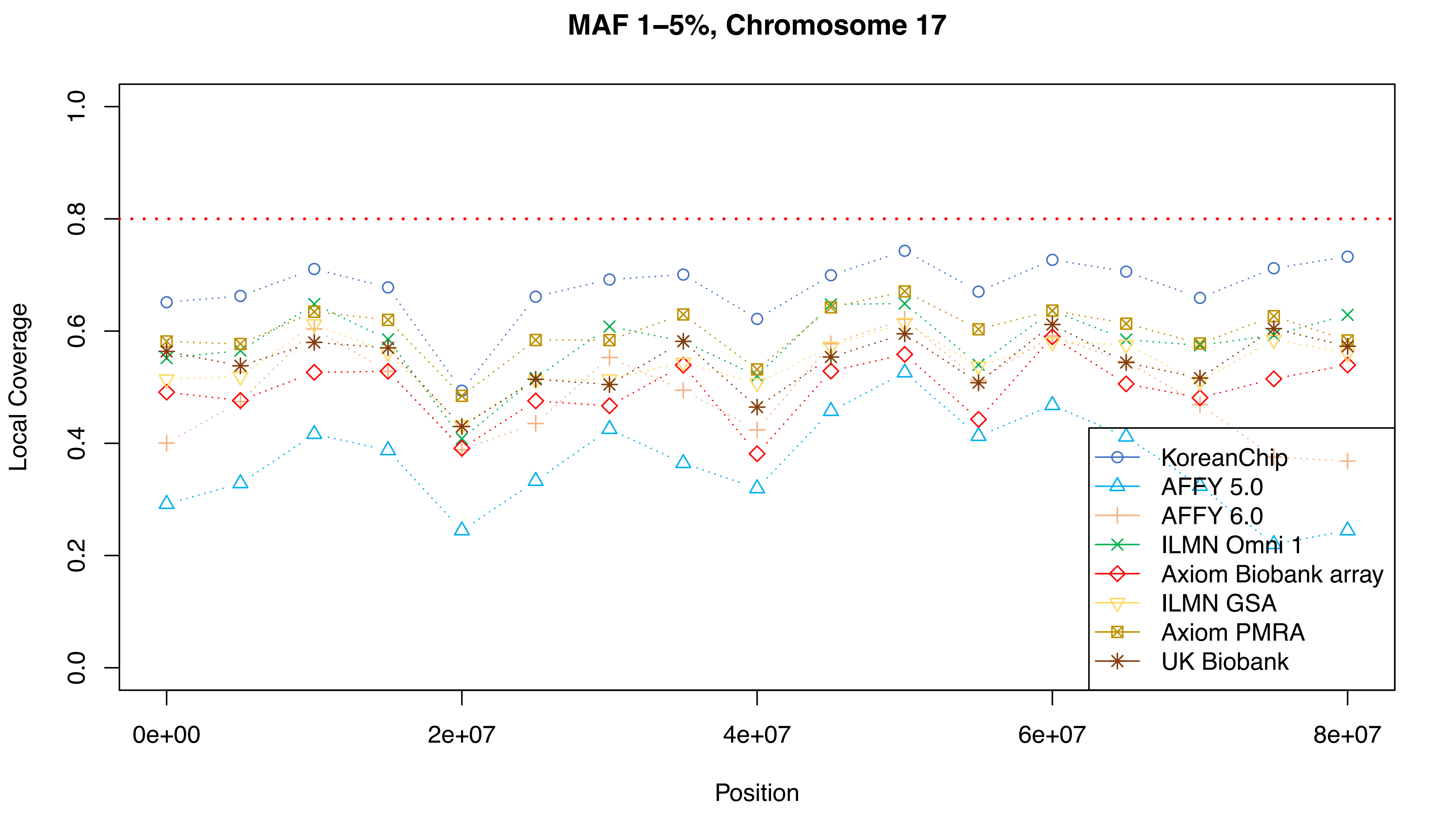
MAF 1–5%, Chromosome 17
Local Coverage
0.0
0.2
0.4
0.6
0.8
1.0
0e+00
2e+07
4e+07
6e+07
8e+07
Position
KoreanChip
AFFY 5.0
AFFY 6.0
ILMN Omni 1
Axiom Biobank array
ILMN GSA
Axiom PMRA
UK Biobank

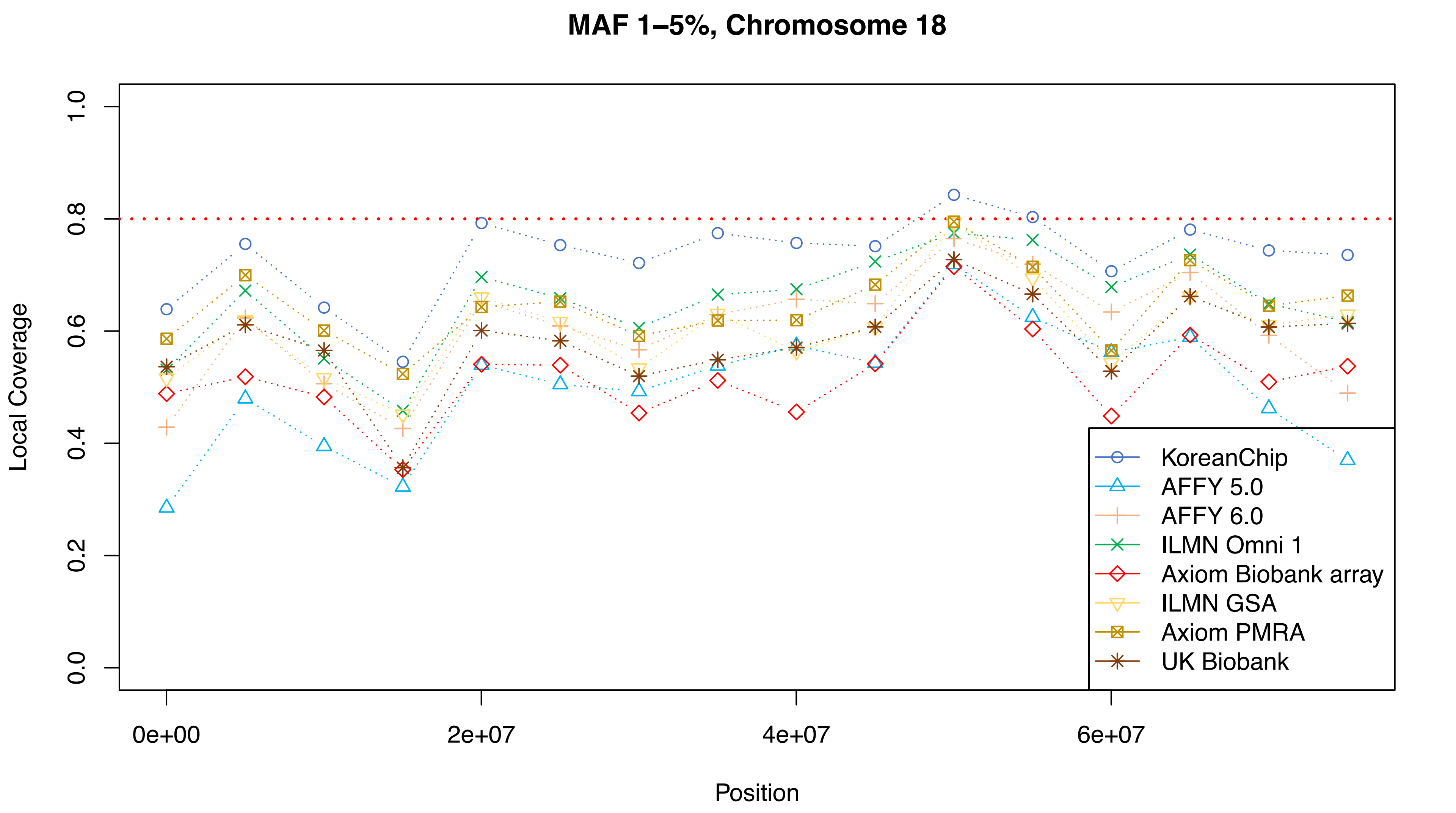
MAF 1–5%, Chromosome 18
Local Coverage
Position
0.0
0.2
0.4
0.6
0.8
1.0
0e+00
2e+07
4e+07
6e+07
KoreanChip
AFFY 5.0
AFFY 6.0
ILMN Omni 1
Axiom Biobank array
ILMN GSA
Axiom PMRA
UK Biobank

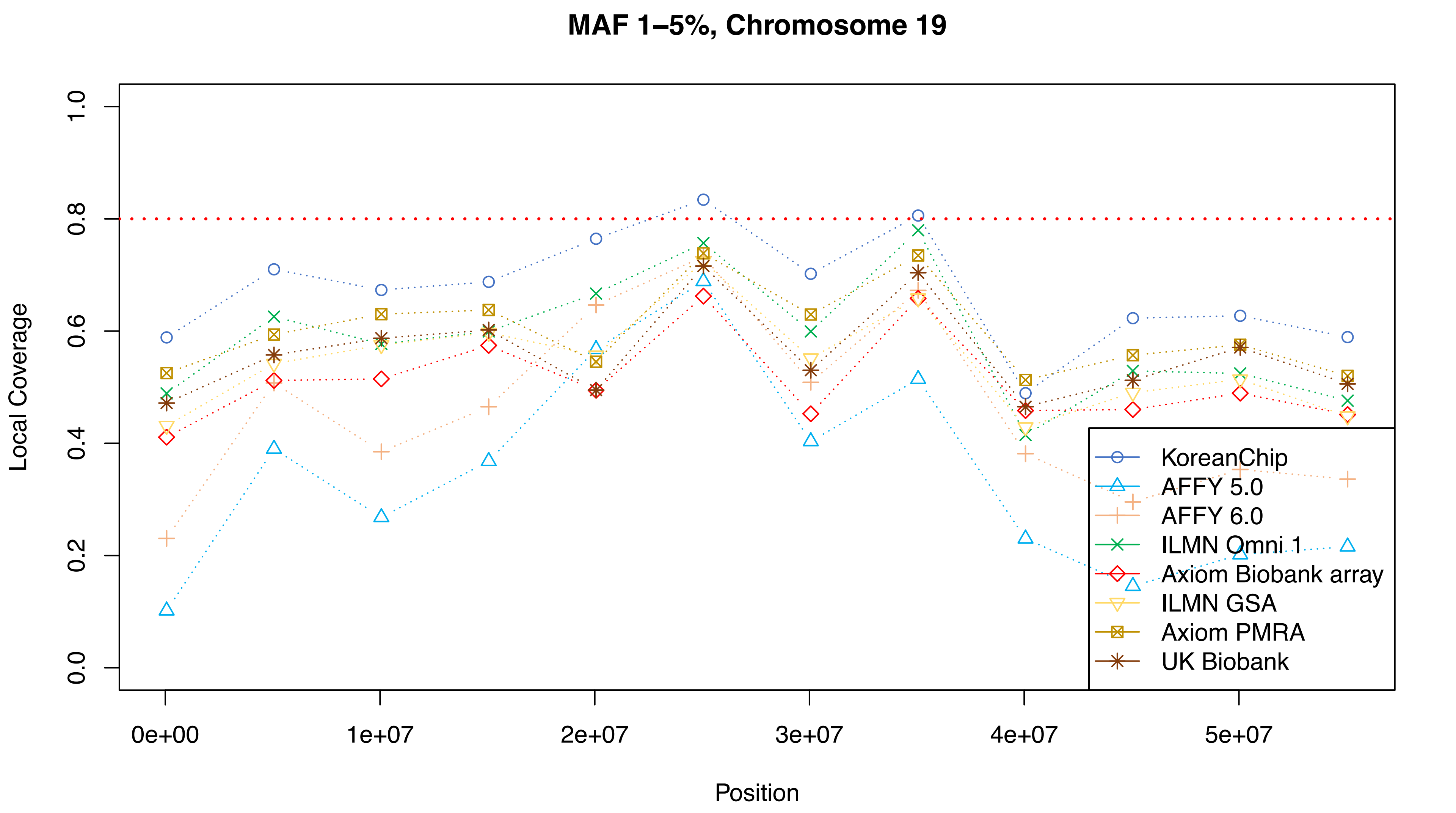
MAF 1–5%, Chromosome 19
Local Coverage
0.0
0.2
0.4
0.6
0.8
1.0
Position
0e+00
1e+07
2e+07
3e+07
4e+07
5e+07
KoreanChip
AFFY 5.0
AFFY 6.0
ILMN Omni 1
Axiom Biobank array
ILMN GSA
Axiom PMRA
UK Biobank

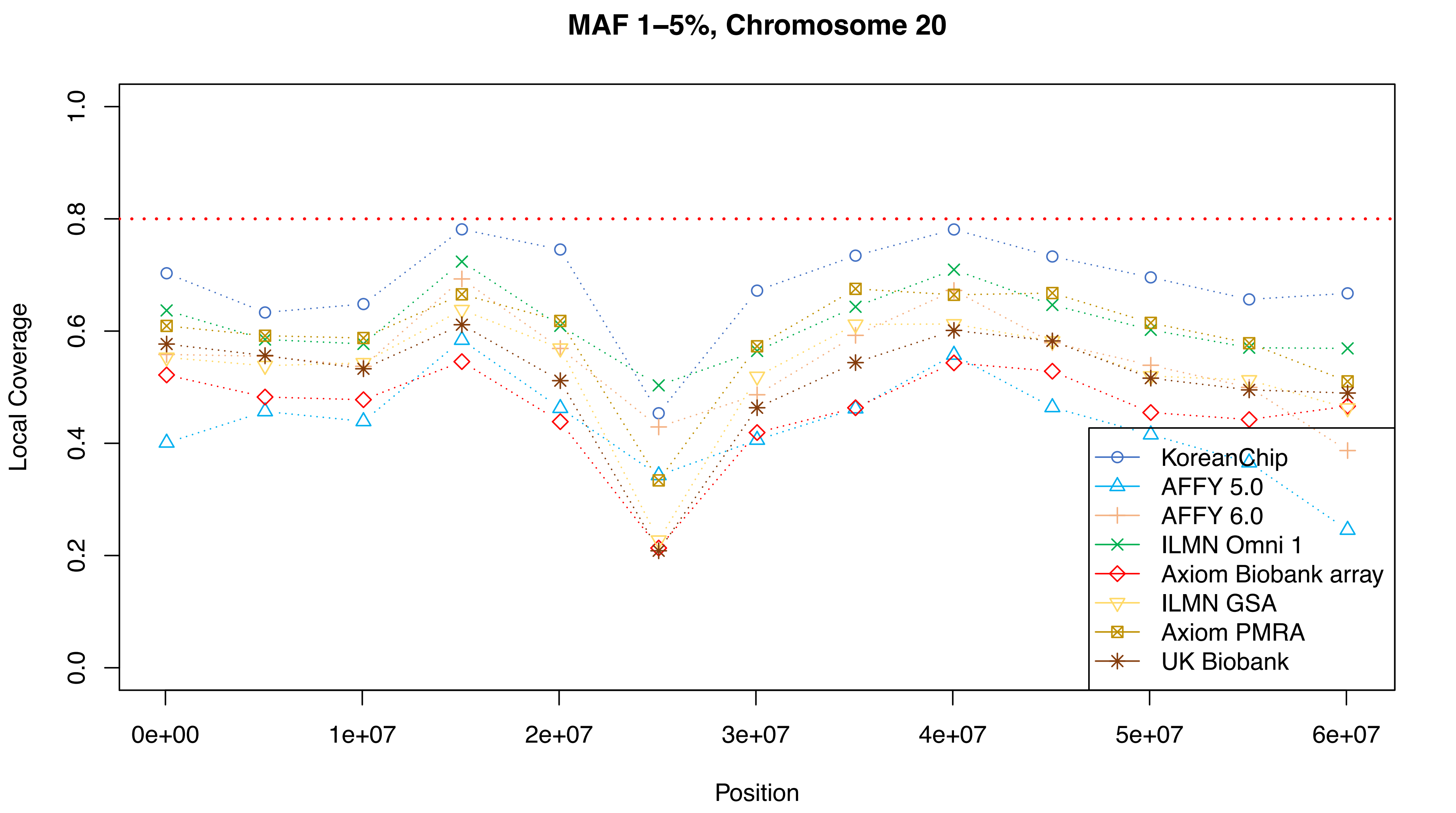
MAF 1–5%, Chromosome 20
Local Coverage
Position
0.0
0.2
0.4
0.6
0.8
1.0
0e+00
1e+07
2e+07
3e+07
4e+07
5e+07
6e+07
KoreanChip
AFFY 5.0
AFFY 6.0
ILMN Omni 1
Axiom Biobank array
ILMN GSA
Axiom PMRA
UK Biobank

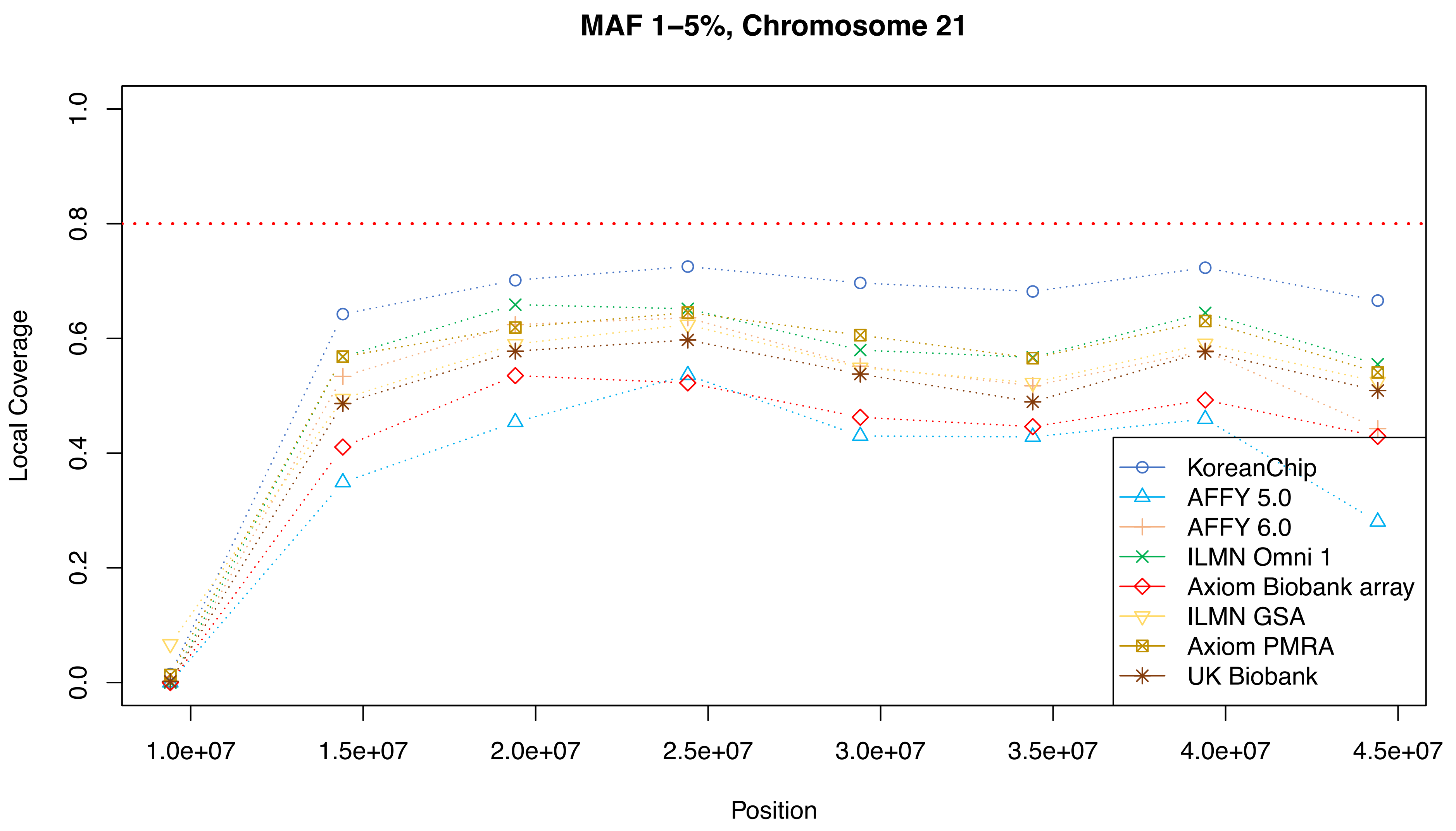
MAF 1–5%, Chromosome 21
Local Coverage
0.0
0.2
0.4
0.6
0.8
1.0
Position
1.0e+07
1.5e+07
2.0e+07
2.5e+07
3.0e+07
3.5e+07
4.0e+07
4.5e+07
KoreanChip
AFFY 5.0
AFFY 6.0
ILMN Omni 1
Axiom Biobank array
ILMN GSA
Axiom PMRA
UK Biobank

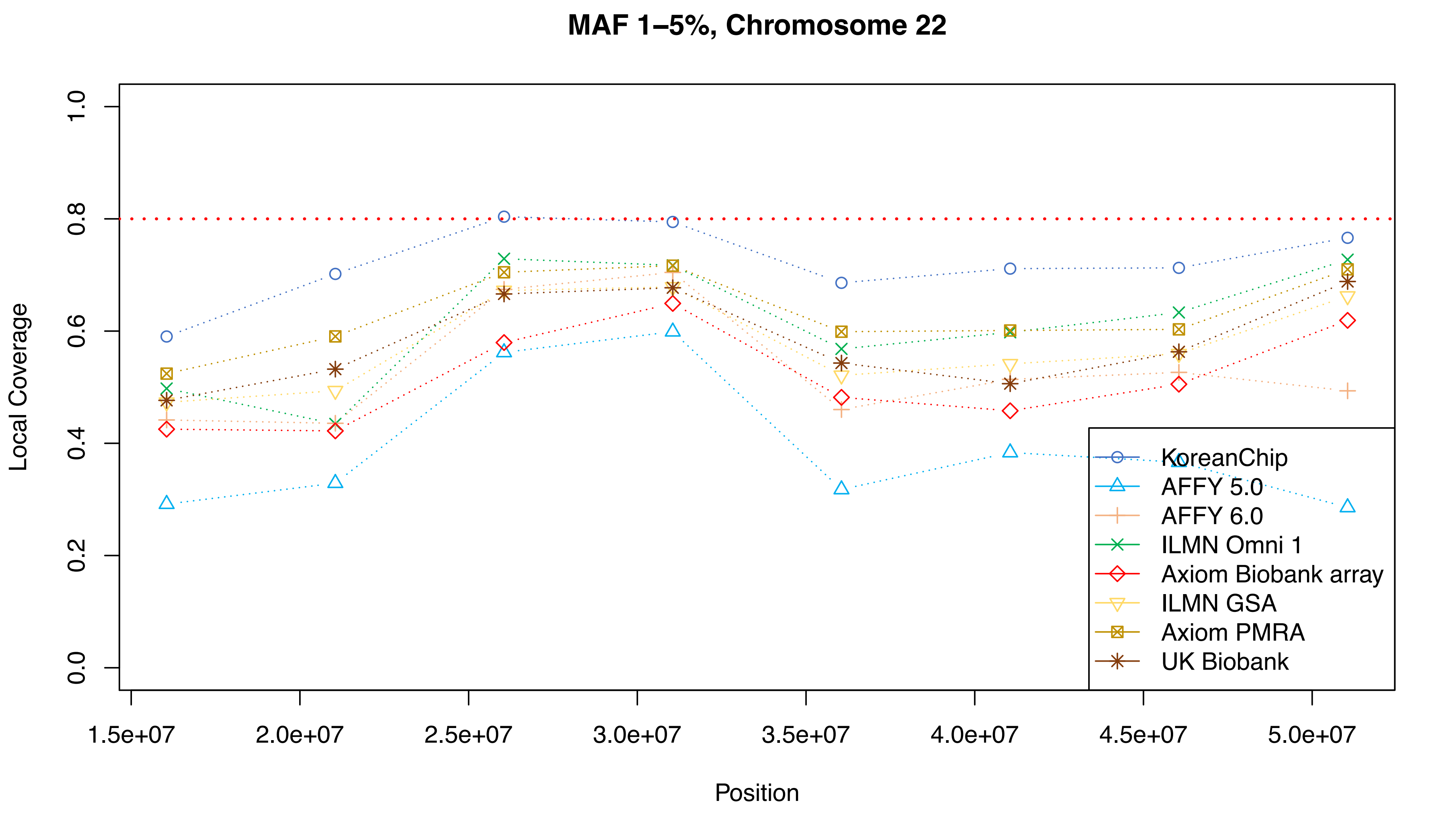
MAF 1–5%, Chromosome 22
Local Coverage
Position
0.0
0.2
0.4
0.6
0.8
1.0
1.5e+07
2.0e+07
2.5e+07
3.0e+07
3.5e+07
4.0e+07
4.5e+07
5.0e+07
KoreanChip
AFFY 5.0
AFFY 6.0
ILMN Omni 1
Axiom Biobank array
ILMN GSA
Axiom PMRA
UK Biobank

Fig. S4. Imputation quality test. Example of HDL. (A) Comparison of P-values by imputation quality score. (B) Comparison of info score between KoreanChip and Affymetrix 5.0. Overall, KoreanChip showed higher imputation quality and stronger statistical significance.

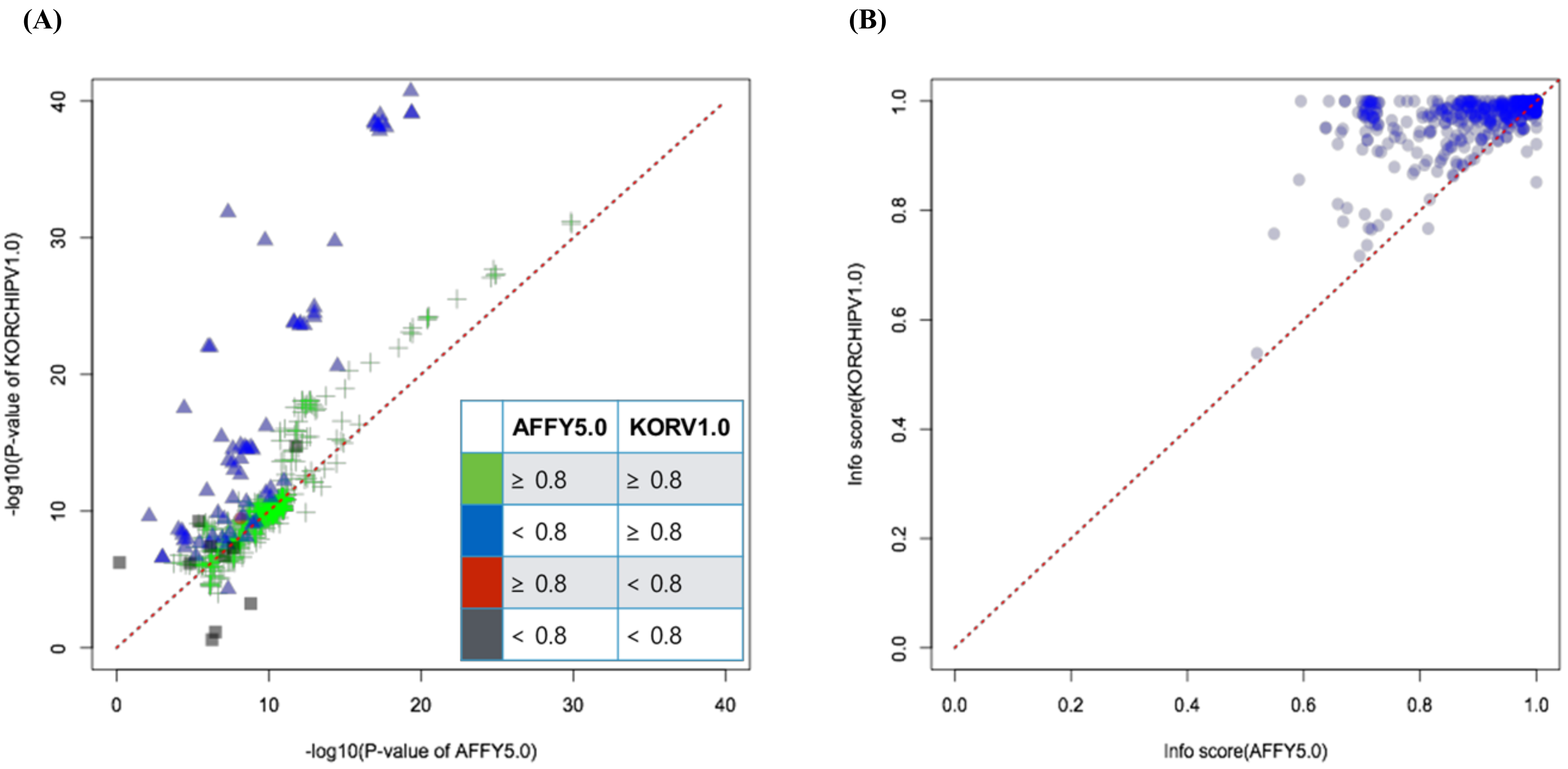

Fig. S5. Side-by-side Manhattan plots of each trait between two GWASs (results of previous study with Affymetrix 5.0 and those of KoreanChip). The pattern of associations in KoreanChip was very similar to that of previous study.

ALT

Affymetrix 5.0

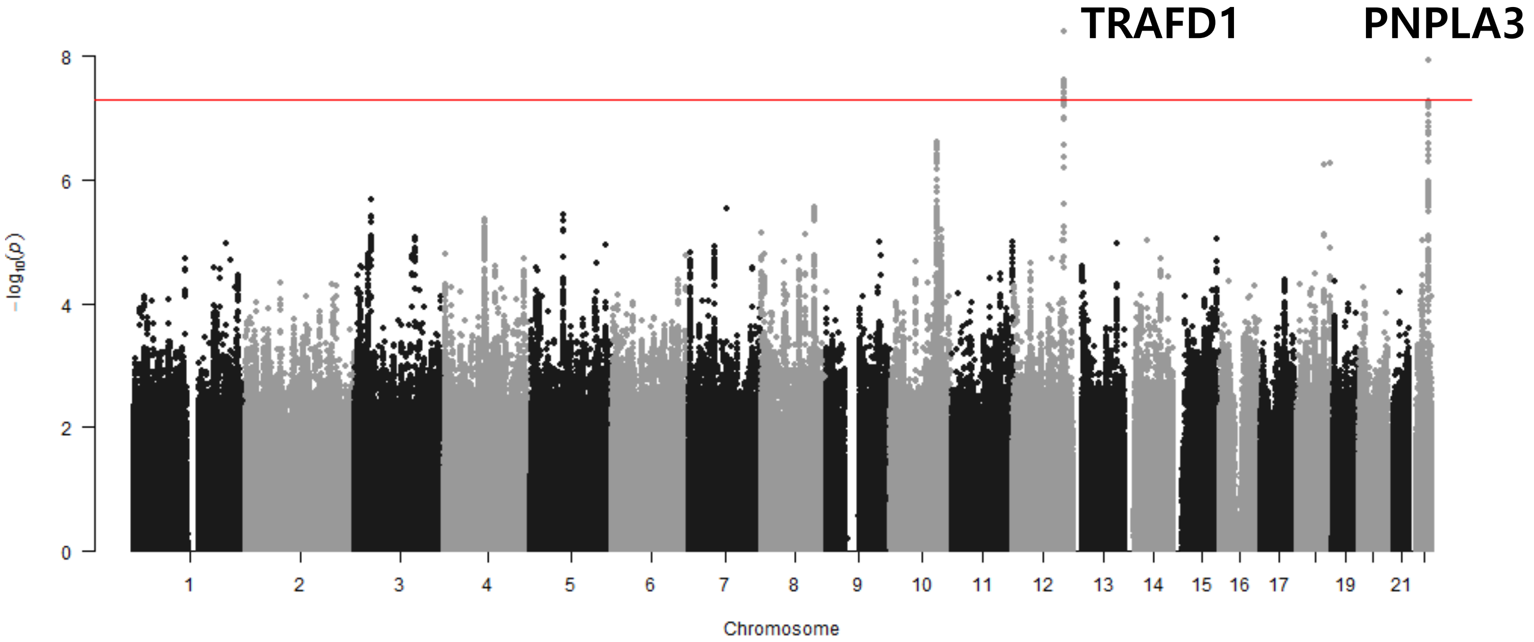

KoreanChip

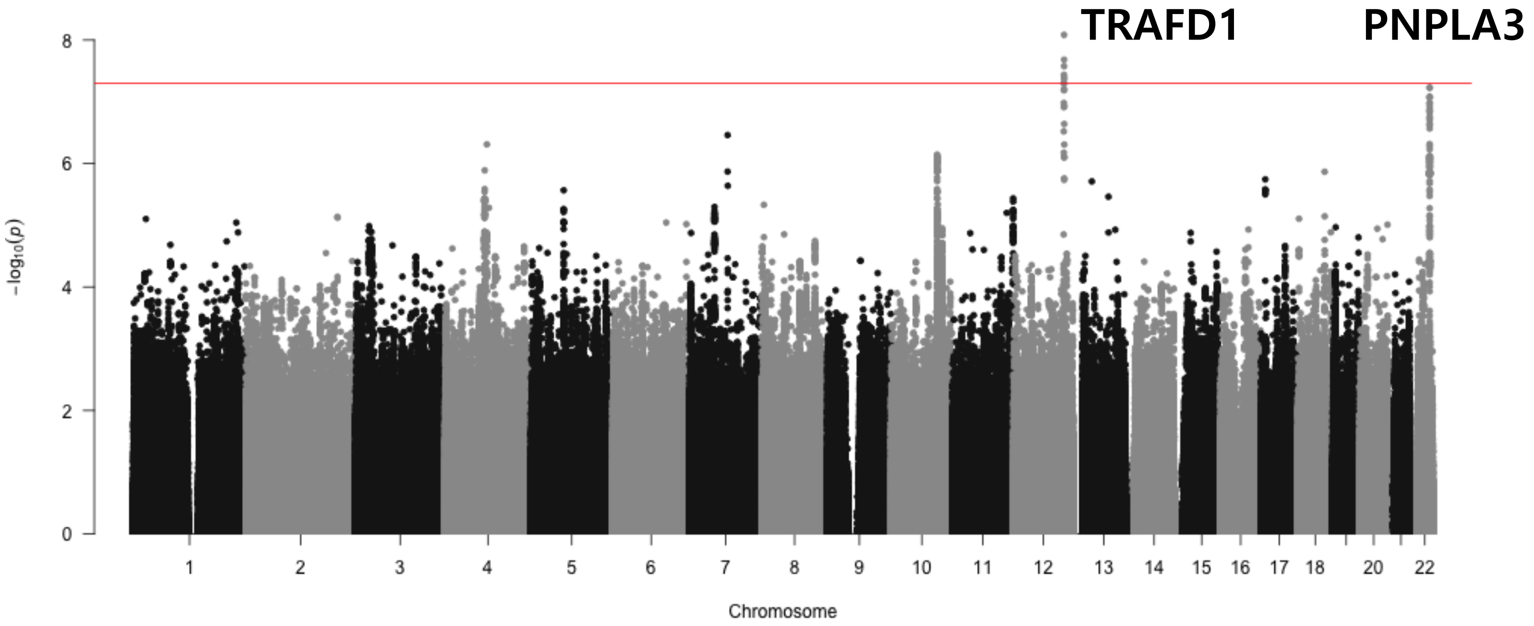

AST

Affymetrix 5.0

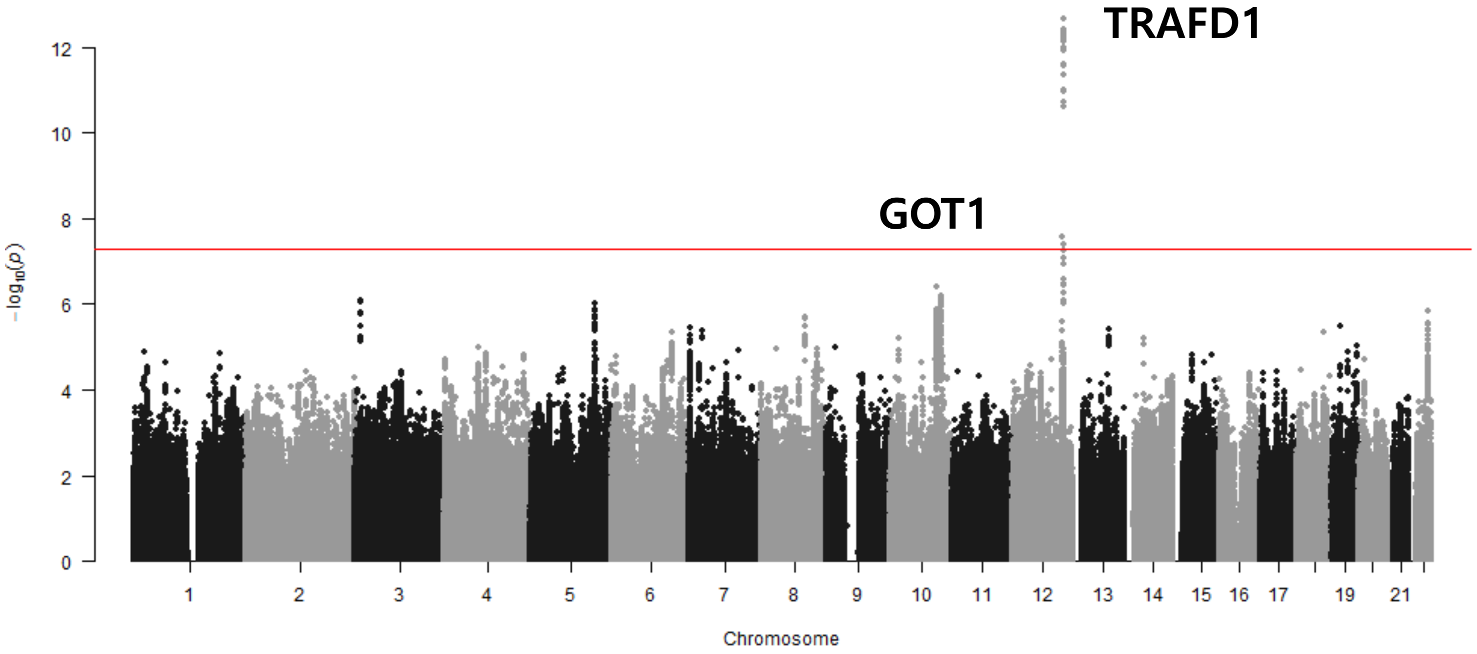

KoreanChip

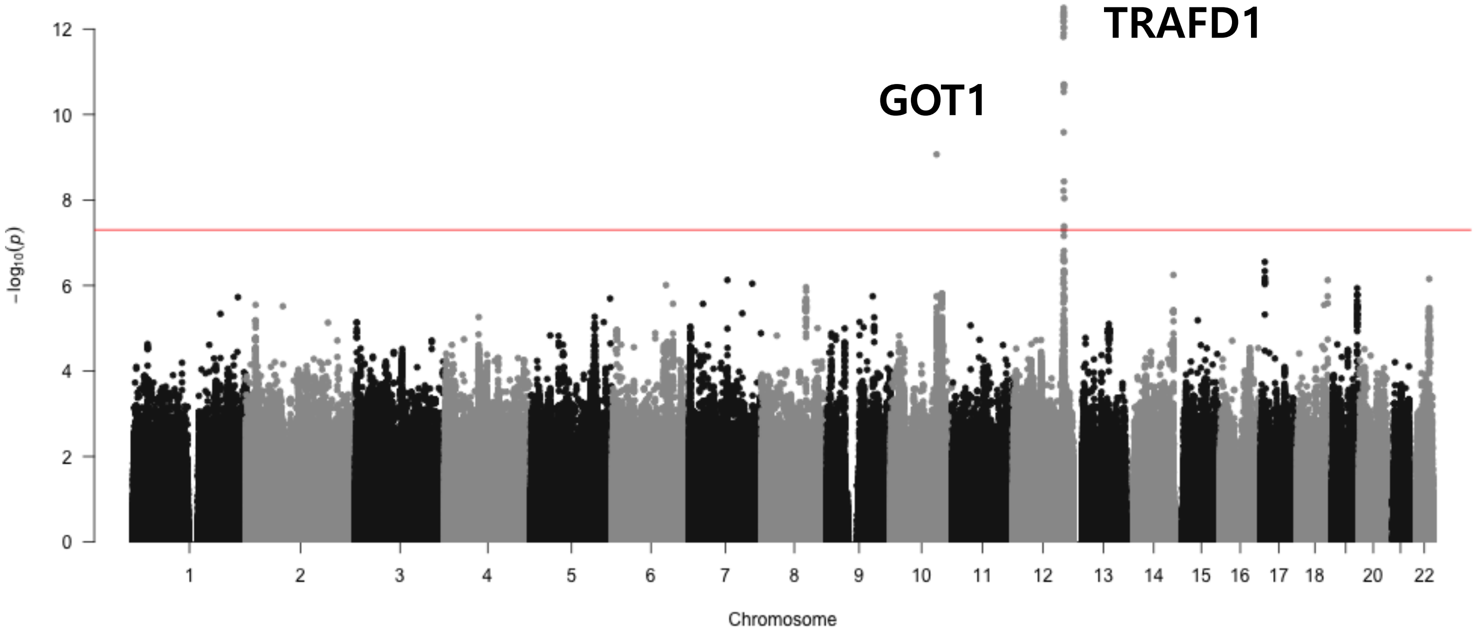

HDL

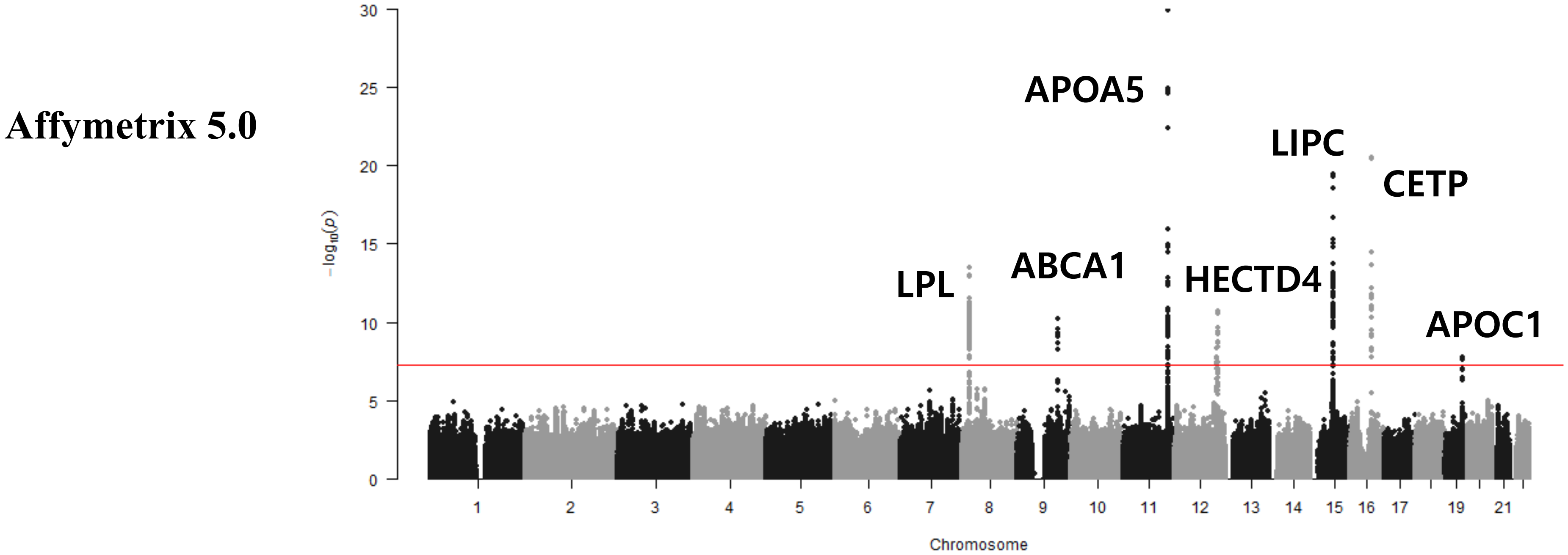
Affymetrix 5.0
APOA5
LIPC
CETP
LPL
ABCA1
HECTD4
APOC1
-log10(p)
Chromosome

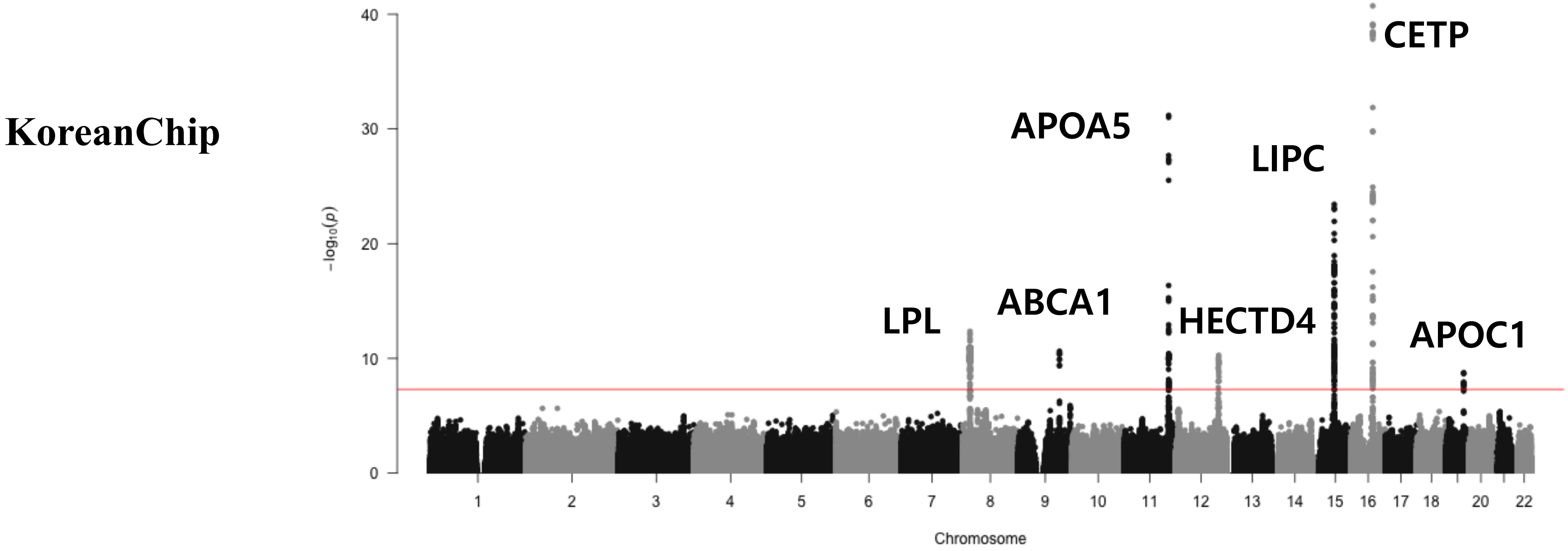
KoreanChip
CETP
APOA5
LIPC
LPL
ABCA1
HECTD4
APOC1
-log10(p)
Chromosome

LDL

Affymetrix 5.0

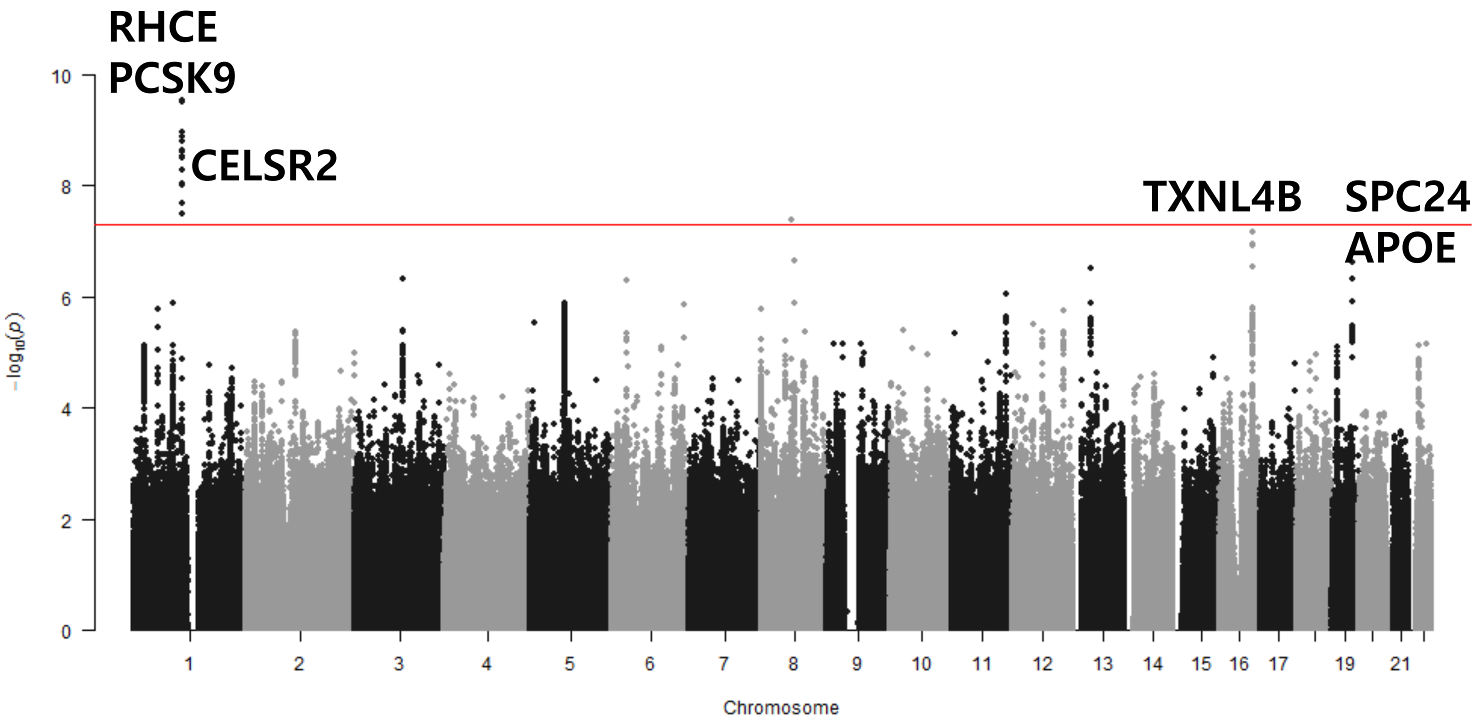

KoreanChip

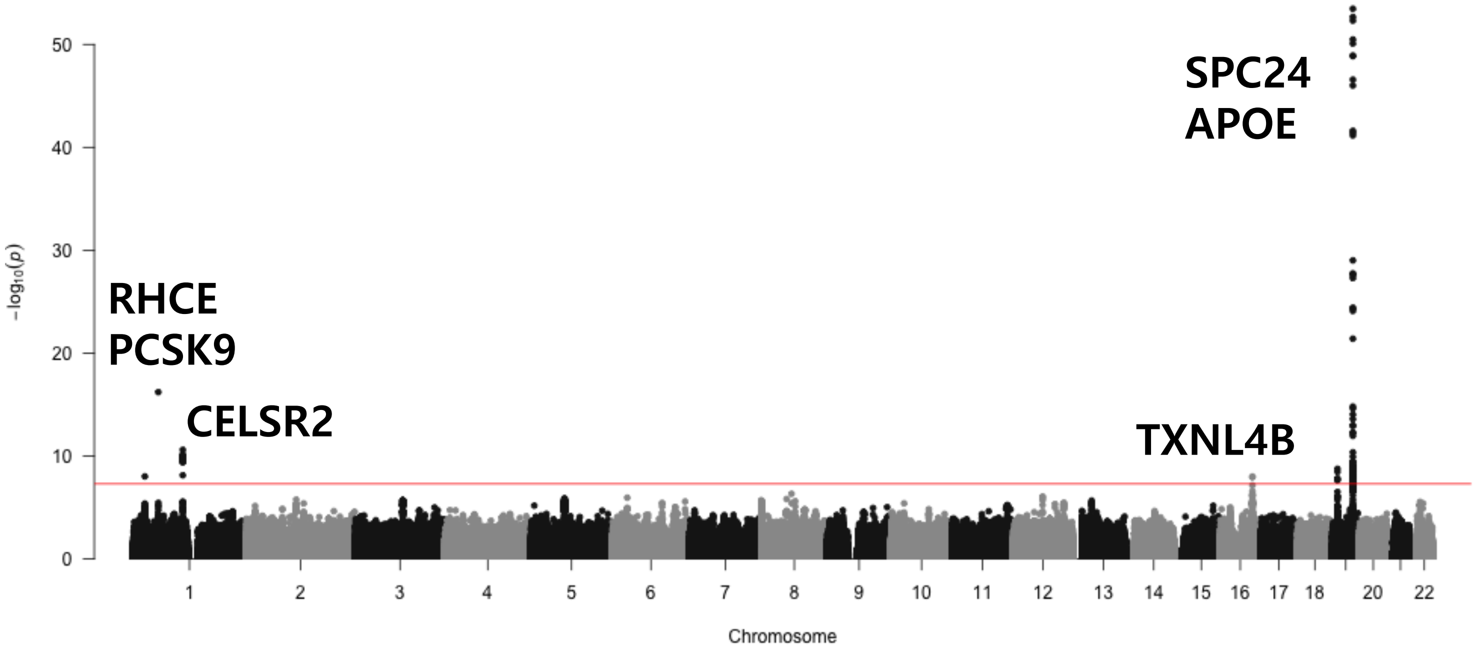

TG

Affymetrix 5.0

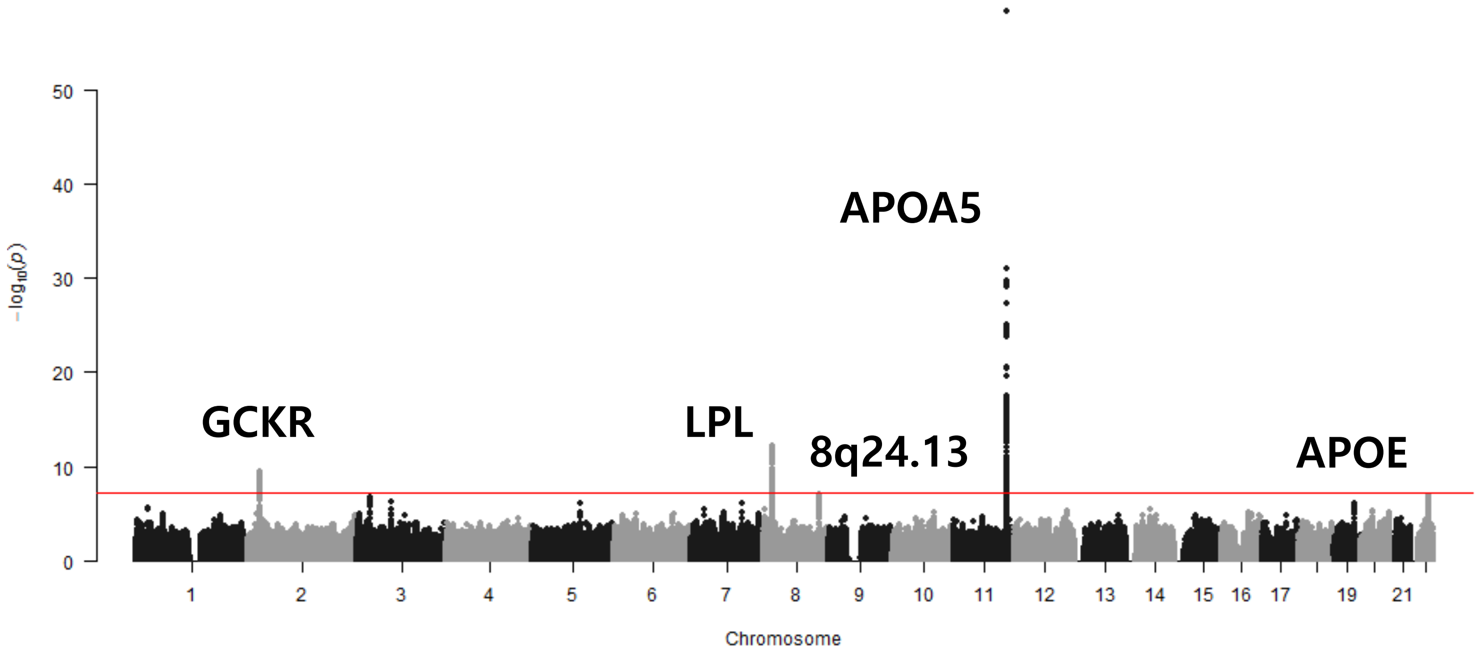

KoreanChip

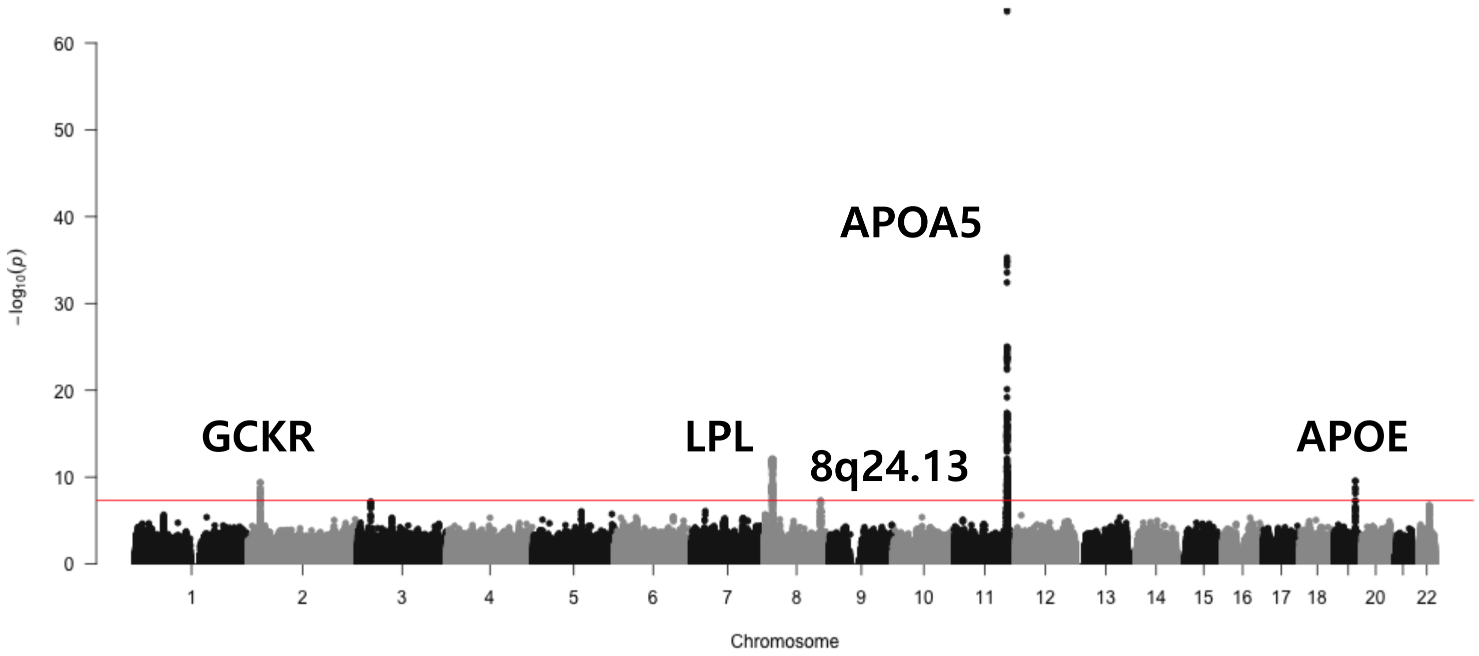

TCHL

Affymetrix 5.0

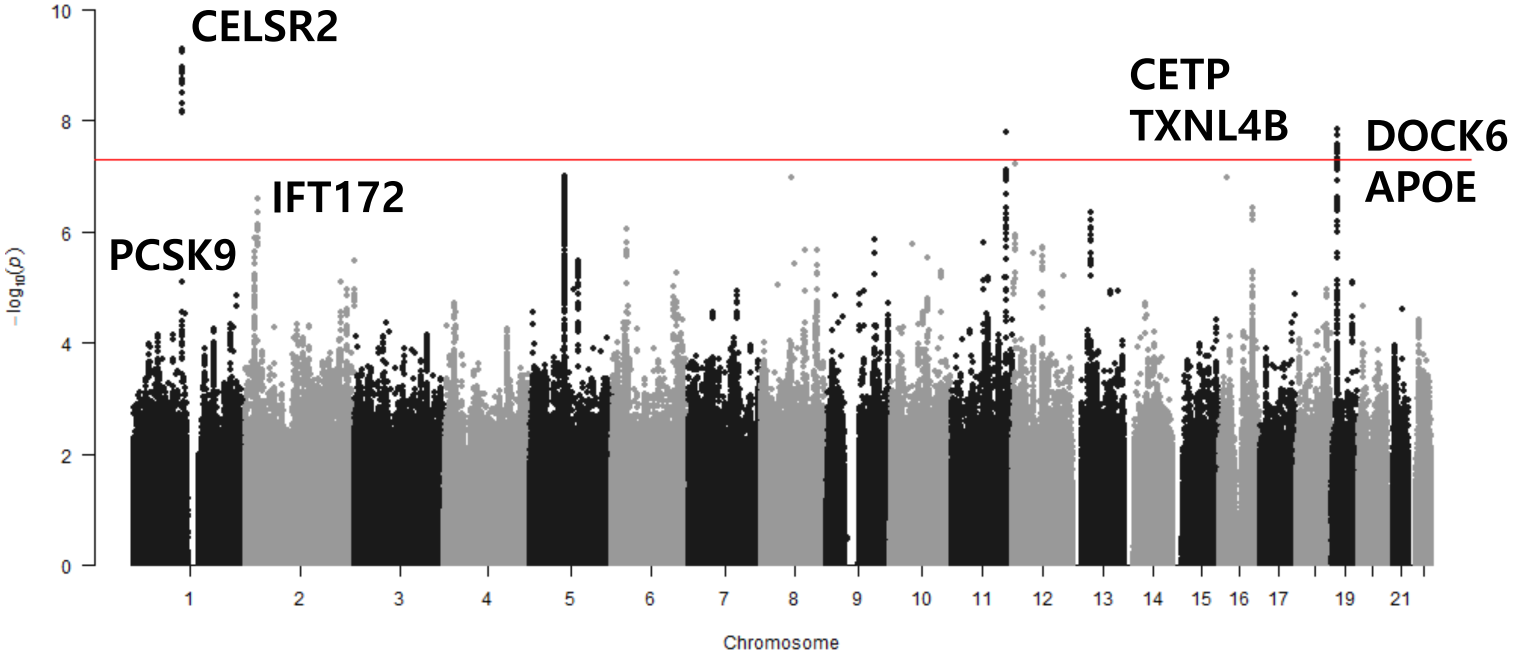

KoreanChip

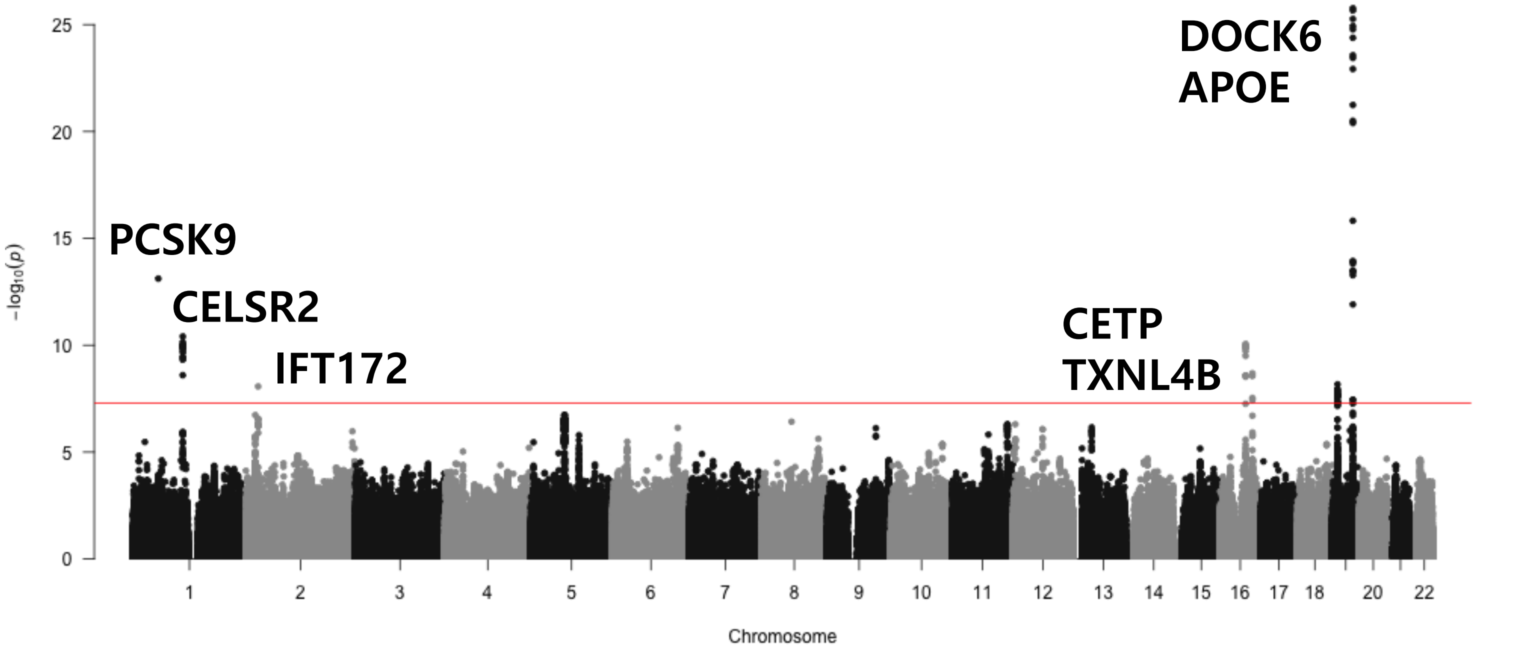

Table S1. Comparison of accuracy between KoreanChip and other platforms

Platform	Number of overlapping markers with KoreanChip		Accuracy (%)	
	Subject	Marker	Overall	Hetero
Affymetrix Genome-wide human SNP array 5.0	6,949	41,246	99.8	99.5
Illumina HumanExome BeadChip v1.1	5,793	34,683	99.9	99.7
Exome sequencing (Illumina Hiseq 2000)	155	90,020	99.8	99.7

Accuracy: # of True genotypes / # of Total genotypes

Overall: Overall accuracy, Hetero: Accuracy of heterozygotes

Table S2. Content comparison (commonly shared markers between KoreanChip and existing arrays).

Platform (N= # of marker)	# of shared markers (%)*		
	AFFY5.0	AFFY6.0	ILLU Omni 1M
KoreanChip (n=833,535)	47,846 (5.7%)	90,057 (10.8%)	123,761 (14.8%)
AFFY5.0 (n=500,568)	-	482,398 (96.4%)	140,046 (27.98%)
AFFY6.0 (n=906,600)	-	-	271,989 (30.0%)

* of shared markers was calculated as (# of shared markers / # of total markers (from platform in the first column of the table))×100. Markers were regarded as shared if two platforms had variants with identical information (chromosome, position, reference allele, and alternative allele).

AFFY5.0, Affymetrix Genome-wide Human SNP array 5.0; AFFY6.0, Affymetrix Genome-wide Human SNP array 6.0; ILLU Omni 1M, Illumina HumanOmni1-Quad BeadChip

Table S3. Content comparison (commonly shared markers between KoreanChip and next-generation arrays)

Platform (n = # of marker)	# of shared markers (%)*				
	Axiom Biobank	UK Biobank	ILMN Exome	PMRA	GSA
KoreanChip (n=833,535)	219,690 (26.4%)	238,929 (28.7%)	42,807 (5.1%)	275,312 (33.0%)	97,686 (11.7%)
Axiom Biobank (n=628,679)	-	398,587 (63.4%)	229,317 (36.5%)	244,305 (38.9%)	137,876 (21.9%)
Axiom UK Biobank (n=830,114)	-	-	82,225 (9.9%)	286,215 (34.5%)	288,777 (34.8%)
ILMN Exome (n=242,901)	-	-	-	34,348 (14.1%)	51,927 (21.4%)
PMRA (n=920,636)	-	-	-	-	124,994 (13.6%)

* % of shared markers was calculated as (# of shared markers / # of total markers (from platform in the first column of the table))×100. Markers were regarded as shared if two platforms had variants with identical information (chromosome, position, reference allele, and alternative allele)

ILMN Exome, Illumina HumanExome BeadChip; PMRA, Axiom Precision Medicine Research Array; GSA, Global Screening Array.

Table S4. Comparison of GWAS results with known loci. Association results of the same markers from the same individuals genotyped with Affymetrix 5.0 (Kim et al. Nat. Genet. 2011, 43:990-5) were used. Association results from the two different platforms were similar. TCHL was not shown in this table due to absence of TCHL information in the report of Kim et al.

Trait	RS ID	CHR	Candidate gene	P-value (AFFY5.0)	P-value (KoreanChip)
HDL	rs16940212	15		6.21E-12	6.27E-12
	rs10503669	8	LPL	1.39E-11	1.21E-11
	rs11216126	11		8.44E-11	8.59E-11
	rs12686004	9	ABCA1	6.00E-10	3.83E-10
	rs2074356	12	C12orf51	2.10E-09	2.08E-09
	rs12708980	16	CETP	5.48E-09	6.97E-10
	rs12229654	12	MYL2	1.60E-08	9.81E-09
	rs2072134	12	OAS3	1.42E-06	1.42E-06
	rs519113	19	PVRL2-TOMM40-APOE	6.80E-04	6.51E-04
LDL	rs599839	1	CELSR2-PSRC1-SORT1	1.02E-08	7.39E-09
	rs12654264	5	MGGCR	1.31E-06	1.42E-06
	rs2738446	19	LDLR	1.20E-05	2.28E-05
	rs651007	9	ABO	1.51E-04	1.69E-04
TG	rs603446	11	ZNF259	2.07E-14	2.32E-14
	rs10503669	8	LPL	9.78E-13	9.93E-13
	rs780092	2	GCKR	1.46E-09	1.64E-09
	rs2001945	8		1.04E-05	1.01E-05
	rs2286276	7	TBL2-MLXIPL	1.02E-04	1.02E-04
ALT	rs12483959	22	PNPLA3	5.32E-08	5.89E-08
	rs11066280	12	C12orf51	1.02E-07	1.17E-07
AST	rs11066280	12	C12orf51	1.88E-11	1.99E-11

CHR, chromosome; AFFY5.0, Affymetrix Genome-wide Human SNP array 5.0; TG, triglyceride; LDL, Low-density lipoprotein cholesterol; HDL, high-density lipoprotein cholesterol; TCHL, Total cholesterol; AST, Aspartate aminotransferase; ALT, alanine aminotransferase.

Table S5. Replication of known associations at known loci

Gene	rs ID	Position	Effective allele /Other	Trait	EAF				Beta	Beta(SE)	P-value	Reference (PMID)
						gnomAD						
					KOR	EA	EUR	AFR				
Known association at known loci (common variants)												
RHCE	rs28695210	chr1:25749269	A/G	LDL	12.9	8.9	7.6	1.7	0.0481	0.0084	9.53E-09	25961943
CELSR2	rs12740374	chr1:109817590	T/G	LDL	5.9	6.3	21.9	25.4	-0.0714	0.0107	2.50E-11	19060906
				TCHL					-8.3697	1.2645	3.89E-11	19060911
**IFT172**	**rs151269177**	**chr2:27674931**	**A/AAAC**	**TCHL**	**29.8**	**-**	**-**	**-**	**-3.9639**	**0.6875**	**8.49E-09**	**27286809**
**GCKR**	**rs57634090**	**chr2:27731739**	**CTT/C**	**TG**	**32.3**	**-**	**-**	**-**	**-0.0565**	**0.0090**	**4.01E-10**	**28334899**
LPL	rs78963197	chr8:19836646	C/T	TG	12.4	10.2	9.1	4.7	-0.0905	0.0126	7.96E-13	28334899
LPL	rs894210	chr8:19865843	A/G	HDL	33.1	35.4	54.9	88.8	0.0284	0.0039	4.39E-13	28334899
8q24.13 (intergenic)	rs2980888	chr8:126507308	T/C	TG	28.5	29.5	29.0	10.6	0.0506	-0.0506	4.88E-08	25961943
ABCA1	rs1883025	chr9:107664301	T/C	HDL	25.5	22.9	24.5	33.3	-0.0279	0.0042	2.38E-11	21909109
APOA5	rs651821	chr11:116662579	C/T	TG	30.0	28.8	6.7	15.1	0.1550	0.0090	1.11E-64	28334899
APOA5	rs662799	chr11:116663707	G/A	HDL	30.0	29.2	7.9	12.1	-0.0473	0.0040	6.56E-32	28334899
LIPC	rs2070895	chr15:58723939	A/G	HDL	43.0	39.5	22.7	51.3	0.0374	0.0037	3.77E-24	28334899
CETP	rs12446515	chr16:56987015	T/C	TCHL	16.6	15.9	31.9	14.0	5.2555	0.8088	8.73E-11	28334899
**CETP**	**rs821840**	**chr16:56993886**	**G/A**	**HDL**	**16.8**	**-**	**-**	**-**	**0.0656**	**0.0048**	**1.86E-41**	**28334899**
TXNL4B	rs77303550	chr16:72079657	T/C	LDL	25.8	27.1	20.0	16.8	-0.0333	0.0058	9.91E-09	28334899
TXNL4B	rs217184	chr16:72105965	C/T	TCHL	26.2	27.6	20.3	15.5	-4.1030	0.6837	2.06E-09	28334899
SPC24	rs34774090	chr19:11253886	CA/C	LDL	28.7	26.3	15.2	37.3	-0.0338	0.0057	3.45E-09	28334899
DOCK6	rs17699089	chr19:11343795	G/A	TCHL	27.2	27.6	12.4	17.7	-3.8794	0.6685	6.79E-09	28334899
APOE	rs1065853	chr19:45413233	T/G	LDL	6.3	8.1	8.3	11.8	-0.1615	0.0103	3.40E-54	28334899
				TCHL					-13.1251	1.2272	1.72E-26	28334899

APOE	rs483082	chr19:45416178	T/G	TG	16.9	19.3	22.6	44.8	0.0706	0.0112	2.71E-10	25961943
APOC1	rs66626994	chr19:45428234	A/G	HDL	10.0	10.5	15.5	12.3	-0.0396	0.0066	1.76E-09	25961943
SAMM50	rs3761472	chr22:44368122	G/A	ALT	41.1	38.9	16.7	16.9	0.0362	0.0081	7.23E-06	29562163
Known association at known loci (Asian-specific)												
TRAFD1	rs12231737	chr12:112574616	T/C	ALT	16.3	27.1	0.0	0.01	-0.0602	0.0107	2.09E-08	21909109
				AST					-0.0544	0.0075	3.23E-13	21909109
HECTD4	rs77768175	chr12:112736118	G/A	HDL	15.7	26.7	0.0	0.0	-0.0329	0.0050	5.34E-11	28334899
Missense variants (Asian-specific)												
GOT1	rs76850691	chr10:101157438	C/G	AST	1.3	1.1	0.0	0.0	-0.1510	0.0246	8.55E-10	24124411
PCSK9	rs151193009	chr1:55509585	T/C	LDL	1.3	0.9	0.0	0.0	-0.1827	0.0218	6.14E-17	26690388
				TCHL					-19.4039	2.5902	7.67E-14	26690388
APOB	rs13306194	chr2:21252534	A/G	LDL	12.4	13.4	0.11	0.06	-0.0280	0.0076	2.41E-04	26690388
				TCHL					-4.1917	0.9066	3.84E-06	26690388
APOA5	rs2075291	chr11:116661392	A/C	HDL	7.9	6.7	0.03	0.03	-0.0748	0.0067	2.08E-28	26690388
				TG					0.1931	0.0153	5.26E-36	26690388
CETP	rs2303790	chr16:57017292	G/A	HDL	4.6	3.3	0.01	0.0	0.1039	0.0087	1.35E-32	26690388
				TCHL					6.4120	1.4332	7.80E-06	26690388

EAF, Estimated Allele Frequency; KOR, Korean; gnomAD, The Genome Aggregation Database EA, East Asian; EUR (non-finnish), European; AFR, African; TG, triglyceride; LDL, Low-density lipoprotein cholesterol; HDL, high-density lipoprotein cholesterol; TCHL, Total cholesterol; AST, Aspartate aminotransferase; ALT, alanine aminotransferase. Bold-face represents no EAF information in gnomAD.

Table S6. Dataset for performance test of the study

Platform	# of samples [(1)]	# of SNPs (initial)	# of SNPs (QCed)	Cohort	Sample QC	SNP QC	Imputation	Reference (PMID)	Data analysis	
									Accuracy	Genomic coverage
Korea Biobank Array	6,949	833,535	805,769	Ansung & Ansan	low call rate (< 99%), excessive heterozygosity, cryptic first-degree relatives, and gender inconsistency	low quality SNP(Off target variants, Other categorized by SNPolisher), Hardy-Weinberg equilibrium P < 1e-6, genotype call rates < 95%	Pre-phasing (Shapeit v2), imputation (IMPUTE v2 with 1,000 Genomes project phase 3 reference)	-	O	O
Affymetrix Genome-wide Human SNP Array 5.0	6,949	500,568	352,228	Ansung & Ansan	low call rate (< 96%), excessive heterozygosity, cryptic first-degree relatives, and gender inconsistency	Hardy-Weinberg equilibrium P < 10-6, genotype call rates < 95%, MAF < 0.01	Pre-phasing (Shapeit v2), imputation (IMPUTE v2 with 1,000 Genomes project phase 3 reference)	Cho et al. Nature Genetics 2009 (19396169)	O	O
Illumina HumanExome BeadChip v1.1	5,793	242,901	77,204	Ansung & Ansan	low call rate (< 99%), excessive heterozygosity, cryptic first-degree relatives, and gender inconsistency	Hardy-Weinberg equilibrium P < 10-6, genotype call rates < 95%, monomorphic	Pre-phasing (Shapeit v2), imputation (IMPUTE v2 with 1,000 Genomes project phase 3 reference)	Park et al. Int. J. Genomics 2015 (26819946)	O	X
Exome sequencing (Illumina Hiseq 2000)	155	367,412	360,254	Ansung & Ansan	-	Hardy-Weinberg equilibrium P < 10-6, genotype call rates < 95%	-	Hwang et al. Genomics. 2015 (25535679)	O	X
Affymetrix Genome-wide Human SNP Array 6.0	3,695	906,600	646,062	HEXA	low call rate (< 95%), excessive heterozygosity, cryptic first-degree relatives, and gender inconsistency	Hardy-Weinberg equilibrium P < 10-6, genotype call rates < 95%, and MAF < 0.01	Pre-phasing (Shapeit v2), imputation (IMPUTE v2 with 1,000 Genomes project phase 3 reference)	Kim et al. Nature Genetics 2011 (21909109)	X	O
Korea Biobank Array	96*	833,535	582,287	Ansung & Ansan	low call rate (< 97%), excessive heterozygosity, cryptic first-degree relatives, and gender inconsistency	low quality SNP(Off target variants, Other categorized by SNPolisher), Hardy-Weinberg equilibrium P < 1e-6, genotype call rates < 95%, MAF < 1%, non-autosome	Pre-phasing (Shapeit v2, reference guided), imputation (IMPUTE v2 with 1,000 Genomes project phase 3 reference)	-	X	O
Illumina Global Screening Array	96	700,078	375,884	HEXA	low call rate (< 97%), excessive heterozygosity, cryptic first-degree relatives, and gender inconsistency	Hardy-Weinberg equilibrium P < 10-6, genotype call rates < 95%, MAF < 0.01, non-autosome	Pre-phasing (Shapeit v2, reference guided), imputation (IMPUTE v2 with 1,000 Genomes project phase 3 reference)	-	X	O
Axiom Biobank array	96	628,679	250,909	HEXA	low call rate (< 97%), excessive heterozygosity, cryptic first-degree relatives, and gender inconsistency	low quality SNP(Off target variants, Other categorized by SNPolisher), Hardy-Weinberg equilibrium P < 1e-6, genotype call rates < 95%, MAF < 1%, non-autosome	Pre-phasing (Shapeit v2, reference guided), imputation (IMPUTE v2 with 1,000 Genomes project phase 3 reference)	-	X	O
Axiom UK Biobank array	96	830,114	364,850	HEXA	low call rate (< 97%), excessive heterozygosity, cryptic first-degree relatives, and gender inconsistency	low quality SNP(Off target variants, Other categorized by SNPolisher), Hardy-Weinberg equilibrium P < 1e-6, genotype call rates < 95%, MAF < 1%, non-autosome	Pre-phasing (Shapeit v2, reference guided), imputation (IMPUTE v2 with 1,000 Genomes project phase 3 reference)	-	X	O
Axiom Precision Medicine Research Array	96	920,636	424,780	HEXA	low call rate (< 97%), excessive heterozygosity, cryptic first-degree relatives, and gender inconsistency	low quality SNP(Off target variants, Other categorized by SNPolisher), Hardy-Weinberg equilibrium P < 1e-6, genotype call rates < 95%, MAF < 1%, non-autosome	Pre-phasing (Shapeit v2, reference guided), imputation (IMPUTE v2 with 1,000 Genomes project phase 3 reference)	-	X	O
Illumina HumanOmni 1M	3,666	1,010,624	730,073	CAVAS	low call rate (< 99%), excessive heterozygosity, cryptic first-degree relatives, and gender inconsistency	Hardy-Weinberg equilibrium P < 10-6, genotype call rates < 95%, and MAF < 0.01	Pre-phasing (Shapeit v2), imputation (IMPUTE v2 with 1,000 Genomes project phase 3 reference)	Kim et al. Genomics 2013 (23147675)	X	O

[(1)] sample size after QC

*These samples are randomly selected from 6,945 Ansung & Ansan samples

HEXA, Health Examinee Study; CAVAS, Cardiovascular Association Study

Table S7. Demographic characteristics of KoGES

Characteristics	Total subject (n=211,715)		
	AAS	HEXA	CAVAS
**Number of participant**	10,030	173,357	28,338
**General information**			
Age (years) [a]	52.29 ± 8.93	53.10 ± 8.37	58.58 ± 9.38
Female, n (%)	5272 (52.56)	114063 (65.80)	17517 (61.81)
**Health habit**			
Smoking status, n (%)			
Non-smokers [a]	5808 (58.69)	125162 (72.88)	19893 (70.32)
Ex-smokers [a]	1539 (15.55)	25218 (14.68)	4254 (15.04)
Current smokers [a]	2549 (25.76)	21352 (12.43)	4143 (14.64)
Drinking status, n (%)			
Non-drinkers [a]	4596 (46.24)	87241 (50.75)	14476 (51.23)
Ex-drinkers [a]	652 (6.56)	6911 (4.02)	2026 (7.17)
Current drinkers [a]	4691 (47.20)	21352 (45.23)	11753 (41.60)
Regular exercise, n (%)[a]	NA	90381 (52.55)	8860 (31.34)
**Anthropometric measurements**			
Height (cm) [a]	159.98 ± 8.66	160.49 ± 8.03	157.83 ± 8.50
Weight (kg) [a]	63.01 ± 10.10	61.83 ± 9.92	60.92 ± 10.07
BMI (kg/m^2) [a]	24.57 ± 3.15	23.94 ± 2.91	24.40 ± 3.15
SBP (mmHg) [a]	121.69 ± 18.48	122.71 ± 15.47	126.28 ± 17.93
DBP (mmHg) [a]	80.34 ± 11.44	76.24 ± 10.03	79.41 ± 11.12
**Clinical examination**			
FPG (mg/dl) [a]	87.31 ± 21.41	95.18 ± 21.59	98.67 ± 24.16
TC (mg/dl) [a]	191.17 ± 35.83	197.45 ± 21.59	199.75 ± 37.33
TG (mg/dl) [a]	162.47 ± 104.96	126.88 ± 89.90	149.87 ± 100.31
HDL (mg/dl) [a]	44.64 ± 10.07	54.02 ± 12.94	45.36 ± 11.12
LDL (mg/d) [a]	-	-	-
**Prevalence ***			
Hypertension, n (%) [b]	3249 (33.88)	50984 (30.98)	12614 (40.10)
Type 2 diabetes, n (%) [c]	849 (8.76)	15157 (9.35)	3376 (10.29)
Obesity, n (%) [d]	4290 (43.03)	56857 (34.80)	11295 (40.16)

This table is adapted from table 1 of Kim et al. Int J Epidemiol. 2017;46(2):e20.

AAS, Ansan and Ansung study; HEXA, Health Examinee Study; CAVAS, Cardiovascular Association Study.

[a] Means±6 standard deviation (SD); [b] defined as either a dbp of ≥ 140 mmHg or ≥ 90 mmHg, respectively, or when participants self-reported the diagnosed diseases; [c] defined as either a fasting blood glucose level of ≥126 mf/dl or when participants self-reported the diagnosed diseases; [d] defined as body mass index ≥ 25 kg/m^2; [*] age-standardized prevalence rate in population-based studies was based on the 2014 mid-year resident population data.

Table S8. Demographic characteristics of the study population

Study	AAS	HEXA
Stage	Discovery	Replication
Study design	Population-based prospective	Population-based prospective
Sample size	6,949	6,000
Male/Female	3,450/3,499	2,256/3,744
Age (year) [a]	52.11 ± 8.86	53.60 ± 8.19
TC (mg/dl) [a]	192.44 ± 35.93	197.34 ± 35.38
HDL (mg/dl) [a]	44.58 ± 10.08	52.77 ± 12.53
LDL (mg/d) [a]	116.42 ± 32.21	120.06 ± 31.93
TG (mg/dl) [a]	164.14 ± 105.86	125.51 ± 85.76
AST (IU/L) [a]	29.75 ± 17.99	23.74 ± 14.02
ALT (IU/L) [a]	28.33 ± 22.67	22.84 ± 18.77
FPG (mg/dl) [a]	84.56 ± 10.23	90.31 ± 10.08

AAS, Ansan and Ansung study; HEXA, Health Examinee Study; TG, triglyceride; LDL, Low-density lipoprotein cholesterol; HDL, high-density lipoprotein cholesterol; TCHL, Total cholesterol; AST, Aspartate aminotransferase; ALT, Alanine aminotransferase; FPG, Fasting plasma glucose

[a]Means±6 standard deviation (SD)

Supplementary Note: The Korea Biobank Array: Design and Identification of Coding Variants Associated with Blood Biochemical Traits

Data description (2,579 Sequencing data used for designing KoreanChip)

- Korean Reference Genome(n=397)

Four hundred healthy volunteers were recruited to participate in the Korean Reference Genome project, and peripheral blood samples were collected. Written informed consent was obtained from all participants. Genomics DNA from the participants were whole-genome sequenced using Illumina HiSeq 2000 platform. Raw reads were aligned on hg19 reference genome using Burrows-Wheeler Aligner (BWA) with default parameters [1]. PCR duplicates were removed using Picard [2]. Resulting BAM files were pre-processed using Genome Analysis Toolkit (GATK) [3]. IndelRealigner was used for realigning reads near short indels and base quality was recalibrated. The pre-processed BAM files were analyzed to identify variants via SNP calling pipeline and LD-aware calling of Genome on the Cloud (GotCloud) pipeline [4]. Three hundred and ninety-seven unrelated samples were used for further analysis based on pairwise identify-by-state analysis of 400 samples. Average read depth per sample range from approximately 10 ~ 30x. As a result, about 20,700,000 variants were discovered.

- Korean samples from T2D-GENES consortium(n=1,087)

By the Type 2 Diabetes Genetic Exploration by Next- generation Sequencing in Ethnic Samples (T2D-GENES) Consortium [5], approximately 10,000 exomes from five ethnic groups were sequenced using Agilent SureSelect Human Exon v2 44M (Agilent Technologies, Santa Clara, CA) at the Broad Sequencing Center. A portion of the samples was from the KARE project [6],

including 538 unrelated type 2 diabetes samples and 579 control samples, and 1,087 samples were used for further analysis after sample quality control. Sequence data were analyzed using Picard, BWA, and GATK pipelines [1-3]. As a result, 500,821 autosomal variants were obtained from the 1,087 Korean Exome sequenced samples.

- Ansung and Ansan study(n=200) and Cardiovascular disease sequencing study(n=200)

Hundred healthy individuals with normal blood pressure level (Systolic Blood Pressure (SBP) 90~119, Diastolic Blood Pressure (DBP) 60~79) and 100 individuals with high blood pressure level (SBP >= 140, DBP >= 90) were randomly selected from the KARE project [6,7], and 200 cardiovascular disease patients were selected from the Genomics Research in Cardiovascular Disease (GenRIC) [8,9]. Peripheral blood samples were collected from the individuals with written informed consent. Genomic DNA was then extracted from the blood samples, and each DNA sample was used for exome enrichment using the Agilent SureSelect Human Exon v2 44M (Agilent Technologies, Santa Clara, CA). All samples were sequenced using Illumina HiSeq 2000 platform. All samples were analyzed together by BWA (alignment on hg19 reference genome), Picard (remove PCR duplicates), and GATK (realignment, recalibration and genotype calling) [1-3]. Average read depth was approximately 60x and approximately 367K variants were identified.

- Korean Children and Adolescents Obesity Cohort Study(n=692)

Study subjects, aged from 12 to 15 years old, were recruited from the Korean Children and Adolescents Obesity Cohort study [10]. Informed parental consents of enrolled children were

obtained. Genomic DNA was used for exome enrichment using the Agilent SureSelect Human Exon v4 71M (Agilent Technologies, Santa Clara, CA). All samples were sequenced using Illumina HiSeq 2500 platform. Raw data were analyzed by BWA (alignment on hg19 reference genome), Picard (remove PCR duplicates), and GATK (realignment, recalibration and genotype calling) [1-3]. As a result, approximately 726K variants were identified. Average read depth was 67.2x.

The data sources for each functional category in Korea Biobank Array (Table 1)

Korea Biobank Array has been designed using imputation-aware SNP selection and provides content modules for GWAS, human diseases, and biological functional variants. There are 833,535 SNPs and indel markers on the Korea Biobank Array. Similar to UK Biobank Axiom Array, available modules of specific interest in AxiomGD were adopted for Korea Biobank Array.

Tag SNPs for genome-wide coverage (600,294 markers)

Korea Biobank Array's core imputation grid consists of approximately 600K genome-wide SNPs shared in common with the conventional Affymetrix Biobank Array. Markers were selected using Affymetrix' imputation aware marker choice algorithms considering the MAF of 7.7M common variants (MAF > 1% in 2,579 Korean sequencing data including 397 WGS and 2,179 WES)

Coding variants of East Asian population (208,039 markers, primarily Missense)

208K coding variants were selected in East Asian populations for low (1%<EMAF<5%) and rare (1%>EMAF) frequency markers (using 504 EAS data of 1000 genomes project phase 3).

Functional contents (35,824 markers)

Korea Biobank Array contains the following Affymetrix' Axiom platform modules of variants based on reported GWAS signals and pharmacogenomic and metabolic phenotypes.

- Expression quantitative trait loci (eQTL) (16,690 markers)
- HLA and KIR region markers (6,659 and 1,546 markers, respectively)
- The set of Fingerprint markers (255 markers)
- NHGRI GWAS catalog Markers were chosen directly from the NHGRI Catalog of Published Genome-Wide Association Studies (8,136 markers)
- Pharmacogenetics/ADME category consists of markers from the Pharmaco-genomics Knowledgebase (2,037 markers)
- Y chromosome markers (807 markers)

Future plan of Korea Biobank Array project

Korea Biobank array (KBA) project will provide the largest East Asian genomic data containing both directly genotyped common and rare variants. Previously, UK Biobank produced a half million samples of genome data using UK BiLEVE array, a prototype, for about 50,000 samples and UK Biobank array for about 450,000 samples [11]. Those two arrays share about 95% of its contents. As a similar strategy used for UK Biobank genome data production, KBA was regarded as a prototype (v1.0) and an updated KBA (v1.1) was designed

by excluding variants with poor genotype clusters and including additional tagging variants for less common variants (MAF 1~5%) and variants in X chromosome. These two arrays share about 93% of its contents. In the KBA project based on KOGES cohorts, about 50,000 samples were genotyped using KBA v1.0 and genotyping of remaining about 150,000 samples using KBA v1.1 will be completed at the end of 2019.

To facilitate genomic researches using Korean chip, Korean chip consortium was established in June 2016. Since then, there are about 150 domestic researchers participating in the consortium and produced genotyped data of about 50,000 disease patients using the Korean chip. Korean chip consortium is expected to discover numerous genetic variants associated with various diseases, such as diabetes, cardiac diseases, and cancers, of Koreans. The discovered variants will be valuable scientific evidence on precision medicine in Koreans.

Contents summary of KoreanChip v1.1

Category	Description	# of variants
GWAS markers	Tag SNPs for genome-wide coverage (Autosomal and X chromosome)	595,957
	NHGRI GWAS catalog	7,635
Function variants	nonsynonymous SNPs, Indels, etc.	196,944
	eQTL	16,097
Pharmacogenomic	Absorption, distribution, metabolism, and excretion (ADME) markers	1,818
Immune related	Human Leukocyte Antigen (HLA) region	6,605
	Killer immunoglobulin-like receptor (KIR) region	1,495
Fingerprint	Fingerprint/sample tracking	248
MT/Y Chromosome	Common mitochondrial DNA variants	178
	Y chromosome markers	806
Total # of variants		827,783

Supplementary References

1. Li, H. & Durbin, R. Fast and accurate short read alignment with Burrows-Wheeler transform. Bioinformatics 25, 1754–1760 (2009).
2. Picard: http://broadinstitute.github.io/picard/
3. DePristo, M. A. et al. A framework for variation discovery and genotyping using next-generation DNA sequencing data. Nat. Genet. 43, 491–498 (2011).
4. Jun G et al. An efficient and scalable analysis framework for variant extraction and refinement from population scale DNA sequence data, Genome Research 2015
5. Fuchsberger et al. The genetic architecture of type 2 diabetes. Nature 2016
6. Cho et al. A large-scale genome-wide association study of Asian populations uncovers genetic factors influencing eight quantitative traits. Nature Genetics 2009
7. Hwang et al. Combinatorial approach to estimate copy number genotype using whole-exome sequencing data. Genomics 2015
8. Lee JY et al. A genome-wide association study of a coronary artery disease risk variant. J. Hum. Genet. 2013
9. Lee et al. Genome-based exome sequencing analysis identifies GYG1, DIS3L and DDRGK1 are associated with myocardial infarction in Koreans. J. Genet. 2017
10. Moon et al. Whole-exome sequencing study reveals common copy number variants in protocadherin genes associated with childhood obesity in Koreans. Int. J. Obes (Lond). 2017
11. Wain et al. Novel insights into the genetics of smoking behaviour, lung function, and chronic obstructive pulmonary disease (UK BiLEVE): a genetic association study in UK Biobank. Lancet Respiratory Medicine 2015
